# Metabolic engineering of *Escherichia coli* for shikimate pathway derivative production from glucose–xylose co-substrate

**DOI:** 10.1038/s41467-019-14024-1

**Published:** 2020-01-14

**Authors:** Ryosuke Fujiwara, Shuhei Noda, Tsutomu Tanaka, Akihiko Kondo

**Affiliations:** 1 Department of Chemical Science and Engineering, Graduate School of Engineering, Kobe University, 1-1 Rokkodai, Nada, Kobe, 657-8501 Japan; 2 Center for Sustainable Resource Science, RIKEN, 1-7-22 Suehiro-cho, Tsurumi-ku, Yokohama, Kanagawa 230-0045 Japan; 3 Graduate School of Science, Technology and Innovation, Kobe University, 1-1 Rokkodai, Nada, Kobe, 657-8501 Japan

**Keywords:** Metabolic engineering, Applied microbiology

## Abstract

Glucose and xylose are the major components of lignocellulose. Effective utilization of both sugars can improve the efficiency of bioproduction. Here, we report a method termed parallel metabolic pathway engineering (PMPE) for producing shikimate pathway derivatives from glucose–xylose co-substrate. In this method, we seek to use glucose mainly for target chemical production, and xylose for supplying essential metabolites for cell growth. Glycolysis and the pentose phosphate pathway are completely separated from the tricarboxylic acid (TCA) cycle. To recover cell growth, we introduce a xylose catabolic pathway that directly flows into the TCA cycle. As a result, we can produce 4.09 g L^−1^ *cis*,*cis*-muconic acid using the PMPE *Escherichia coli* strain with high yield (0.31 g g^−1^ of glucose) and produce l-tyrosine with 64% of the theoretical yield. The PMPE strategy can contribute to the development of clean processes for producing various valuable chemicals from lignocellulosic resources.

## Abstract (web summary)

In lignocellulose biomass, microbes prefer consuming glucose over xylose, which affects target compound production. Here, the authors achieve simultaneous utilization of glucose and xylose for target chemical production and cell growth, respectively, and realize high-level production of shikimate pathway derivatives.

## Introduction

Research on the microbial production of useful materials has gained interest and attention as an alternative production method of petrochemical products^1–3^. Metabolic engineering has made great contributions to advances in bioproduction^4–6^ on the premise of using only particular sugars as carbon sources^7–10^. Lignocellulosic biomass, which does not compete with global food supplies, is a promising raw material for bioproduction^11,12^. Glucose, existing as a component of cellulose, is the most common monosaccharide obtained from woody biomass^13^. Hemicelluloses, which contain xylose as the main constituent, represent ~20–40% of lignocellulosic biomass^14^. The co-utilization of glucose and xylose in lignocellulosic biomass is essential for the economically feasible production of biofuels and chemicals^15–17^. One problem with the use of mixed sugars is carbon catabolite repression (CCR)^18,19^. When *E. coli* is cultivated with glucose–xylose mixtures, glucose is preferentially consumed before xylose because of CCR, which decreases the production rate and/or titer; thus, it is necessary to repress or avoid CCR to efficiently utilize glucose–xylose mixtures^20^.

A successful strategy for relieving CCR is the knockdown of the phosphotransferase system (PTS) and the application of adaptive evolution to improve sugar co-utilization^21^. Disrupting l-arabinose transcriptional regulator, which acts as a repressor of xylose catabolite enzymes, is another strategy to eliminate CCR^22^. Wang et al. engineered an *E. coli* strain that utilized glucose–xylose mixtures for methyl ketone production^23^. To inhibit CCR, glucose uptake was reduced by disrupting the gene encoding glucose-specific PTS enzyme II component (*ptsG*), and xylose uptake was enhanced by the expression of a xylose transporter and xylose isomerase, which are encoded by *xylA* and *xylF*, respectively. This simple modification of metabolism allowed the strain to simultaneously use both the sugars, but it caused decreases in productivity and yield.

The two-strain co-culture system is another method for efficiently utilizing glucose–xylose mixtures^24,25^. Zhang et al. used a co-culture system with two metabolically engineered strains to increase the production of *cis,cis*-muconic acid (MA)^25^. MA, a shikimate pathway derivative, is a valuable compound that is a precursor of important chemical compounds, such as adipic acid and terephthalic acid, and its production has recently attracted attention^7,26–30^. The glucose-consuming strain synthesized 3-dehydroshikimic acid (DHS) and secreted it in culture medium, whereas the other xylose-consuming strain converted DHS to MA. Co-culture using strains assimilating each specific sugar resulted in high production of MA from mixed sugars, thus successfully avoiding CCR. However, in fermentation using a two-strain co-culture, several problems, such as complicated operations and difficult handling arise.

**Fig. 1:**
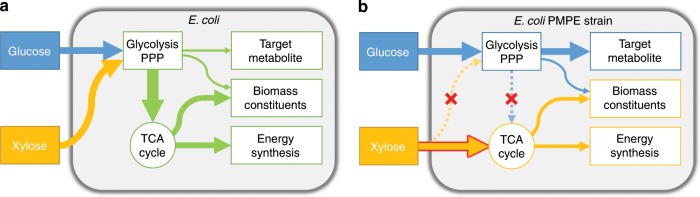
The concept of parallel metabolic pathway engineering. **a** Glucose and xylose carbon fluxes in common strains. Glucose and xylose are catabolized by the same pathways, glycolysis and the pentose phosphate pathway, and a large amount of the liberated carbon is used to synthesize biomass constituents and produce energy. **b** Glucose and xylose carbon fluxes in the engineered parallel metabolic pathway strain. Metabolic pathways of glucose and xylose do not intersect, making them metabolically parallel. Blue, yellow, and green arrows represent carbon flow from glucose, xylose, and both, respectively. The red-bordered yellow arrow represents the introduced exogenous xylose catabolite pathway. The thickness of arrows represents the proportion of carbon flow. Red crosses and dotted lines indicate the disruption of metabolic pathways.

In bioproduction, another common problem is that carbon leaks into pathways other than the target pathway (Fig. 1a)^31^. Phosphoenolpyruvate (PEP), pyruvate (PYR), and acetyl-CoA are important intermediates of target chemicals as well as tricarboxylic acid cycle (TCA cycle) components^4^. Hence, the biosynthetic pathways of chemicals derived from PEP, PYR, and acetyl-CoA compete for carbon flux with the TCA cycle, leading to decreased product yields^32^. However, the disruption of the TCA cycle or carbon flux into the cycle results in metabolic imbalance and negative effects on cell growth^33–35^. This means that target chemical production and cell growth are in an inverse relationship. As one solution, a switching system named metabolic toggle switch between the TCA cycle and target chemical production pathway was developed^32^. To toggle between cell growth and production, the expression of enzymes related to acetyl-CoA metabolism (*gltA*; citrate synthase) was controlled by transcriptional regulation. Using this system, isopropanol production was improved by as much as 3.7 times relative to that in the parental strain level. Although metabolic toggle switching is a useful tool for redirecting metabolic pathways, the ON/OFF timing of toggle switches (i.e., adding an inducer to the culture) needs to be optimized.

In this study, we propose parallel metabolic pathway engineering (PMPE) for co-utilizing glucose–xylose without decreasing growth ability. Glycolysis and the pentose phosphate pathway (PPP) are completely separated from the TCA cycle, and carbon supply from these pathways (i.e., PEP, PYR, and acetyl-CoA) is completely blocked from the TCA cycle. To recover cell growth (i.e., supply TCA cycle intermediates), a xylose catabolic pathway that directly flows into the TCA cycle without interfering glycolysis and PPP is introduced (Fig. 1). As a result, glucose is used mainly for target chemical production, and xylose is only used for cell growth. PEP is the most important precursor derived from glucose via glycolysis for chemical production. PEP is also a starting metabolite of the shikimate pathway as well as a precursor of the TCA cycle. Shikimate pathway-derived chemicals such as MA are among the most suitable targets for PMPE strains because they cause no carbon leaks from glycolysis into the TCA cycle. The highest yields of MA reported till date are 0.21 and 0.35 g g^−1^ of glucose in batch culture and complicated fed-batch culture, respectively^25,26,29^. Although some researchers partially disrupted the pathways connecting glycolysis and PPP to the TCA cycle to prevent carbon loss from the TCA cycle^25,29,36^. there are no reports in which these pathways were completely removed.

In many organisms, xylose is catabolized by the isomerase and *oxo*-reductive pathways into d-xylose 5-phosphate, which then enters PPP^37^. To construct a parallel metabolic pathway (PMP), we focused on the Dahms pathway. *Caulobacter crescentus*, an oligotrophic bacterium, catabolizes xylose via an alternate pathway^38^. The Dahms pathway in *C. crescentus* directly produces PYR and glyoxylate from xylose without glycolysis and PPP (Supplementary Fig. 1). In the Dahms pathways, each reaction step has a higher negative change of Gibbs energy than the isomerase and *oxo*-reductive pathways, which indicates high thermodynamic favorability^37^. Chemical production using the Dahms pathway has been successfully demonstrated with high yields^39,40^; however, there is no report regarding the use of this pathway for cell growth and maintenance. We assume that the Dahms pathway, which can provide PYR and glyoxylate from xylose as a carbon source, will be suitable for growth recovery in cells in which glycolysis and the TCA cycle cannot supply the necessary components.

To prove our concept, we produce MA using the PMPE *E. coli* strain. We modify the metabolic pathway of *E. coli* so that only glucose could be used for MA production, and we introduce the Dahms pathway to restore cell growth. We efficiently produce MA from glucose–xylose mixtures and investigate the fractional contribution of these sugars via ^13^C-metabolic analysis. To confirm the versatility of PMPE, we demonstrate the production of l-tyrosine which is another shikimate pathway derivative and 1,2-propanediol.

## Results and discussion

### PMP design

Our final goal in the present study was to construct an engineered strain that can simultaneously assimilate both glucose and xylose and produce target chemicals with high yields. With this aim, we devised a concept, PMPE. In PMPE, the pathway that produces target chemicals and another pathway that supplies metabolites essential for cell growth and maintenance are completely separated. These two pathways are metabolically parallel in the same strain and do not interfere with each other regarding metabolism (Fig. 1b). Since there is a larger amount of glucose than xylose in nature, we selected glucose as a substrate for producing target chemicals, and xylose is used in the pathway that synthesizes essential metabolites.

Regarding potential parent strains, we chose the previously developed *E. coli* CFT5 strain^41^. In CFT5, obtained from ATCC 31882, two genes encoding PYR kinase (*pykA* and *pykF*) were disrupted and the endogenous PTS was replaced with the galactose permease/glucokinase system (GalP/Glk system). These modifications improved PEP availability, and as a result, CFT5 produced various shikimate pathway derivatives at high yields from glucose supplemented in yeast extract. When CFT5 was cultured in M9 minimal medium containing glucose, xylose, or a glucose–xylose mixture, CFT5 did not grow (Supplementary Fig. 2a). Alternatively, the growth of the CFT5 strain was recovered in medium containing malate, which is a TCA cycle intermediate (Supplementary Fig. 2b). While the cell growth recovered by adding malate, it needed a very long lag phase, about 2 days. The gene expression patterns of these strains were also evaluated (Supplementary Discussion 1).

These results suggest that carbon flux from glucose into the TCA cycle is not sufficient to support the growth of the CFT5 strain. The Dahms pathway can produce PYR and glyoxylate, and glyoxylate will be immediately converted to malate via the glyoxylate shunt. Therefore, additional PYR and glyoxylate will be provided by the Dahms pathway.

### Introducing the Dahms pathway leads to growth on glucose

The exogenous xylose catabolic pathway for recovering the cell growth of the CFT5 strain needs to have the following two features: (i) ability to synthesize the essential metabolites of the TCA cycle from xylose and (ii) independence from both glycolysis and PPP. We focused on the Dahms pathway that synthesizes PYR and glyoxylate from xylose without using glycolysis/PPP-related enzymes. In the Dahms pathway, xylose dehydrogenase (*xdh*) from *C. crescentus* converts xylose to xylonolactone. Xylonolactonase (*xylC*) from *C. crescentus* converts xylonolactone to xylonate. Xylonate is converted to 2-dehydro-3-deoxy-d-xylonate (DHDOX) by endogenous xylonate dehydratase (*yjhG*), and then DHDOX is converted to PYR and glycolaldehyde by endogenous DHDOX aldolase (*yjhH*) (Supplementary Fig. 1). Glycolaldehyde is metabolized to glyoxylate, after which it enters the TCA cycle.

**Fig. 2:**
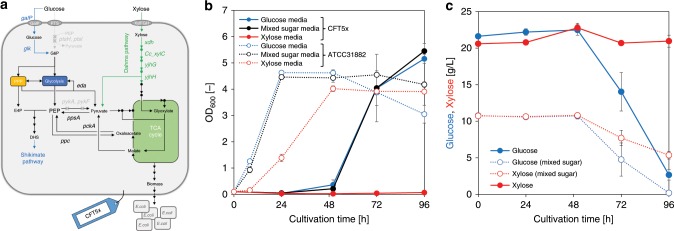
Strain construction and cell growth examination. **a** Metabolic design of CFT5x. **b** Bacterial cell growth. Blue, black, and red symbols indicate the growth of the CFT5x strain and ATCC31882 cultured in medium supplemented with glucose, a glucose–xylose mixture, and xylose, respectively. **c** Glucose and xylose consumption. Filled blue symbols indicate glucose consumption in glucose-containing medium. Filled red symbols indicate xylose consumption in xylose-containing medium. Open blue and open red symbols indicate glucose and xylose consumptions in mixed sugar-containing medium, respectively. G6P glucose-6-phosphate; PEP phosphoenolpyruvate; E4P erythrose 4-phosphate; DHS dehydroshikimate; and Gluconate-6P gluconate-6-phosphate. The data are presented as the average of three independent experiments, and error bars indicate standard errors. Source data underlying Fig. 2b, c are provided as a Source Data file.

A strain, CFT5x, harboring *xdh*, *xylC*, and an endogenous *yjhHG* expression plasmid (Fig. 2a), was cultured in M9 minimal medium with glucose, xylose, or a glucose–xylose mixture. When xylose-containing medium was used, CFT5x did not grow (Fig. 2b, c), suggesting that supplying metabolites synthesized from glycolysis or PPP was not sufficient for growth using xylose as the sole carbon source. Unexpectedly, CFT5x grew in M9 minimal medium containing glucose as the sole carbon source, suggesting that introducing the Dahms pathway may activate carbon flow into the TCA cycle from glucose but not xylose. We examined the effect on the cell growth by expressing each enzyme of Dahms pathway, Xdh, CcxylC, and YjhHG in CFT5. As a result, only the strain expressing Xdh could grow in M9 minimal medium containing glucose as the sole carbon source (Supplementary Fig. 3). However, Xdh has no or little enzymatic activity for glucose or glucose-6-phosphate as substrates^38^. Thus, 6-phosphogluconate is one of the possible candidates.

### Complete disruption of the metabolic connection

**Fig. 3:**
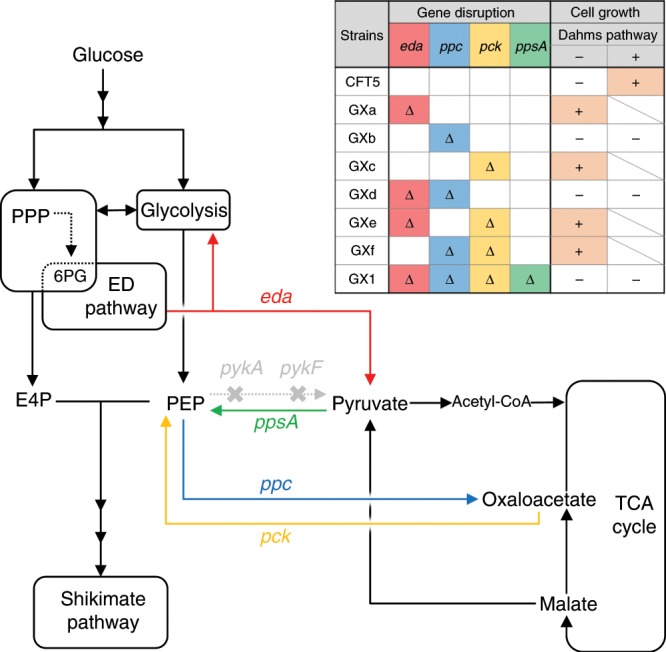
Metabolic pathway and growth of CFT5-derived strains. Δ indicates gene disruption. Orange highlighted + indicates that the strain grew in M9 minimal medium with glucose as the sole carbon source. ED pathway Entner–Doudoroff pathway; PEP phosphoenolpyruvate; E4P erythrose 4-phosphate; and 6PG 6-phosphogluconate.

One hypothesis of the mechanism of regaining cell growth by expressing Xdh is that Xdh activate the Entner–Doudoroff (ED) pathway. In the ED pathway, 6-phosphogluconate is converted to 2-keto-3-deoxygluconate-6-phosphate by phosphogluconate dehydratase (EDD, EC:4.2.1.12), then it is converted to PYR and glyceraldehyde-3-phosphate by 2-keto-3-deoxygluconate-6-phosphate aldolase, which is encoded by *eda*. If Xdh has dehydrogenase activity for 6-phosphogluconate as substrate, it is thought that ED pathway would be activated. Therefore, we constructed an *eda*-deficient strain GXa. Surprisingly, the GXa strain regained cell growth in a minimal medium containing glucose in the absence of the Dahms pathway (Fig. 3). This result suggests that the disruption of *eda* may activate other pathways or provide TCA cycle intermediates. In GXa, *ppc* and *pck* which coding PEP carboxylase (Ppc) and PEP carboxykinase (Pck), remained intact. Ppc and Pck catalyze the reaction converting PEP to OAA and the reverse reaction (PEP–OAA interconversion). It is thought that the balance of PEP–OAA interconversion may have been altered by the disruption of the ED pathway. The GXb strain (*ppc* disruption) did not grow in M9 minimal medium, but the GXc strain (*pck* disruption) regained cell growth in minimal medium. The disruption of *ppc* resulted in a loss of cell growth (GXb), but the GXf strain (with *ppc* and *pck* disruption) could grow. Another *pck-*disrupted strain, GXe (with *eda* and *pck* disruption), exhibited cell growth. Alternatively, the GXd strain (with *ppc* and *eda* deletion) exhibited no cell growth (Supplementary Fig. 4a, b).

In wild-type *E. coli*, Ppc and Pyk regulate metabolism between glycolysis and the TCA cycle^33^. In the *pyk*-knockout strain, the activities of Ppc and Pck are higher than those in the wild-type strain^42^. CFT5 also features *pykF* and *pykA* disruptions; moreover, it was believed that PEP–OAA interconversion was activated in CFT5-derived strains. Ppc and Pck activity, i.e., PEP–OAA interconversion, depends on the concentration of metabolites in the TCA cycle (i.e., PEP, OAA, acetyl-CoA, and malate), and it is regulated by these metabolites^43,44^. Figure 3 suggests that the loss of the growth of the CFT5 and GXb strains was attributable to the regulation of central metabolism including the regulation of Pck by the metabolites rather than insufficient carbon supply. Therefore, we believed that the disruption of these two metabolic pathways (ED pathway and PEP–OAA interconversion) is necessary to completely block carbon flow from glycolysis and PPP into the TCA cycle. In addition, to eliminate unexpected changes in metabolic regulation, all junctions between glycolysis and the TCA cycle need to be disrupted.

We generated the GX1 strain (with *eda*, *ppc*, *pck*, and *ppsA* disruptions). *ppsA* encodes PEP synthase, which converts PYR to PEP. The GX1 strain did not grow in M9 minimal medium containing glucose. To identify the necessary TCA cycle metabolites for cell growth, the GX1 strain was cultured in PYR-supplemented medium (M9P medium) or PYR- and malate-supplemented media (M9PM medium). It grew in M9PM medium but not M9P medium (Supplementary Fig. 5). These results indicate that the GX1 strain has a shortage of citrate, which is the starting material of the TCA cycle, because of the disruption of the oxaloacetate-generating reaction from PEP. In M9PM medium, supplemented malate is converted to oxaloacetate by malate dehydrogenase, which covers the lack of oxaloacetate supply.

### Growth of the GX1x strain by introducing the Dahms pathway

**Fig. 4:**
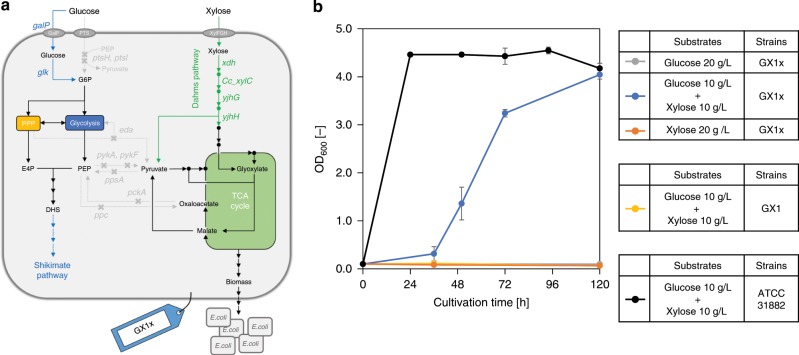
Examination of the growth of the strain in which the Dahms pathway was introduced. **a** Metabolic design of the GX1x strain. **b** Bacterial cell growth in minimal medium. Gray, blue, and orange symbols indicate the growth of the GX1x strain cultured in medium containing only glucose, a glucose–xylose mixture, and xylose alone, respectively. Yellow symbols indicate the growth of the GX1 strain (i.e., a strain lacking the Dahms pathway) cultured in medium containing a glucose–xylose mixture. Black symbols indicate the growth of the ATCC31882 (control strain) cultured in medium containing a glucose–xylose mixture. G6P glucose-6-phosphate; PEP phosphoenolpyruvate; E4P erythrose 4-phosphate; and DHS dehydroshikimate. The data are presented as the average of three independent experiments, and error bars indicate standard errors. Source data underlying Fig. 4b are provided as a Source Data file.

To regain cell growth, we introduced the Dahms pathway into the GX1 strain. GX1x strain equipped with the Dahms pathway (Fig. 4a) grew in M9 minimal medium with a glucose–xylose mixture as the carbon source, and maximum OD_600_ reached at the same level of ATCC31882 (Fig. 4b). Conversely, the strain did not grow in media containing glucose or xylose alone. Carbon from glucose could not flow into the TCA cycle, which leads to deficiencies of the essential metabolites and energy necessary for cell growth. In the medium containing glucose–xylose mixture, the Dahms pathway supplied PYR and glyoxylate from xylose as carbon sources, thereby restoring cell growth. It was thought that the GX1x strain did not grow in M9 minimal medium containing only xylose because of the insufficient activity of the endogenous xylose catabolic pathway (i.e., *xylAB*). We constructed the strain overexpressing XylA and XylB derived from GX1x. Contrary to expectations, this strain did not grow in M9 minimal medium with xylose as a sole carbon source. Furthermore, the strains derived from CFT5 overexpressing Dahms pathway enzymes (Xdh, CcxylC, YjhH, and YjhG) and/or enzymes of the endogenous xylose catabolic pathway (XylA and XylB) also did not grow in the same condition (Supplementary Fig. 6). In this strain, while PYR and glyoxylate were supplied via Dahms pathway, these metabolites would have been converted to intermediates of glycolysis and could not be used in the TCA cycle. Additionally, we analyzed the metabolome in PMPE strain. As a result, PMPE strain considerably accumulated PEP and had been enhanced with PPP. These phenotypes are suited for the production of shikimate pathway derivatives (Supplementary Discussion 2, Supplementary Figs. 14, 15).

### MA production from glucose–xylose mixture in the PMPE strain

**Fig. 5:**
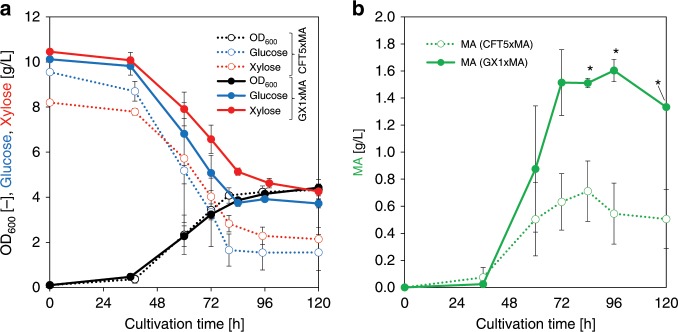
Culture profiles of *cis,cis*-muconic acid (MA)-producing strains in M9 minimal medium. **a** Black, blue, and red symbols indicate bacterial cell growth, glucose consumption, and xylose consumption, respectively. **b** Green symbols indicate the produced amounts of MA. All open and filled symbols indicate the results for the CFT5xMA and GX1xMA strains, respectively. The data are presented as the average of three independent experiments, and error bars indicate standard errors. *P* values were computed using the two-tailed Student’s *t*-test (*P < 0.05). Source data are provided as a Source Data file.

We attempted to produce MA via the shikimate pathway using a GX1x-derived strain. Protocatechuate (PCA) was synthesized from DHS by DHS dehydratase, PCA decarboxylase converted PCA to catechol (CA), and CA 1,2-dioxygenase produced MA from CA (Supplementary Fig. 7) with the highest theoretical yield among all MA synthetic pathways (0.68 g g^−1^ of glucose)^25^. Figure 5 illustrates the culture profiles of the GX1xMA strain (GX1 harboring an MA-producing plasmid) in M9 minimal medium. The GX1xMA strain produced 1.60 ± 0.08 g L^−1^ of MA after 96 h of cultivation with a yield of 0.30 ± 0.02 g g^−1^ of glucose after 72 h. CFT5xMA strain produced 0.71 ± 0.22 g L^−1^ of MA after 80 h of cultivation with a yield of 0.12 ± 0.05 g g^−1^ of glucose after 60 h. As a control of glucose and xylose co-utilizing strain, strain CTR2 was constructed by disrupting *ptsG* and *pheA* from ATCC31882. These genes are responsible for catabolite repression (*ptsG*) and production of l-phenylalanine competing with MA production (*pheA*), respectively. CTR2MA strain (CTR2 harboring an MA-producing plasmid) was cultured in M9 minimal medium and produced 0.53 ± 0.01 g L^−1^ of MA after 48 h (Supplementary Fig. 8). After 120 h cultivation of CFT5xMA and GX1xMA, 1.63 ± 0.24 and 1.67 ± 0.20 g L^−1^ of xylonate was accumulated, respectively, and the <0.05 g L^−1^ of acetate and lactate were accumulated in the medium (Supplementary Fig. 9). The production titer of MA from glucose of the GX1xMA strain were 2.3-fold and 3.0-fold higher than CFT5xMA strain and CTR2MA strain, respectively, and the yield was 2.6-fold higher than CFT5xMA. These findings suggest that the complete elimination of carbon flux from glycolysis to the TCA cycle improves MA production.

### ^13^C-metabolic analysis of the MA-producing strain

To analyze the metabolic flux of glucose and xylose in the PMPE strain, the GX1xMA strain was cultured in M9 minimal medium containing [U-^13^C]glucose and non-labeled xylose. After culturing, the levels of MA in the supernatant and that of five key amino acids (histidine, glycine, alanine, glutamic acid, and lysine) were analyzed via GC–MS. The initial optical density at 600 nm (OD_600_) of the cultures was 0.5, and cells were harvested for GC–MS analysis when OD_600_ reached 2.40 ± 0.47.

**Fig. 6:**
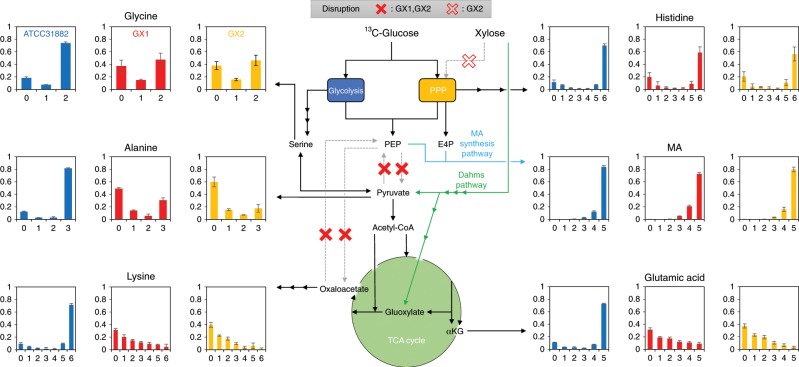
^13^C-metabolic analysis. Blue, red, and yellow bars indicate the mass isotopomer distributions of six metabolites [glycine, alanine, lysine, histidine, glutamic acid, and *cis,cis*-muconic acid (MA)] from tracer experiments with [U-^13^C]glucose and non-labeled xylose in ATCC31882xMA, GX1xMA, and GX2xMA, respectively. The vertical axis indicates the relative abundance. The horizontal axis is M+, which denotes the difference with a fully unlabeled isotopomer regarding the *m*/*z* of a mass fragment (fully unlabeled isotopomer, M+ = 0). The maximum value of M+ for each metabolite is the number of constituent carbons of the metabolite in the mass fragment. Red closed crosses indicate disrupted metabolic pathways in the GX1 and GX2 strains. Red open cross indicates the disrupted metabolic pathway in the GX2 strain. G6P glucose-6-phosphate; PEP phosphoenolpyruvate; E4P erythrose 4-phosphate; and αKG alpha-ketoglutarate. The data are presented as the average of three independent experiments, and error bars indicate standard errors. Source data are provided as a Source Data file.

Figure 6 presents the mass isotopomer distributions of metabolites. MA was completely labeled with ^13^C, indicating that all produced MA was derived from glucose. The carbon atoms in histidine were almost completely labeled with ^13^C. Histidine is synthesized from the PPP intermediate 5-phosphoribosyl diphosphate. This result indicates that carbon flux into PPP is only derived from glucose, and xylose was not used in this pathway. On the contrary, the proportions of carbon labeled with ^13^C in alanine, glutamic acid, and lysine were extremely low. It appears that these amino acids were synthesized from non-labeled xylose. These results support the hypothesis that xylose is mostly catabolized via the Dahms pathway even though the endogenous xylose catabolic pathway (i.e., xylose isomerase and xylulokinase) in *E. coli* was retained. To prevent accidental activation of the endogenous xylose isomerase pathway, we generated the GX2xMA strain, a GX1xMA-derived strain in which *xylA* (xylose isomerase) and *xylB* (xylulokinase) were disrupted. ^13^C-metabolic analysis of the GX2xMA strain revealed profiles similar to those of the GX1xMA strain (Fig. 6). This result suggested that the metabolites derived from the intermediates of glycolysis or PPP were not sufficiently synthesized from consumed xylose in the strain in which the Dahms pathway was introduced.

Contrary to our expectations, both labeled and non-labeled carbon atoms were detected in glycine and alanine. This was caused by the interconversion of serine and PYR^45^, as catalyzed by serine deaminase (SDA) encoded by *sdaA*. The interconversion of ^13^C-labeled serine and non-labeled PYR appears to be the junction between the glucose-dominated glycolysis/PPP and xylose-dominated TCA cycle. We generated the GX3xMA strain, which was derived by disrupting *sdaA* in the GX1xMA strain. The GX3xMA strain could not grow in M9 minimal medium containing the sugar mixture. These results indicate that although carbon metabolism mediated by SDA is essential for the growth of GX1-derived strain in M9 minimal medium, the ratio of carbon leakage was small in relation to the entire metabolism of *E. coli*.

### Optimization of MA production using PMPE strain

**Fig. 7:**
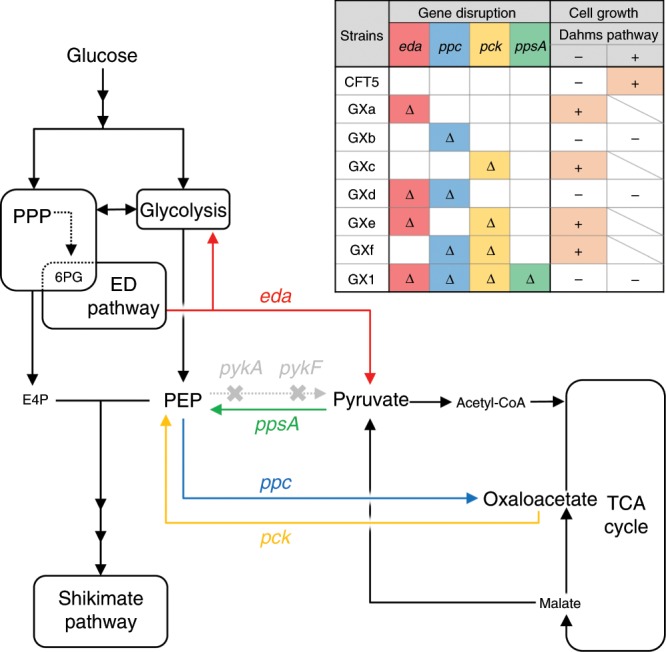
Culture profiles of the GX1xMA strain in M9 minimal medium containing 20 g L^−1^ sugar(s) and 5 g L^−1^ yeast extract. **a** Bacterial cell growth. **b** Produced amounts of MA. **c** Glucose consumption. **d** Xylose consumption. **e** Production yield of *cis,cis*-muconic acid (MA) from glucose (g of produced MA g^−1^ of consumed glucose). Red, orange, yellow, purple, and blue symbols indicate the results of cultivation in 0%, 25%, 50%, 75%, and 100% glucose medium, respectively, in **a**–**h**. **f** Culture profiles of the GX1xMA strain with the addition of CaCO_3_. The final sampling time is after 80 h cultivation. Blue, red, and yellow symbols indicate the glucose concentration, xylose concentration, and the produced amount of MA, respectively. The data are presented as the average of three independent experiments, and error bars indicate standard errors. Source data are provided as a Source Data file.

For MA production, the optimal glucose:xylose ratio was determined using the GX1xMA strain, which was cultivated using M9 minimal medium containing 20 g L^−1^ sugar(s) and 5 g L^−1^ yeast extract (M9Y medium) in shake flasks. The ratio of glucose to total sugars was 0%, 25%, 50%, 75%, or 100%. As expected, the cells did not grow well in medium containing glucose or xylose alone (Fig. 7a), corresponding to the results presented in Fig. 4b. Both glucose and xylose were needed for cell growth, and glucose content exceeding 50% was required. The highest specific growth rate was 0.136 h^−1^ in 75% glucose-containing medium (Supplementary Table 1).

Figure 7b presents the results of MA production. The GX1xMA strain produced 0.84, 1.94, and 3.13 g L^−1^ of MA after 48 h of cultivation in 25%, 50%, and 75% glucose-containing media, respectively. MA yields were similar among these glucose ratios (Fig. 7e). When using [U-^13^C]glucose and non-labeled xylose as substrates, the produced MA was completely labeled with ^13^C (Supplementary Fig. 10). Although the titer was increased by optimizing the glucose:xylose ratio, the yield decreased compared with that presented in Fig. 5 (titer, 1.60 ± 0.08 g L^−1^; yield, 0.30 ± 0.02 g g^−1^ of glucose). The maximum productivity of MA in 25%, 50%, and 75% glucose-containing media were 0.078, 0.072, and 0.114 g L^−1^ h^−1^, respectively. The intermediates of the MA synthesis pathway (PCA and CA) and shikimate pathway derivatives (l-phenylalanine, l-tyrosine, l-tryptophan, *p*-aminobenzoate, and *p*-hydroxybenzoate) were not detected in the medium, suggesting that these pathways were not rate-limiting steps.

We found that several organic acids were accumulated as by-products. As the xylose ratio in the medium was increased, considerable acetate and lactate productions were noted. In 25% glucose medium (glucose:xylose ratio is 1:3), 1.87 g L^−1^ of acetate and 1.29 g L^−1^ of lactate were produced after 48 h of cultivation (Supplementary Fig. 11). It is considered that the excessively consumed xylose overflowed to these organic acids. It is thought that the accumulation of acetate was caused by the activation of phosphate acetyltransferase (Pta) due to the accumulation of PEP. Pta converts PYR to acetyl phosphate and acetyl phosphate is converted to acetate by acetate kinase. PEP acts as a regulator of Pta and activates the production of acetyl phosphate and inhibit the reverse reaction^46^. The accumulation of lactate might be caused by a decrease in pH. In all conditions, pH was decreased under 5.0 after 72 h cultivation. The low pH condition activates lactate dehydrogenase which converts PYR to lactate^47^.

Furthermore, the accumulation of xylonate and glyoxylate increased in proportion to xylose concentration (Supplementary Fig. 11). In the case of medium containing xylose as the sole carbon source, 11.11 g L^−1^ of xylonate was produced after 72 h of cultivation. Xylonate accumulation is a common problem of Dahms pathway-mediated chemical production^39,40,48,49^, which decreases the yields of target compounds synthesized via this pathway. To reduce the accumulation of xylonate, overexpressing lactaldehyde dehydrogenase (AldA) would be one of the possible strategies. Glycolaldehyde synthesized via Dahms pathway is converted to glycolate by AldA and then glycolate is converted to glyoxylate by glycolate oxidase. Cabulong et al. increased glycolic acid production by overexpressing AldA^39^. In PMPE strains, it might be possible to improve the accumulation of xylonate that pulling the carbon flow from glycolaldehyde to glyoxylate by overexpressing AldA and glycolate oxidase. Another explanation of the glyoxylate accumulation is due to the shortage of acetyl-CoA. Glyoxylate and acetyl-CoA are converted to malate by malate synthase. Disruption of Pta and overexpression of malic enzymes coded by *maeB* (NADP-dependent malic enzyme) or *sfcA* (NAD-dependent malic enzyme), which converts malate to PYR, have the potential to solve glyoxylate accumulation. Furthermore, organic acids accumulation reduced the pH of the medium (4.59, 4.46, 4.85, and 4.98 in 0%, 25%, 50%, and 75% of glucose medium after 72 h of cultivation, respectively). Because pH control is important for MA production^29^, CaCO_3_ (10 g L^−1^) was added to the culture medium after 24 h of cultivation (Fig. 7f). Under this condition, the GX1xMA strain produced 4.09 ± 0.14 g L^−1^ of MA after 72 h of cultivation (Fig. 7f), representing a 1.31-fold increase compared with that produced without CaCO_3_. The yield reached 0.31 ± 0.003 g g^−1^ of glucose (80 h), which was improved to the same level as that in M9 minimal medium (Fig. 5). It is believed that carbons from glucose flowed to more downstream metabolites or that biomass turnover occurred via the degradation of shikimate pathway derivatives. To further increase the production and yield, it is necessary to disrupt metabolic pathways that compete with the shikimate pathway, and *aroE* and *ydiB*, which encode shikimate dehydrogenase, are promising candidates for such disruption^25,50^ (Supplementary Fig. 7).

We demonstrated MA production using the PMPE strain. Figures 5 and 7f show that CCR was almost relaxed, and glucose and xylose consumptions were simultaneously achieved. In many studies, xylose was catabolized via XylAB after the deregulation of CCR. Generally, xylose-metabolizing pathways catalyzed by XylAB have lower activity than glucose catabolism through glycolysis, as shown in Fig. 2. Our PMPE strain featuring the Dahms pathway consumed and efficiently metabolized xylose, leading to cell growth and high MA production. Moreover, the PMPE strategy simplifies the process compared with co-culture systems and the operation of fermentation using glucose–xylose mixtures.

### Production of other target metabolites using PMPE strain

**Fig. 8:**
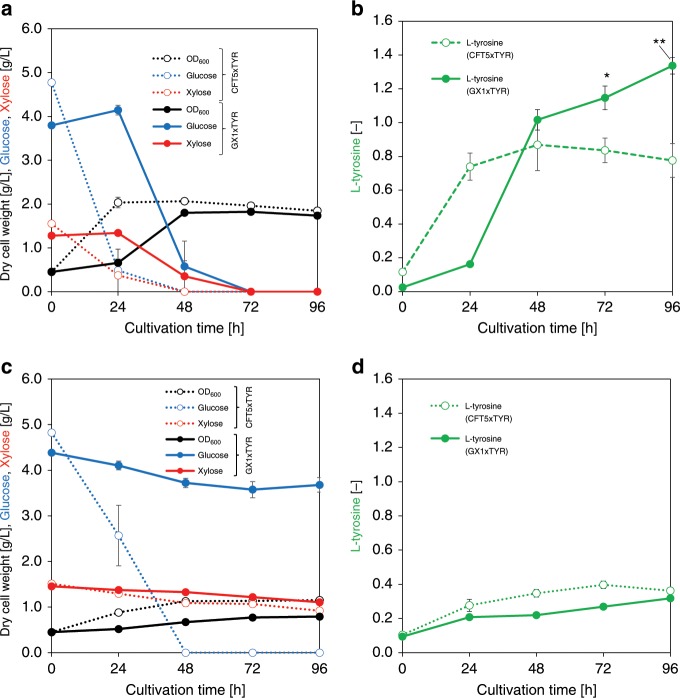
Cultures profiles of l-tyrosine-producing strains. **a**, **b** Aerobic condition. **c**, **d** Micro-aerobic condition. **a**, **c** Black, blue, and red symbols indicate bacterial cell growth, glucose consumption, and xylose consumption, respectively. **b**, **d** Green symbols indicate the produced amounts of l-tyrosine. All open symbols and filled symbols indicate the results for the CFT5xTYR and GX1xTYR strains, respectively. The data are presented as the average of three independent experiments, and error bars indicate standard errors. *P* values were computed using the two-tailed Student’s *t*-test (*P < 0.05; **P < 0.01). Source data are provided as a Source Data file.

To confirm the versatility of PMPE in the shikimate pathway, we attempted to produce l-tyrosine using the PMPE strain. The GX1xTYR strain, a GX1-derived strain harboring *tyrA*^fbr^ encoding feedback-resistant chorismate mutase/prephenate dehydrogenase, was cultivated in M9Y medium containing both 3.75 g L^−1^ of glucose and 1.25 g L^−1^ of xylose in shake flasks. In aerobic condition, GX1xTYR strain produced 1.34 ± 0.05 g L^−1^ of l-tyrosine after 96 h of cultivation, representing a 1.73-fold increase compared with that produced by the control strain CFT5xTYR (Fig. 8). The maximum productivity of l-tyrosine in CFT5xTYR and GX1xTYR were 0.026 and 0.021 g L^−1^ h^−1^, respectively. Xylonate, glyoxylate, lactate, and acetate were not accumulated in the medium after the cultivation of CFT5xTYR or GX1xTYR. The production yield reached 0.35 ± 0.01 g g^−1^ of glucose after 96 h of cultivation, reflecting a 2.15-fold increase compared with that using the CFT5xTYR strain and corresponding to 64% of the theoretical maximum without considering cell growth (0.55 g g^−1^ of glucose)^51^. In micro-aerobic condition, GX1xTYR strain and CFT5xTYR strain produced 0.32 ± 0.02 g L^−1^ (96 h) and 0.40 ± 0.02 g L^−1^ (72 h) of l-tyrosine, respectively, and the production yield in GX1xTYR reached 0.32 ± 0.10 g g^−1^ of glucose after 96 h of cultivation, reflecting a 5.22-fold increase compared with that using the CFT5xTYR (Fig. 8). These results suggested that PMPE is superior also in micro-aerobic conditions at the production yield, however, the production titer needs to be improved. In micro-aerobic condition, CFT5xTYR and GX1xTYR produced by-products (such as organic acids and ethanol) and the expression level of aerobic respiration control protein (ArcA, coded by *arcA*) was increased in micro-aerobic conditions (Supplementary Fig. 12a, b). ArcA down-regulates the transcription of genes in the TCA cycle^52^. To improve the productivity of PMPE strain in micro-aerobic conditions, the engineering of metabolic regulators including ArcA would be necessary. Juminaga et al. improved l-tyrosine production by deregulating bottlenecks (AroB and AroK) in its biosynthetic pathway^53^. They identified the bottlenecks by considering the expression way of genes. The optimized strain produced 2.17 g L^−1^ of l-tyrosine, which corresponds to 80% of the theoretical yield. Although the expression of shikimate pathway enzymes was not optimized in the PMPE strain, the achieved yield of l-tyrosine was high. It is believed that blocking carbon flow into the TCA cycle, which competes with the shikimate pathway, improves the yield of l-tyrosine production without optimizing enzyme expression in the shikimate pathway. To further improve l-tyrosine production, modifying the expression of shikimate pathway enzymes in the PMPE strain is a promising strategy. In our previous study, we suggested that a major reason for decreased yields of shikimate pathway derivatives was the loss of carbon for synthesizing metabolites branching from chorismate, which is an end-product of the shikimate pathway^29^. We solved this problem by expressing the protein fused chorismate synthase and isochorismate synthase in MA-producing strain in this study. This technique may be applicable to the PMPE strain for further improving l-tyrosine production. Additionally, we constructed the strain producing 1,2-propanediol as a non-shikimate pathway chemical derived from GX1x (GX1xPD). GX1xPD increased the production titer and the yield of 1,2-propanediol by 1.40-fold and 11.4-fold, respectively, compared to the control strain. (Supplementary Discussion 3, Supplementary Figs. 16, 17). These results suggested that PMPE is versatile for applying the production of various compounds including non-shikimate pathway derivatives.

We demonstrated that PMPE enables the efficient use of glucose–xylose co-substrate, and this strategy can be applied for producing shikimate pathway derivatives. The loss of cell growth caused by the complete interruption of carbon flow into the TCA cycle could be rescued by introducing the Dahms pathway. A high yield of MA was achieved using the PMPE *E. coli* strain because all glucose was used for target chemical production, whereas essential metabolites were supplied from xylose via the Dahms pathway. Poor growth and xylonate accumulation should be improved in the PMPE *E. coli* strain. Xylonate dehydratase in the Dahms pathway is a rate-limiting enzyme, and another isozyme, such as *E. coli yagF* with higher enzyme activity^39^ would be useful for efficient utilization of xylose. Glyoxylate accumulation is another issue to be solved. Using Weimberg pathway is an alternative option because this pathway supplies α-ketoglutarate from xylose without producing glyoxylate^54^.

By changing the disconnection point of metabolism, PMPE would be applicable to other pathways that branch from PYR, acetyl-CoA, and the upstream metabolites of glycolysis. Chemicals such as 1,2-propanediol are produced under anaerobic conditions^55,56^ to repress carbon flow into the TCA cycle, which causes a shortage of NADH supply^57^. We also demonstrated that PMPE can be applied to the production of 1,2-propanediol. Therefore, it is expected that the PMPE strain will significantly contribute to the production of other non-shikimate pathway chemicals with high yields.

## Methods

### Media

LB medium was used for pre-cultures. LB medium comprised the following (per liter): 10-g tryptone, 5-g yeast extract, and 5-g NaCl. M9 minimal medium was used for cell growth examination and MA production in 5-mL test tube-scale cultures. M9 minimal medium comprised the following (per liter): carbon source (20-g glucose, 10-g glucose, and 10-g xylose, 20-g xylose), 0.5-g NaCl, 17.1-g Na_2_HPO_4_·12H_2_O, 3-g KH_2_PO_4_, 1-g NH_4_Cl, 246-mg MgSO_4_·7H_2_O, 14.7-mg CaCl_2_·2H_2_O, 2.78-mg FeSO_4_·7H_2_O, 10-mg thiamine hydrochloride, 40-mg l-tyrosine, 40-mg l-tryptophan, and 100-mg l-phenylalanine (Tyr and Trp were included because ATCC31882 is auxotrophic for these amino acids and the CFT5 derivative strains are auxotrophic for Phe). M9P medium comprised M9 minimal medium supplemented with 10-mM sodium PYR. M9PM medium comprised M9P minimal medium supplemented with 10-mM malate. M9Y medium was used for MA production in flask-scale cultures. M9Y medium comprised M9 minimal medium supplemented with 5 g L^−1^ yeast extract. The ratio of glucose to the sugar mixture in M9Y medium was 0%, 25%, 50%, 75%, or 100%. In pH-controlled flask-scale cultures, 500 g L^−1^ of CaCO_3_ (dispersed in distilled water) was autoclaved and added to the cultures at a final concentration of 10 g L^−1^. M9Y medium without l-tyrosine supplementation was used to assess l-tyrosine production in flask-scale cultures. As needed, ampicillin and/or chloramphenicol were added to the initial medium at the final concentrations of 100 and 30 mg L^−1^, respectively.

### Culture conditions

Engineered strains were pre-cultured in 4 mL of LB medium for 1 day at 37 °C with shaking at 220 rpm in a 15-mL test tube. Each pre-culture medium was centrifuged at 12,000×*g* for 3 min, washed with M9 minimal medium without sugars, and used for inoculation. The pellet was suspended in fresh medium and seeded into the appropriate medium (supplemented with 0.1-mM isopropyl β-d-1-thiogalactopyranoside) at an initial OD_600_ of 0.1. These tube-scale cultures were incubated at 37 °C with shaking at 220 rpm. In micro-aerobic conditions, a screw tube was used and shaken at 150 rpm. In the pH-controlled tube-scale cultures, 10 g L^−1^ of CaCO_3_ was added to the cultures at 24 h after seeding. Flask-scale cultures were performed in 200-mL flasks with 10-mL working volume. In 1,2-propanediol production, tube-scale cultures were incubated at 30 °C in micro-aerobic condition.

### Strains and plasmid construction

The strains and plasmids used in this study are listed in Supplementary Data 6. *E. coli* NovaBlue competent cells (Novagen, Cambridge, MA, USA) were used for gene cloning. Polymerase chain reaction was performed using KOD FX Neo (TOYOBO, Osaka, Japan). Custom DNA oligonucleotide primers were synthesized by Invitrogen Custom DNA Oligos (Thermo Fisher Scientific, Tokyo, Japan) (Supplementary Data 7). Codon-optimized exogenous genes fragments (*xdh* and *XylC* from *Caulobacter crescentus*) were synthesized by the Invitrogen GeneArt Gene Synthesis service (Thermo Fisher Scientific).

pZE12-x was constructed as follows. Synthetic genes corresponding to *xdh* and *XylC* from *C. crescentus* were obtained from a commercial source (Invitrogen). The *xdh* gene fragment was amplified using PCR with the *xdh* synthetic gene as a template with the primer pair pZ-xdh Fw and pZ-xdh Rv. The amplified fragment was cloned between the *Kpn*I and *Hind*III sites of pZE12-MCS, and the resulting plasmid was designated as pZE12-xdh. The *XylC* fragment was amplified using PCR with the *XylC* synthetic gene as a template with the primer pair pZ-xylC Fw and pZ-xylC Rv. The amplified fragment was cloned between the *Hind*III and *EcoR*V sites of pZE12-xdh, and the resulting plasmid was designated as pZE12-xdh-xylC. *yjhHG* was amplified using PCR using *E. coli* MG1655 genomic DNA as a template with the primer pair yjhH Fw and yjhG Rv. The linearized fragment was amplified using inverse PCR using pZE12-xdh-xylC as a template with the primer pair pZ-xdh-xylC Inv. Rv and pZ-xdh-xylC Inv. Fw. This linearized fragment and the *yjhHG* fragment were circularized using an In-Fusion HD Cloning Kit (Takara), and the resulting plasmid was designated as pZE12-x.

pSAK-ZYc was constructed as follows. The *aroZ-aroY-catA* fragment was amplified using PCR using pZA23-ZYc as a template with the primer pair ZYc insert Fw and ZYc insert Rv. The linearized fragment was amplified using inverse PCR using pSAK as a template with the primer pair pZYc vector Fw and pZYc vector Rv. This linearized fragment and *aroZ-aroY-catA* fragment were circularized using the NEBuilder HiFi DNA Assembly Master Mix (New England Biolabs Japan, Tokyo, Japan), and the resulting plasmid was designated as pSAK-ZYc.

pSAK-*tyrA*^fbr^ was constructed as follows. The P_trc_ fragment was amplified using PCR using pTrcHisB as a template with the primer pair P_trc_ Fw and P_trc_ Rv. The linearized fragment was amplified using inverse PCR using pSAK as a template with the primer pair pSAK inv. Fw and pSAK inv. Rv. This linearized fragment and P_trc_ fragment were circularized using the NEBuilder HiFi DNA Assembly Master Mix, and the resulting plasmid was designated pSAK-P_trc_. A synthetic gene fragment corresponding to *E. coli tyrA*^fbr^ was obtained from a commercial source (Invitrogen)^41^. The *tyrA*^fbr^ fragment was amplified using PCR using the synthetic gene fragment as a template with the primer pair *tyrA*^fbr^ insert Fw and *tyrA*^fbr^ insert Rv. The linearized fragment was amplified using inverse PCR using pSAK-P_trc_ as a template with the primer pair pSAK-P_trc_ inv. Fw and pSAK-P_trc_ inv. Rv. This linearized fragment and the *tyrA*^fbr^ fragment were circularized using the NEBuilder HiFi DNA Assembly Master Mix, and the resulting plasmid was designated as pSAK-*tyrA*^fbr^.

pSAK-PD was constructed as follows. Synthetic gene corresponding to *Bs_mgsA* was obtained from a commercial source (Invitrogen). The *gldA* gene fragment was amplified using PCR with the *E. coli* MG1655 genomic DNA as a template with the primer pair gldA Fw and gldA Rv. The *Bs_mgsA*-*gldA* gene fragment was amplified using the *gldA* gene fragment and *Bs_mgsA* synthetic gene as templates with the primer pair Bs_mgsA fw and gldA Rv. The *fucO* gene fragment was amplified using PCR with the *E. coli* MG1655 genomic DNA as a template with the primer pair fucO Fw and fucO Rv. The *Bs_mgsA*-*gldA*-*fucO* gene fragment was amplified using the *Bs_mgsA*-*gldA* gene fragment and the *fucO* gene fragment as templates with the primer pair Bs_mgsA fw and fucO Rv. The amplified fragment was cloned between the *Kpn*I and *Hind*III sites of pSAK, and the resulting plasmid was designated as pSAK-PD.

pTΔeda was constructed as follows. The linearized fragment was amplified using inverse PCR using pTargetF as a template with the primer pair sgRNA eda Rv and sgRNA eda Fw. The amplified fragment was circularized using an In-Fusion HD Cloning Kit, and the resulting plasmid was designated as pTΔedaF. The upstream homologous sequence DNA of *eda* was amplified using PCR using *E. coli* MG1655 genomic DNA as a template with the primer pair HomSeq Up eda Fw and HomSeq Up eda Rv. The downstream homologous sequence DNA of *eda* was amplified using PCR using *E. coli* MG1655 genomic DNA as a template, with the primer pair HomSeq Dw eda Fw and HomSeq Dw eda Rv. The donor editing template DNA of *eda* was amplified using overlap extension PCR using the upstream and downstream homologous sequence DNAs of *eda* as a template with the primer pair HomSeq Up eda Fw and HomSeq Dw eda Rv. The amplified donor editing template DNA was cloned between the *EcoR*I and *Hind*III sites of pTΔedaF, and the resulting plasmid was designated as pTΔeda. pTΔppc was constructed by the same procedure as pTΔeda using the primers sgRNA ppc Rv, sgRNA ppc Fw, HomSeqUp ppc Fw, HomSeq Up ppc Rv, HomSeq Dw ppc Fw, and HomSeq Dw ppc Rv. pTΔpck Was constructed by the same procedure as pTΔeda using primers sgRNA pck Rv, sgRNA pck Fw, HomSeqUp pck Fw, HomSeq Up pck Rv, HomSeq Dw pck Fw, and HomSeq Dw pck Rv. pTΔppsA was constructed by the same procedure as pTΔeda using the primers sgRNA ppsA Rv, sgRNA ppsA Fw, HomSeqUp ppsA Fw, HomSeq Up ppsA Rv, and HomSeq Dw ppsA Fw. pTΔxylAB was constructed by the same procedure as pTΔeda using the primers sgRNA xylAB Rv, sgRNA xylAB Fw, HomSeq Up xylAB Fw, HomSeq Up xylAB Rv, HomSeq Dw xylAB Fw, and HomSeq Dw xylAB Rv. pTΔsdaA was constructed by the same procedure as pTΔeda using the primers sgRNA sdaA Rv, sgRNA sdaA Fw, HomSeq Up sdaA Fw, HomSeq Up sdaA Rv, HomSeq Dw sdaA Fw, and HomSeq Dw sdaA Rv.

### Deletion of chromosomal genes

The plasmids used to delete chromosomal genes are listed in Supplementary Data 6. Using the CRISPR-Cas two-plasmid system, each gene in this study was deleted^58^. The plasmid pCas was introduced into the parental strain. An appropriate pTΔ plasmid was additionally introduced to the pCas harboring strain and incubated overnight in the LB medium with 10 g L^−1^ of arabinose as an inducer for λ-Red, without kanamycin and spectinomycin. After recovery culture, the culture medium was seeded to an LB agar plate containing 50 mg L^−1^ of kanamycin and 100 mg L^−1^ of spectinomycin. The target gene deletion was confirmed by colony direct PCR. The plasmids pCas, and pTΔ was eliminated from bacterial the target-gene-deficient strain. All fragments inserted in the plasmids used to inactivate the respective genes were amplified using direct colony PCR using the *E. coli* MG1655 strain as a template and appropriate primers as listed in Supplementary Data 7. To delete *ptsG*, *pheA*, *eda*, *ppc*, *pck*, *ppsA*, *xylAB*, and *sdaA*, the plasmids pTΔptsG, pTΔpheA, pTΔeda, pTΔppc, pTΔpck, pTΔppsA, pTΔxylAB, and pTΔsdaA, respectively, were used.

### Transformation of *E. coli* strains

The transformation of *E. coli* strains was conducted using electroporation with 1350 kV, 600 Ω, and 10-μF electric pulse in a 0.1-cm cuvette using a Gene Pulser (Bio-Rad Laboratories, Hercules, CA, USA). The CFT5 and GX1 strains harboring pZE12-x were designated as GX1xMA and GX1xTYR strains, respectively. The CFT5x strain harboring pSAK-ZYc or pSAK-*tyrA*^fbr^ was designated as CFT5xMA and CFT5xTYR strains, respectively. The GX1x strain harboring pSAK-ZYc or pSAK-*tyrA*^fbr^ was designated GX1xMA and GX1xTYR strains, respectively.

### Sample preparation for ^13^C metabolic analysis

M9 minimal medium containing [U-^13^C]glucose and non-labeled xylose was used for preparing ^13^C metabolic analysis cultures. Each strain was seeded into the medium (supplemented with 0.1-mM isopropyl β-d-1-thiogalactopyranoside) at an initial optical density (OD_600_) of 0.5. The culture medium was sampled when OD_600_ reached 2.0. The sample was centrifuged, and the supernatant was used for *cis*,*cis*-MA analysis. The pellet was washed twice with PBS (−) buffer, suspended in 400 μL of 5-N HCl, and boiled for 1 day at 100 °C. After cooling the HCl-processed liquid to room temperature, 400 μL of 5-N NaOH was added and the solution was centrifuged to collect the supernatant. This processed supernatant was used for cellular constituent amino acid analysis. The dried residues of MA samples were derivatized for 30 min at 37 °C in 50 µL of N-methyl-N-trimethylsilyltrifluoroacetamide and 20 µL of pyridine prior to the analysis. The dried residues of amino acid samples were derivatized for 60 min at 80 °C in 30 µL of N-tert-butyldimethylsilyl-N-methyltrifluoroacetamide with 1% tert-butyldimethylchlorosilane and 30 µL of N,N-dimethylformamide prior to the analysis.

### Analytical methods

Cell growth was determined by measuring OD_600_ using a UVmini-1240 spectrophotometer (Shimadzu Corporation, Kyoto, Japan). The dry cell weight was determined based on OD_600_ using a calibration curve (Supplementary Fig. 13). Glucose was analyzed using a Prominence HPLC System (Shimadzu) equipped with a Shodex SUGAR KS-801 column (grain diameter, 6 µm; L × I.D., 300 × 8.0 mm; Shodex). Water was used as the mobile phase with a flow rate of 0.8 mL min^−1^, and the column was maintained at 50 °C. The HPLC profile was monitored using a refractive index detector.

Glyoxylate was derivatized according to the protocol of Chihara et al.^59^. A 50 μL of culture supernatant was diluted with 950 μL ethanol. To 100 μL of the diluted sample, 50 μL of 100 mmol L^−1^ pyreneboronic acid in ethanol and 50 μL of 200 mmol L^−1^ MBA in ethanol were successively added and the sample was mixed well. After heating at 80 °C for 1 h, 300 μL water was added to the reaction mixture. A 100 μL of the supernatant was injected for HPLC analysis after centrifugation in 20,000×*g* for 10 min. Glyoxylate derivative was analyzed using an HPLC equipped with a 5C_18_-MS-II column (grain diameter, 5 µm; *L* × I.D., 250 × 4.6 mm; Nacalai Tesque). A dual-solvent system was used. Solvent A was 35:65 mixture of acetonitrile and 50 mM acetate buffer (pH 4.0), and solvent B was acetonitrile. The flow rate of the mobile phase was 1.0 mL min^−1^, and the column was maintained at 30 °C. Gradient was initiated as a 100:0 mixture of A and B (0–15 min), shifted to a 0:100 mixture of A and B (15–15.5 min), and maintained a 0:100 mixture of A and B (15.5–26 min). The HPLC profile was monitored using a UV–VIS detector at 450 nm.

MA, acetate, lactate, and xylonate were analyzed using an HPLC equipped with a SCR-102H column (grain diameter, 7 µm; *L* × I.D., 300 × 8 mm; Shimadzu). *p*-Toluenesulfonic acid (5 mM) was used as the mobile phase with a flow rate of 2.0 mL min^−1^, and the column was maintained at 40 °C. The HPLC profile was monitored using a conductivity detector.

PCA, CA, l-phenylalanine, l-tyrosine, tryptophan, *p*-aminobenzoate, and *p*-hydroxybenzoate were analyzed using an HPLC equipped with a PBr column (grain diameter, 5 µm; *L* × I.D., 250 × 4.6 mm; Nacalai Tesque). A dual-solvent system was used. Solvent A was 0.2% phosphate buffer, and solvent B was methanol. The flow rate of the mobile phase was 1.0 mL min^−1^, and the column was maintained at 40 °C. Gradient was initiated as an 80:20 mixture of A and B (0–15 min), shifted to a 50:50 mixture of A and B (15–20 min), and subsequently shifted back to an 80:20 mixture of A and B (20–25 min). The HPLC profile was monitored using a UV–VIS detector at 240 nm.

GC–MS was performed using a GC–MS-QP2010 Ultra instrument (Shimadzu) equipped with a CP-Sil 8 CB-MS capillary column (film thickness, 0.25 μm; *L* × I.D., 30 m × 0.25 mm; Agilent). Helium was used as the carrier gas to maintain a flow rate of 2.1 mL min^−1^. The injection volume was 1 μL with a split ratio of 1:10. The produced MA was analyzed as follows. The oven temperature was initially held at 150 °C for 1 min, raised to 240 °C at 15 °C min^−1^, further raised to 300 °C at 120 °C min^−1^, and finally held at 300 °C for 3 min The total run time was 10 min The amino acids were analyzed as follows. The oven temperature was initially held at 150 °C for 5 min, raised to 300 °C at 10 °C min^−1^, and finally held at 300 °C for 5 min The total run time was 25 min The other settings were as follows: interface temperature, 300 °C; ion-source temperature, 150 °C, and electron ionization impact, 70 eV.

### Metabolome analysis

According to the previous report^60^, metabolome analysis was performed with some modifications. Briefly, cells were cultured in the M9Y medium supplemented with 10 g L^−1^ of glucose and 10 g L^−1^ of xylose until the midterm at the logarithmic growth phase (ATCC31882x, 5.5 h; CFT5x, 14 h; GX1x, 14 h). 2 mL of each culture broth was harvested by rapid filtration. After the filtrated cells were dropped into cold methanol solution quickly to quench metabolic flow, the intracellular metabolites were extracted in solvent mixture (CHCl_3_:CH_3_OH:H_2_O, 2.5:2.5:1, v/v/v). After centrifugation at 15,000 × *g* at 4 °C for 15 min, 300 μL of the upper phase was collected. The metabolites were quantified by high-performance liquid chromatography coupled with electrospray ionization tandem mass spectrometry combined with the method package to measure primary metabolites (LC–MS-8040 triple quadrupole LC/MS/MS spectrometer; Shimadzu, Kyoto, Japan).

### Quantification of transcriptional level

The transcriptional expression of *maeB, ppsA*, *ppc*, *pckA*, *pgi*, *zwf*, *gltA*, and *arcA* in each strain was quantified using real-time PCR. Total RNA was isolated from individual cultures using a NucleoSpin RNA column (Takara Bio, Shiga, Japan) according to the manufacturer's protocol. Reverse transcription reactions and quantitative real-time PCR was performed using an Mx3005P Real-Time QPCR System (Agilent Technologies, Santa Clara, CA, USA) with RNA-direct SYBR Green Realtime PCR Master Mix (TOYOBO, Osaka, Japan). The primer pairs are listed in Supplementary Data 7. The normalized transcriptional level of each mRNA was calculated by the relative quantification method using the *mdoG* gene (coding glucan biosynthesis protein G) as the housekeeping gene^61^.

### Reporting summary

Further information on research design is available in the Nature Research Reporting Summary linked to this article.

## Supplementary information

Supplementary Information

Peer Review

Reporting Summary

Description of Additional Supplementary Files

Supplementary Data 1

Supplementary Data 2

Supplementary Data 3

Supplementary Data 4

Supplementary Data 5

Supplementary Data 6

Supplementary Data 7

## Data Availability

A reporting summary for this Article is available as a Supplementary Information file. The datasets generated and analyzed during the current study are available from the corresponding author upon request. The data that support the findings of this study are available from the corresponding author upon reasonable request. The source data underlying Figs. 2b, 2c, 4b, and 5–8, as well as Supplementary Figs. 2a, b, d, 3–6, 8–15, and 17 are provided as a Source Data file.

## Source data

Source Data
