# Publisher Correction: Metabolic engineering of *Escherichia coli* for shikimate pathway derivative production from glucose-xylose co-substrate

**DOI:** 10.1038/s41467-020-14710-5

**Published:** 2020-02-20

**Authors:** Ryosuke Fujiwara, Shuhei Noda, Tsutomu Tanaka, Akihiko Kondo

**Affiliations:** 1 Department of Chemical Science and Engineering, Graduate School of Engineering, Kobe University, 1-1 Rokkodai, Nada, Kobe 657-8501 Japan; 2 Center for Sustainable Resource Science, RIKEN, 1-7-22 Suehiro-cho, Tsurumi-ku, Yokohama, Kanagawa 230-0045 Japan; 3 Graduate School of Science, Technology and Innovation, Kobe University, 1-1 Rokkodai, Nada, Kobe 657-8501 Japan

**Keywords:** Metabolic engineering, Applied microbiology

Correction to: *Nature Communications* 10.1038/s41467-019-14024-1, published online 14 January 2020.

In the original version of this Article, Fig. 3 was incorrectly duplicated as Fig. 7. Also, the legend to Fig. 7 panel ‘e’ should read ‘Production yield of *cis,cis*-muconic acid (MA) from glucose (g of produced MA g^−1^ of consumed glucose). Red, orange, yellow, purple, and blue symbols indicate the results of cultivation in 0%, 25%, 50%, 75%, and 100% glucose medium, respectively, in ‘**a**–**e**’; ‘**a**–**h**’ were referred to in the original version. This has been corrected in both the PDF and HTML versions of the Article.

