# Supplementary material for: Metabolic engineering of Escherichia coli for shikimate pathway derivative production from glucose–xylose co-substrate
Source: Nat Commun. 2020 Jan 14;11:279. doi: 10.1038/s41467-019-14024-1 (PMC6959354; doi:10.1038/s41467-019-14024-1)
Supplement: Supplementary file 1 — Supplementary Information [file 41467_2019_14024_MOESM1_ESM.pdf]

Metabolic engineering of *Escherichia coli* for shikimate pathway derivative production from glucose–xylose co-substrate

Fujiwara *et al.*

Supplementary Discussion 1. Characteristics of central metabolic genes expression in CFT5 strain

In the M9M medium culture of CFT5, lag phase was very long, about two days (Supplementary Figure 2b). It is thought that there are differences in expression of genes in central metabolism from ATCC31882, the parental strain which can grow rapidly in M9 minimal medium. To examine the characteristics of gene expression in CFT5, we measured genes transcription by real time revers transcription PCR. ATCC31882 and CFT5 were cultured in M9 minimal medium containing 20 g/L glucose supplemented with 10 mM sodium malate. The initial optical density at 600 nm (OD_{600}) of the cultures was 1.0, and cells were collected 1h (ATCC31882, lag phase), or 22h (CFT5, lag phase) and 66h (CFT5, early logarithmic growth phase) for real time RT-PCR analysis. An increase or decrease compared with ATCC31882 was measured in seven genes, *maeB* (coding malic acid enzyme), *ppsA* (coding PEP synthase), *ppc* (coding PEP carboxylase), *pckA* (coding PEP carboxykinase), *pgii* (coding G6P isomerase), *zwf* (coding G6P dehydrogenase), *gltA* (coding citrate synthase) (Supplementary Figure 2c). Supplementary Figure 2d shows the results of relative expression levels of mRNA in each gene. In the lag phase, CFT5 increased the expression of *maeB*, *ppsA*, *pckA*. These three genes code enzymes of gluconeogenesis which is the reverse metabolism of glucose catabolism. Expression of *gltA* was decreased in the lag phase of CFT5. Since citrate synthase catalyzes the first reaction of the TCA cycle, it is thought that the TCA cycle would have been suppressed in CFT5. Furthermore, expression of *ppc* was also decreased. PEP carboxylase is the enzymes of anaplerosis which is the metabolic reaction supplying oxaloacetate, an intermediate of the TCA cycle. These results suggested that the carbon flow to the TCA cycle reduced. The expression of *pgi* and *zwf* which code the

enzymes catalyzing the first reaction of glycolysis and pentose phosphate pathway, respectively, were enhanced in the lag phase of CFT5. In the early logarithmic growth phase of CFT5, all genes were increased when compared with the lag phase of CFT5, but there was no change in the increase / decrease trend when compared with ATCC31882. Taken together, it is thought that sugar catabolism was promoted in the upstream of the central metabolism, gluconeogenesis was enhanced and the TCA cycle was suppressed, which might have caused the long lag phase and the slow cell growth in CFT5.

Supplementary Discussion 2. Metabolome analysis of PMPE strains

To analyze the changes in the accumulation of metabolites by PMPE, three strain ATCC31882x, CFT5x, and GX1x were cultured in M9Y medium containing glucose and xylose. After culturing, the levels of metabolites, glucose 6-phosphate (G6P), fructose 6-phosphate (F6P), fructose 1,6-biphosphate (F16BP), 3-phosphoglycerate (3PG), 2-phosphoglycerate (2PG), phosphoenolpyruvate (PEP), pyruvate, 6-phosphogluconate (6PG), ribulose 5-phosphate (Ru5P), ribose 5-phosphate (Ro5P), Sedoheptulose 7-phosphate (S7P), and Erythrose 4-phosphate (E4P) were analyzed via LC-MS. The initial optical density at 600 nm (OD_{600}) of the cultures was 0.05, and cells were harvested 5.5h (ATCC31882x) or 14h (CFT5x and GX1x) for LC-MS analysis. Supplementary Figure 14 shows the relative amount of metabolites the specific accumulated amount of metabolites per OD_{600}, used that in ATCC31882x as a benchmark. In GX1x, the accumulated PEP increased 28.6-fold compared to ATCC31882x, and 5.2-fold compared to CFT5x. CFT5x accumulated PEP higher than ATCC31882x, but also increased the accumulated pyruvate. On the other hand, the accumulated pyruvate in GX1x was at the same level as ATCC31882x.

Although there was no significant difference in the total amount of 3PG and 2PG between ATCC31882x and CFT5x, that of GX1x was increased 2.0-fold compared to ATCC31882x. The accumulated F16BP in CFT5x and GX1x were significantly lower than ATCC31882. It would have been caused by the accumulation of PEP because PEP inhibits 6-phosphofructokinase 1 (coded by *pfkA*) which synthesize F16BP from F6P and activates Fructose 1,6-bisphosphatase (coded by *fbp*) which synthesize F6P from F16BP [1,2]. Therefore, PEP accumulation degrades the flux of glycolysis and enhances the flux of pentose phosphate pathway (PPP)[3]. In GX1x, four PPP metabolites, 6PG, Ru5P, Ro5P, and S7P, were significantly increased than ATCC31882x. We also investigated the levels of ATP, ADP, AMP, NADH, NAD^+, NADPH, and $NADP^+$. In GX1x, the ATP level was significantly reduced compared to ATCC31882x and CFT5 (Supplementary Figure 15a). In CFT5 and GX1, GalP/Glk system consumes 1 mole of ATP when converting 1 mole of glucose to G6P. Furthermore, pyruvate kinases (coded by *pykA* and *pykF*) which synthesize pyruvate and ATP from PEP were disrupted in CFT5 and GX1. These metabolic modifications would have caused the lower levels of ATP and the late and lower cell growth (Fig. 2b, 4b, 5a). The NADH level in GX1x was significantly reduced compared to ATCC31882x, and NADH was not detected in CFT5x (Supplementary Figure 15b). These results are consistent with the transcriptome analysis suggesting the carbon flux suppression of the TCA cycle in CFT5 (Supplementary Fig. 2d) and show that ATP synthesis by oxidative phosphorylation did not work well due to the restriction of the NADH regeneration in the TCA cycle. Additionally, the NADPH in CFT5x and GX1x were not detected, which caused by the enhanced shikimate pathway because shikimate dehydrogenase consumes 1 mole of NADPH when synthesis 1 mole of shikimate from 3-dehydroshikimate.

Supplementary Discussion 3. 1,2-Propanediol production in the PMPE strain

To confirm the versatility of PMPE in non-shikimate pathway, we attempted to produce 1,2-propanediol using the PMPE strain (Supplementary Figure 16). The GX1xPD strain, a GX1-derived strain harboring *Bs_mgsA*, *gldA*, and *fucO*, encoding methylglyoxal synthase, glycerol dehydrogenase, and 1,2-propanediol oxidoreductase, respectively, was cultivated in M9Y medium containing both 20 g/L of glucose and 5 g/L of xylose in micro-aerobic condition at 30°C. GX1xPD strain produced 0.53 ± 0.01 g/L of 1,2-propanediol after 96 h of cultivation, representing a 1.40-fold increase compared with that produced by the control strain CTR2PD (Supplementary Figure 17). The maximum productivity of 1,2-propanediol in CTR2PD and GX1xPD were 0.010 and 0.015 g/L/h, respectively. The production yield reached 0.53 ± 0.16 g/g-glucose after 96 h of cultivation, reflecting a 11.4-fold increase compared with that using the CTR2PD strain and corresponding to 62% of the theoretical maximum without considering cell growth (0.84 g/g of glucose)[4]. Jain et al. engineered the metabolism in *E. coli* for production of 1,2-propanediol. They disrupted *tpiA* which is coding triosephosphate isomerase to increase the production[5]. The final strain in this study produced 1.2 g/L of 1,2-propanediol from 20 g/L of glucose. However, *tpiA*-deficient strains can only produce up to 1 mole of 1,2-propanediol from 1 mole of glucose, thus the theoretical maximum yield is 0.42 g/g. In GX1xPD, the yield was higher than the theoretical maximum yield in *tpiA*-deficient strains, it suggests that the PMPE strategy has the potential to dramatically improve the yield of 1,2-propanediol from glucose. For further improvement of 1,2-propanediol production, the optimization of metabolism is necessary because GX1 was engineered to increase the production of shikimate pathway derivatives. In particular, improving NADH

regeneration is necessary for 1,2-propanediol production in the PMPE strain. 2 moles of NADH was required when 1 mole of 1,2-propanediol is synthesized (Supplementary Figure 16). The NADH level in the PMPE strain was very low (Supplementary Figure 15b). By improving the redox levels of NADH / NAD^+, further increase in the production of 1,2-propanediol can be expected.

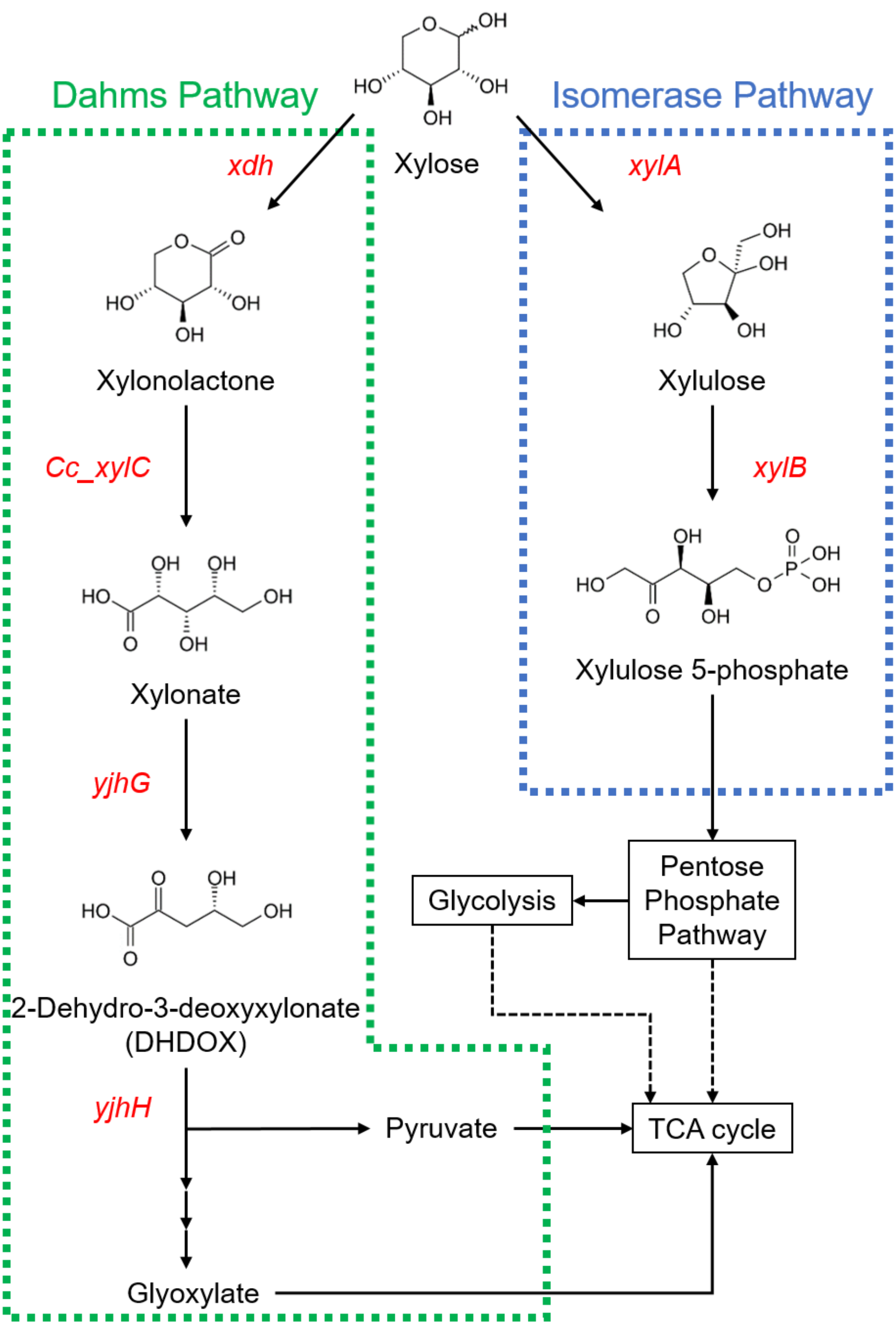

Supplementary Figure 1. Diagram of the xylose catabolite pathway. Green and blue framed pathways are the Dahms and isomerase pathways, respectively.

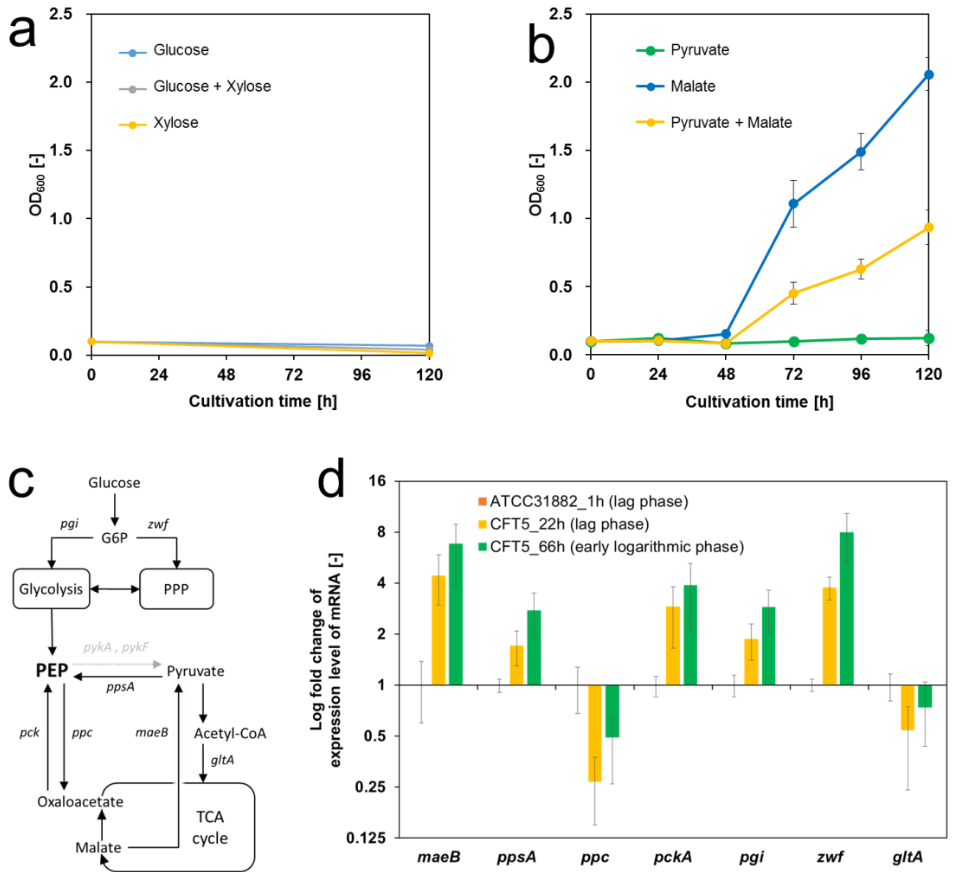

Supplementary Figure 2. Growth of the CFT5 strain. (a) Blue, gray, and yellow symbols indicate M9 minimal medium supplemented with glucose, a glucose–xylose mixture, and xylose, respectively. (b) Green, blue, and yellow symbols indicate the use of minimal medium containing glucose supplemented with 10-mM sodium pyruvate, 10-mM sodium malate, and mixture of 5-mM pyruvate and 5-mM malate, respectively. (c) Diagram of central metabolism in CFT5. (d) Transcriptome analysis of CFT5. Yellow and green bars indicate log fold change of expression level of mRNA from ATCC31882 (after 1h cultivation) in the lag phase of CFT5 (after 22h cultivation) and in the early logarithmic phase of CFT5 (after 66h cultivation), respectively. The data are presented as the average of three independent experiments, and error bars indicate standard errors. Source data underlying Supplementary Figure 2a, 2b, and 2d are provided as a Source Data file.

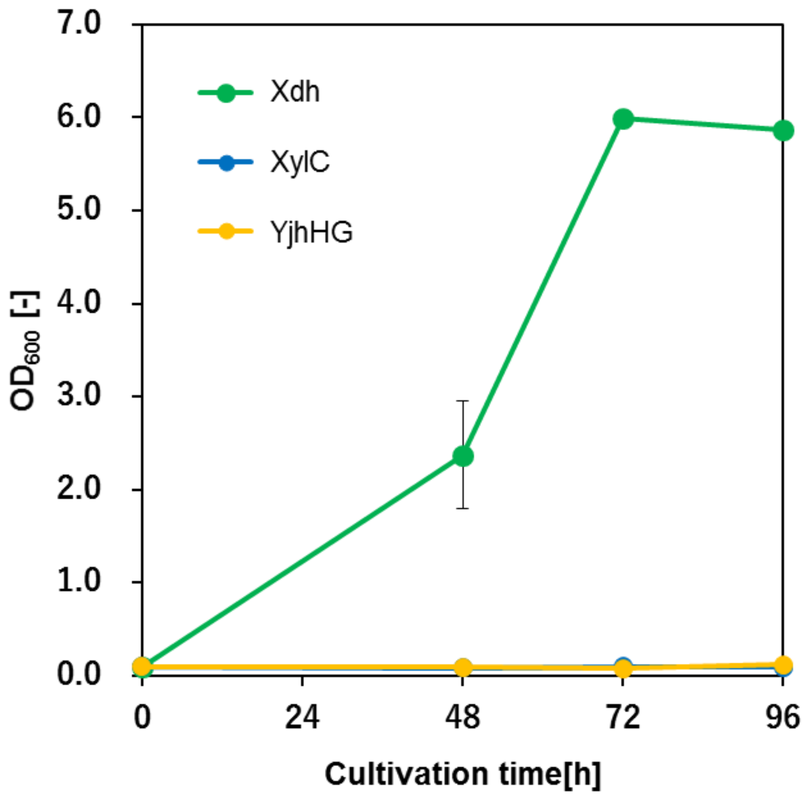

Supplementary Figure 3. Growth of the CFT5 derived strain expressing each Dahms pathway enzyme in M9 minimal medium containing glucose as a sole carbon source. Green, blue, and yellow symbols indicate the bacterial cell growth of CFT5 harboring pZE12-*xdh*, CFT5 harboring pZE12-*xylC*, and CFT5 harboring pZE12-*yjhHG*, respectively. Source data are provided as a Source Data file.

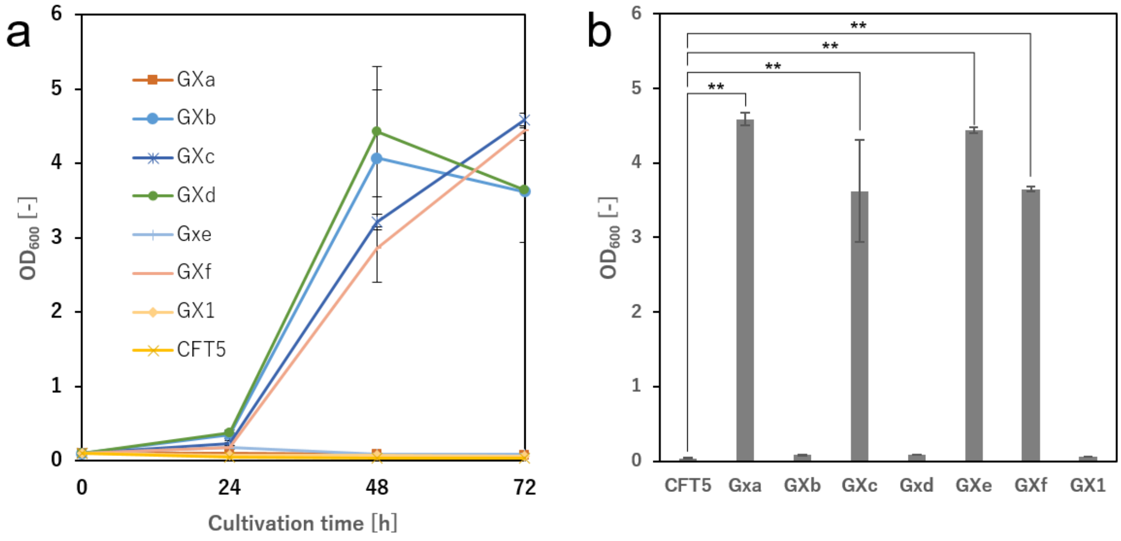

Supplementary Figure 4. Bacterial cell growth in M9 minimal medium with glucose as the sole carbon source. (A) Time courses of cell growth. (B) Cell density after 72 h of cultivation. The data are presented as the average of three independent experiments, and error bars indicate standard errors. P values are computed using the two-tailed Student's *t*-test (***P* < 0.01). Source data are provided as a Source Data file.

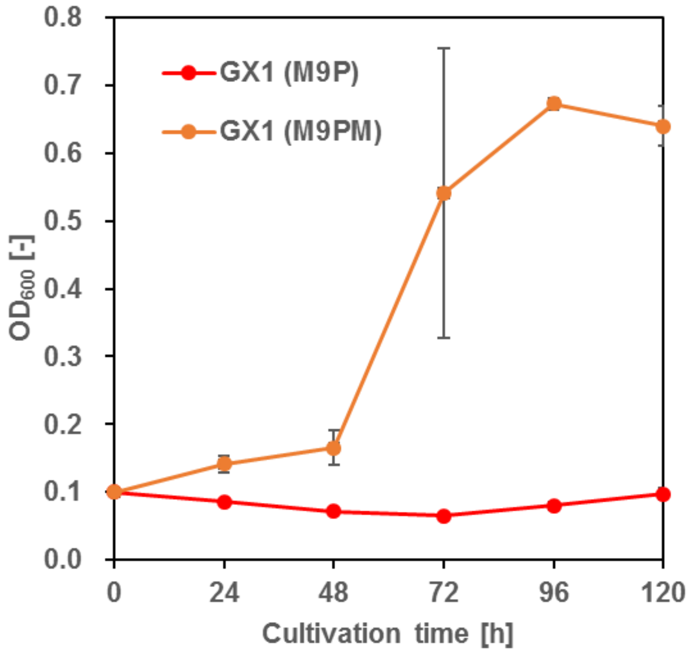

Supplementary Figure 5. The growth of the GX1 strain. Red and orange symbols indicate M9 minimal medium supplemented with pyruvate (M9P) medium and M9P minimal medium supplemented with 10-mM malate (M9PM), respectively. The data are presented as the average of three independent experiments, and error bars indicate standard errors. Source data are provided as a Source Data file.

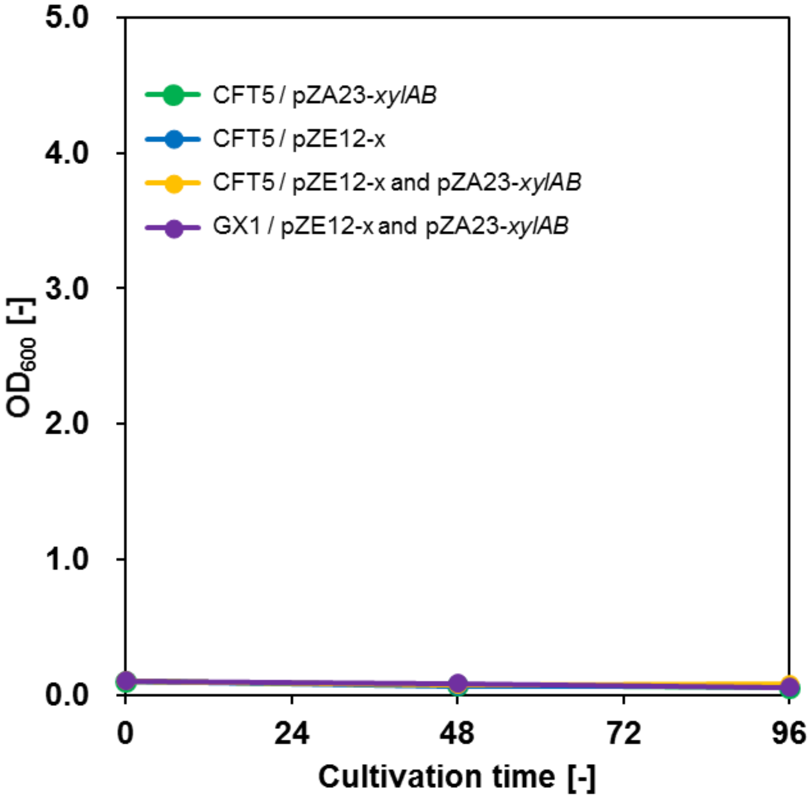

Supplementary Figure 6. The effect of overexpressing XylA and XylB on the cell growth in M9 minimal medium with xylose as a sole carbon source. Green, blue, and yellow symbols indicate the bacterial cell growth of CFT5 harboring pZA23-*xylAB*, CFT5 harboring pZE12-x, and CFT5 harboring pZE12-x and pZA23-*xylAB*. Purple symbols indicate the bacterial cell growth of GX1 harboring pZE12-x and pZA23-*xylAB*. Source data are provided as a Source Data file.

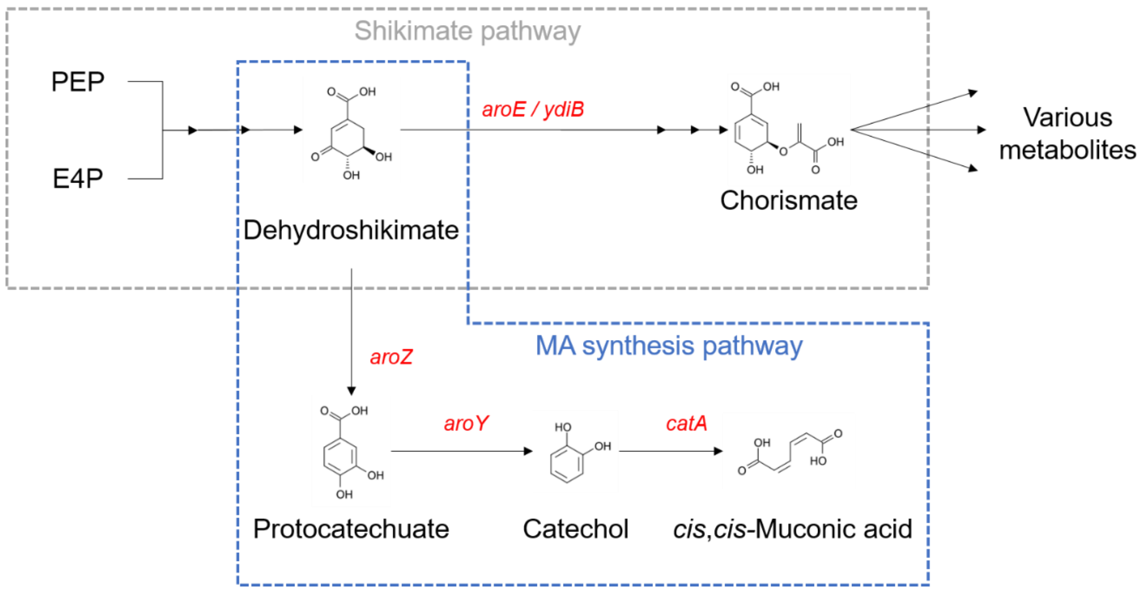

Supplementary Figure 7. Diagram of the shikimate and *cis,cis*-muconic acid synthesis pathways. Gray and blue framed pathways are the shikimate and MA synthesis pathways, respectively.

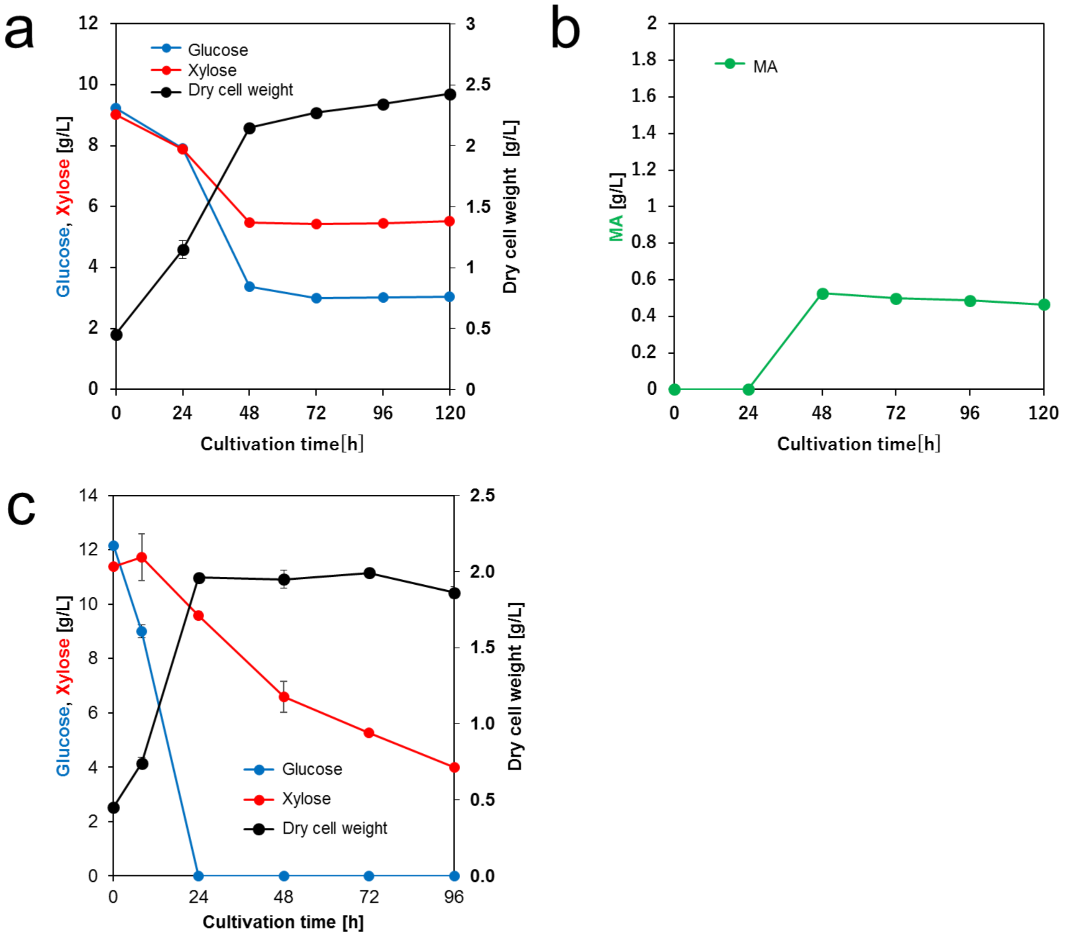

Supplementary Figure 8. Culture profiles of CTR2MA and ATCC31882 strains in M9 minimal medium. (a) Culture profiles of CTR2MA. Black, blue, and red symbols indicate bacterial cell growth, glucose consumption, and xylose consumption, respectively. (b) MA production in CTR2MA. (c) Culture profiles of ATCC31882. Black, blue, and red symbols indicate bacterial cell growth, glucose consumption, and xylose consumption, respectively. The data are presented as the average of three independent experiments, and error bars indicate standard errors. Source data are provided as a Source Data file.

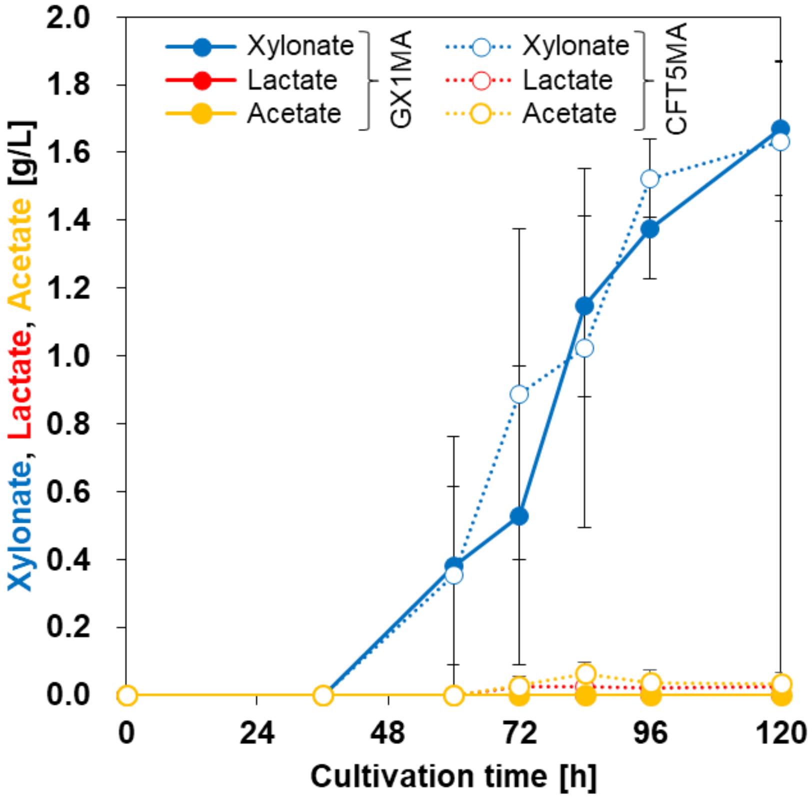

Supplementary Figure 9. Organic acid accumulation of *cis,cis*-muconic acid (MA)-producing strains in M9 minimal medium. Black, red, and yellow symbols indicate xylonate, lactate, and acetate concentration, respectively. All open and filled symbols indicate the results for the CFT5xMA and GX1xMA strains, respectively. (b) Mass balance of GX1xMA and CFT5xMA. Orange, yellow, and gray bars indicate the proportion of produced MA, xylonate, and other components including biomass and exhausted CO_2, respectively. Blue and red bars indicate the proportion of consumed glucose and xylose, respectively. The data are presented as the average of three independent experiments, and error bars indicate standard errors. Source data are provided as a Source Data file.

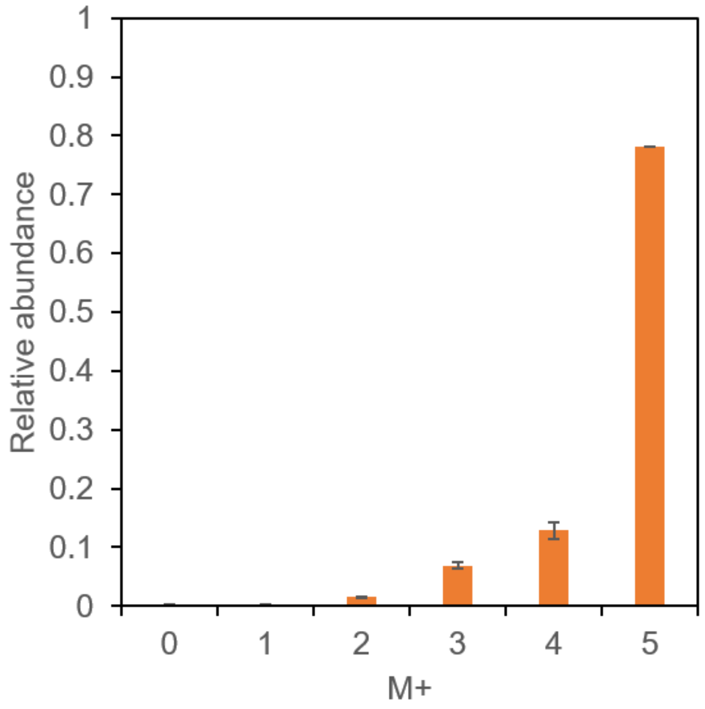

Supplementary Figure 10. ^{13}C-metabolic analysis of produced *cis*,*cis*-muconic acid (MA) in the flask fermentation of the GX1xMA strain. Orange bars indicate the mass isotopomer distributions of MA from tracer experiments with [U-^{13}C]glucose and non-labeled xylose in the GX1xMA strain. The vertical axis presents relative abundance. The horizontal axis is M+, which denotes the difference with a fully unlabeled isotopomer, in m/z of a mass fragment (fully unlabeled isotopomer, M+ = 0). The maximum value of M+ is the number of constituent carbons of MA in the mass fragment. The data are presented as the average of three independent experiments, and error bars indicate standard errors. Source data are provided as a Source Data file.

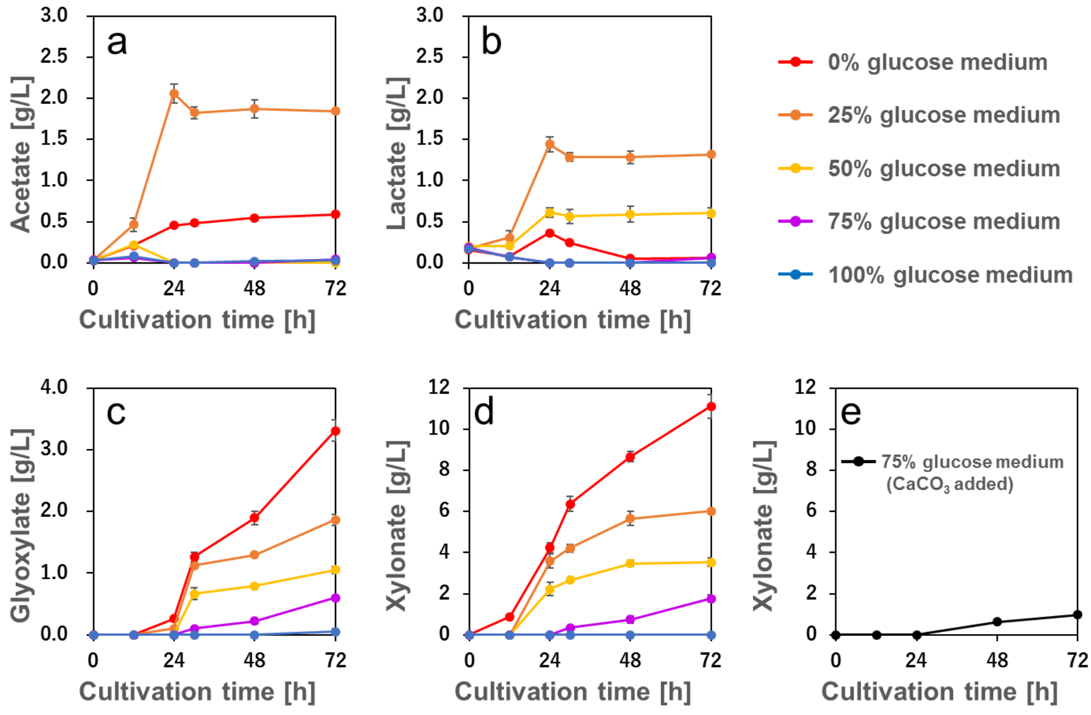

Supplementary Figure 11. Culture profiles of the GX1xMA strain in M9 minimal medium containing 20-g/L sugar(s) and 5-g/L yeast extract. (**a**) Acetate accumulation. (**b**) Lactate accumulation. (**c**) Glyoxylate accumulation. (**d**) Xylonate accumulation. (**e**) Xylonate accumulation in 75% glucose medium with $CaCO_3$ addition condition. Red, orange, yellow, purple, and blue symbols indicate the results of cultivation in 0%, 25%, 50%, 75%, and 100% glucose medium, respectively, in a–d. The data are presented as the average of three independent experiments, and error bars indicate standard errors. Source data are provided as a Source Data file.

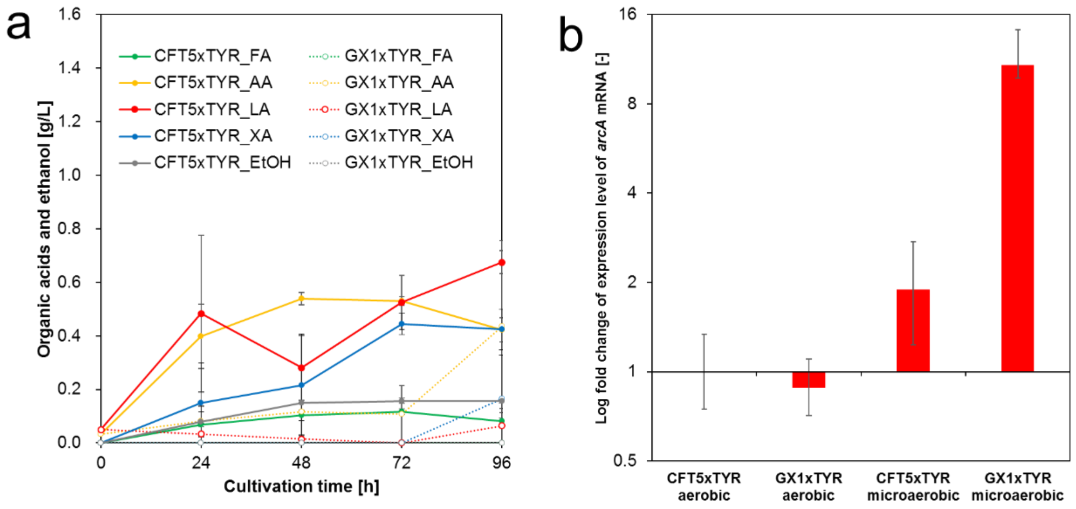

Supplementary Figure 12. Culture profile of CFT5xTYR and GX1xTYR. (A) By-products production in anaerobic condition. All filled symbols and all open symbols indicate results of CFT5xTYR and GX1xTYR, respectively. FA, formate; AA, acetate; LA, lactate; XA, xylonate; and EtOH, ethanol. (B) Relative transcriptional levels of *arcA*. The *arcA* mRNA level of CFT5xTYR in aerobic condition was used as a benchmark. The data are presented as the average of three independent experiments, and error bars indicate standard errors. Source data are provided as a Source Data file.

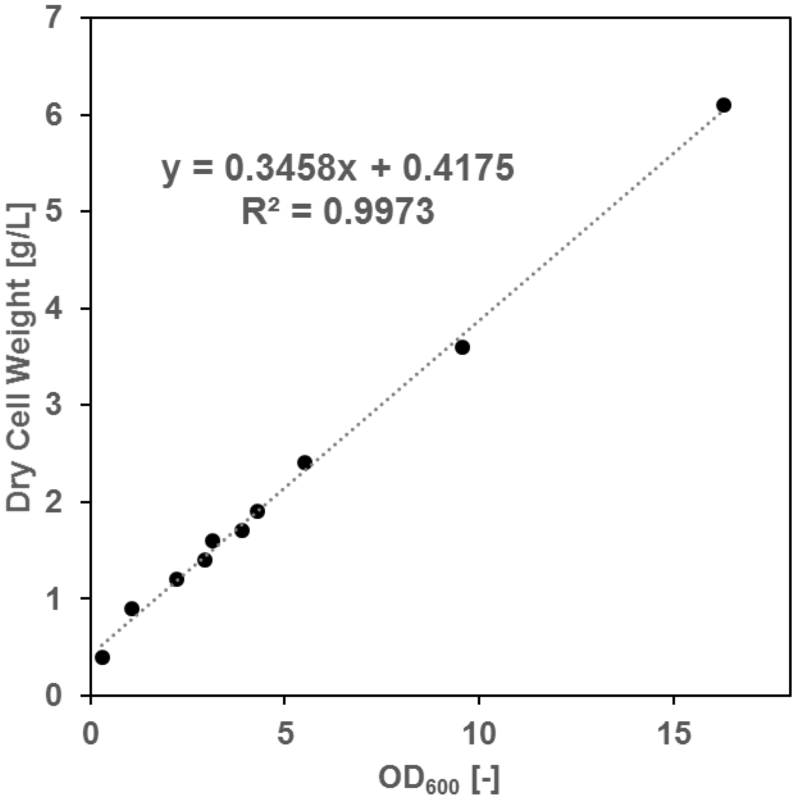

Supplementary Figure 13. Calibration curve for obtaining DCW from OD. Source data are provided as a Source Data file.

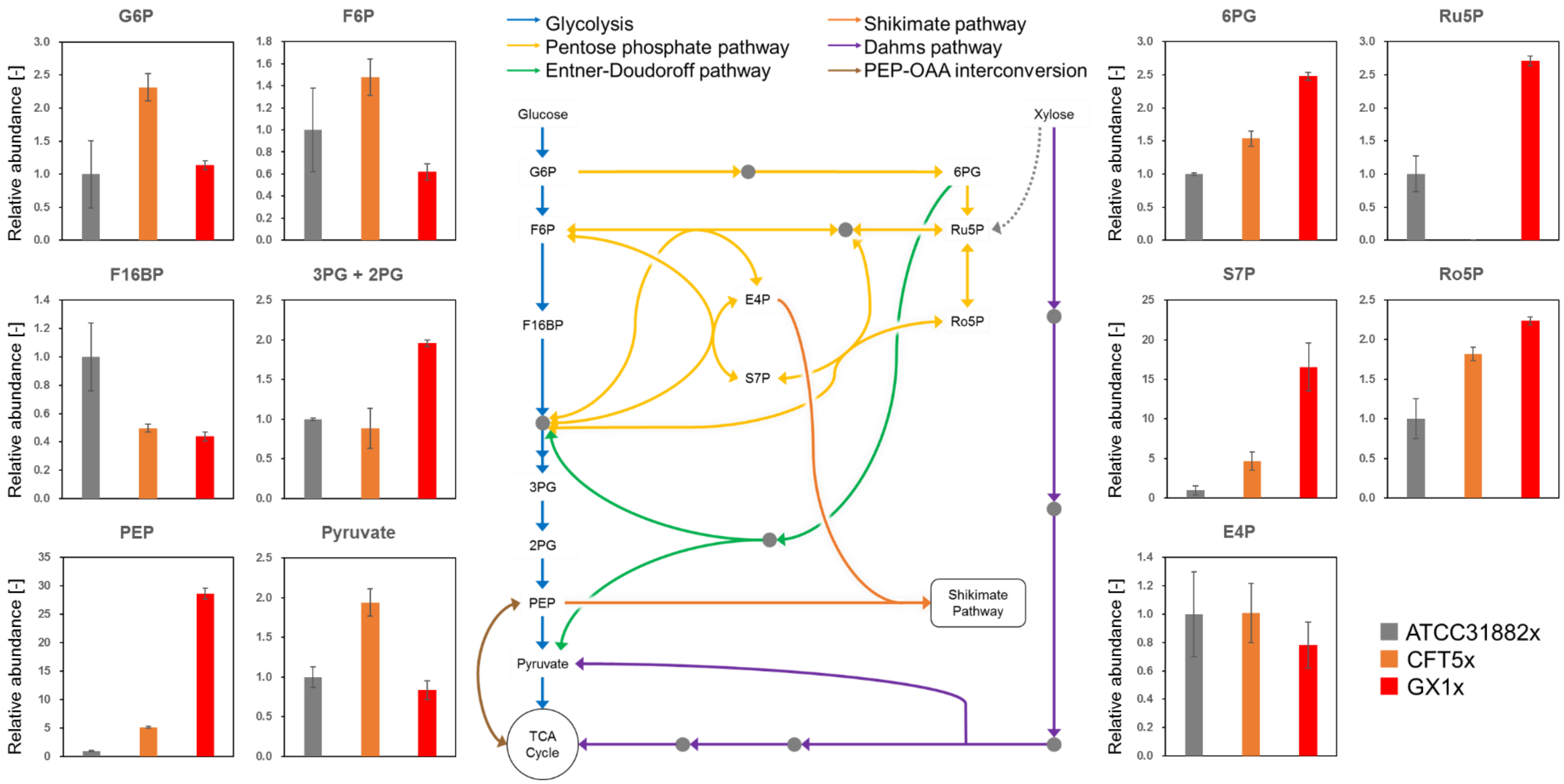

Supplementary Figure 14. Metabolome analysis of PMPE strains. Figures indicate the specific accumulated amount of metabolites per OD_{600}, used that in ATCC31882x as a benchmark. In 3PG and 2PG, these were evaluated as a combined value. Gray, orange, and red bars indicate the results of ATCC31882, CFT5x, and GX1, respectively. The data are presented as the average of three independent experiments, and error bars indicate standard deviations. Source data are provided as a Source Data file.

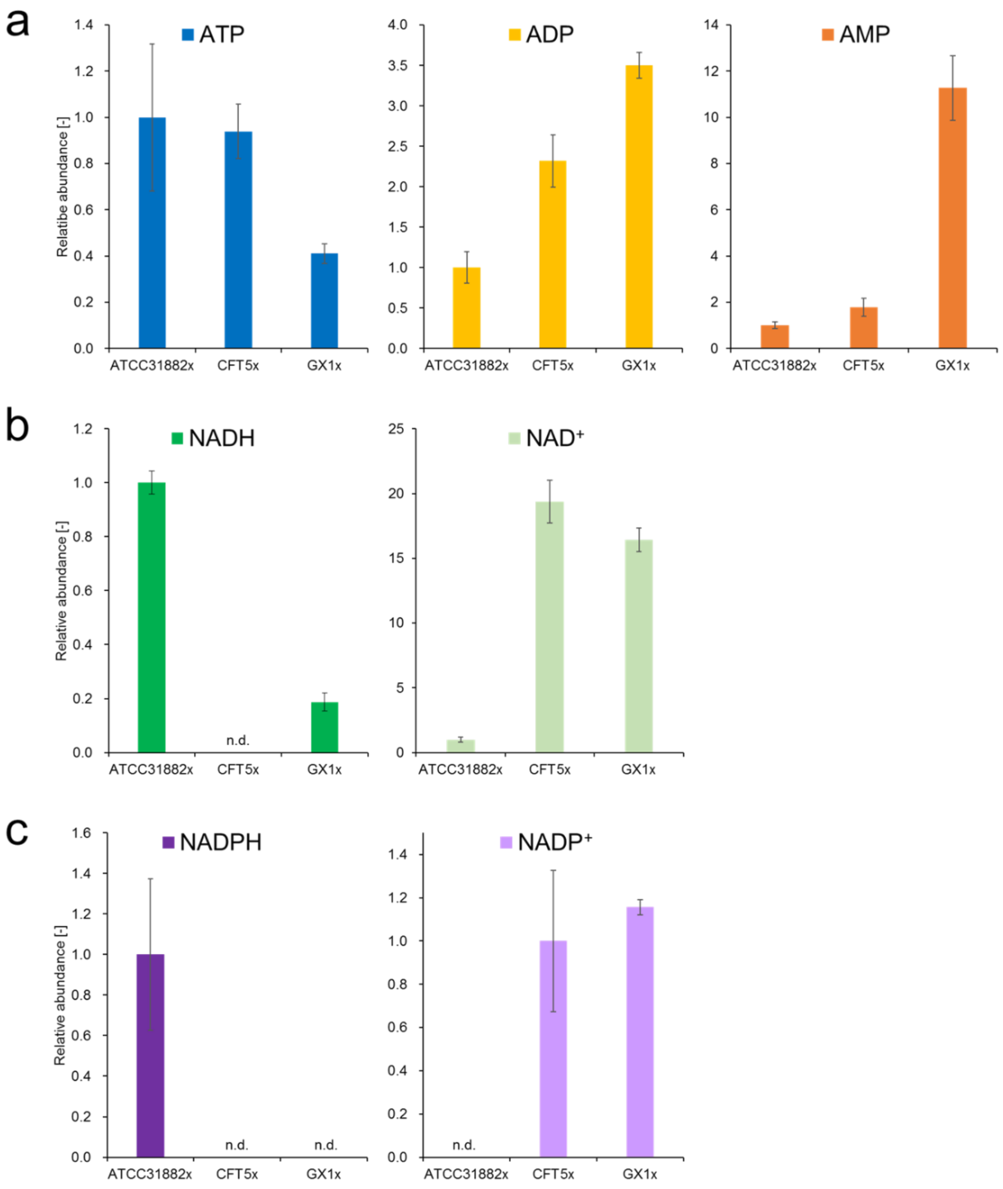

Supplementary Figure 15. The relative abundance of ATP, ADP, AMP, NADH, NAD^+, NADPH, and $NADP^+$ in PMPE strains. (a) Blue, yellow, and orange bars indicate ATP, ADP, and AMP, respectively. (b) Dark green, and light green bars indicate NADH, and NAD^+, respectively. (c) Dark purple, and light purple bars indicate NADPH, and $NADP^+$, respectively. Figures indicate the specific accumulated amount of metabolites per OD_{600}. CFT5x was used as a benchmark in $NADP^+$ and ATCC31882x was used as a benchmark in other metabolites. n.d., not detected. The data are presented as the average of three independent experiments and error bars indicate standard deviations. Source data are provided as a Source Data file.

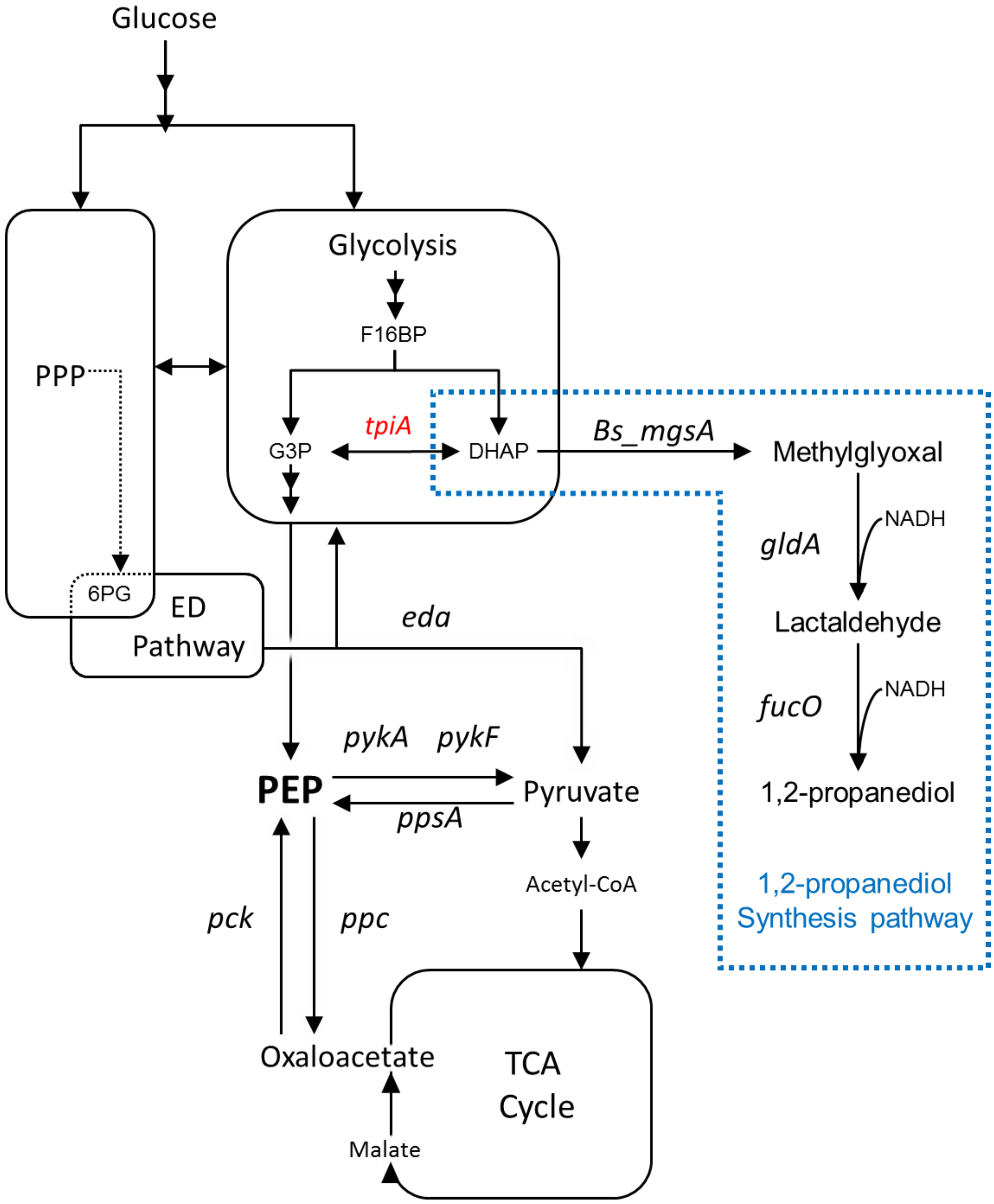

Supplementary Figure 16. Diagram of metabolic pathway incruding 1,2-propanediol synthesis pathay. ED Pathway, Entner–Doudoroff Pathway; PEP, phosphoenolpyruvate; F16BP, fructose 1,6-biphosphate; G3P, Glyceraldehyde 3-phosphate; DHAP, Dihydroxyacetone phosphate; and 6PG, 6-phosphogluconate.

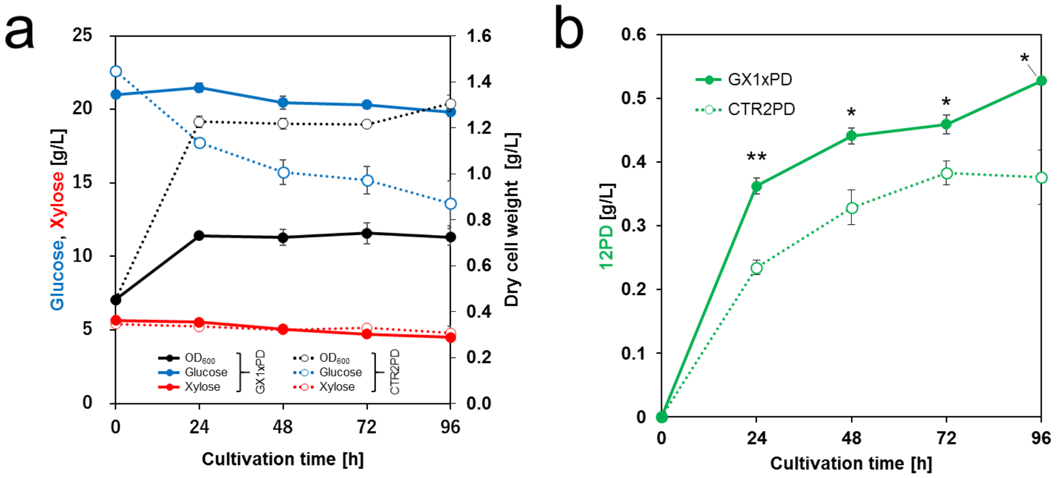

Supplementary Figure 17. Culture profiles of 1,2-propanediol-producing strains in M9 minimal medium. (**a**) Black, blue, and red symbols indicate bacterial cell growth, glucose consumption, and xylose consumption, respectively. (**b**) Green symbols indicate the produced amounts of 1,2-propanediol. All open and filled symbols indicate the results for the CTR2PD and GX1xPD strains, respectively. The data are presented as the average of three independent experiments, and error bars indicate standard errors. P values were computed using the two-tailed Student's *t*-test (*, $P < 0.05$; **$P < 0.01$). Source data are provided as a Source Data file.

Supplementary Table 1. The specific rates of cell growth, substrate consumption, and metabolite production of GX1xMA in each glucose:xylose ratio

Glucose content	specific growth rate [h^{-1}]	specific glucose consumption rate [h^{-1}]	specific xylose consumption rate [h^{-1}]	specific acetate consumption rate [h^{-1}]	specific lactate consumption rate [h^{-1}]
0%	0.067	-	0.026	0.151	0.242
25%	0.108	0.082	0.096	0.127	0.138
50%	0.126	0.081	0.136	-	0.092
75%	0.136	0.046	0.053	-	-
100%	0.119	0.004	-	-	-

Supplementary references

1. Kotlarz, D. & Buc, H. Phosphofructokinases from *Escherichia coli*. *Methods Enzymol.* **90**, 60–70 (1982).
2. Hines, J. K., Fromm, H. J. & Honzatko, R. B. Novel allosteric activation site in *Escherichia coli* fructose-1,6-bisphosphatase. *J. Biol. Chem.* **281**, 18386–93 (2006).
3. Meza, E., Becker, J., Bolivar, F., Gosset, G. & Wittmann, C. Consequences of phosphoenolpyruvate:sugar phosphotranferase system and pyruvate kinase isozymes inactivation in central carbon metabolism flux distribution in *Escherichia coli*. *Microb. Cell Fact.* **11**, 127 (2012).
4. Cameron, D. C., Altaras, N. E., Hoffman, M. L. & Shaw, A. J. Metabolic Engineering of propanediol pathways. *Biotechnol. Prog.* **14**, 116–125 (1998).
5. Jain, R., Huang, J., Yuan, Q. & Yan, Y. Engineering microaerobic metabolism of *E. coli* for 1,2-propanediol production. *J. Ind. Microbiol. Biotechnol.* **42**, 1049–1055 (2015).
