# Supplementary material for: Metabolic engineering of Escherichia coli for shikimate pathway derivative production from glucose–xylose co-substrate
Source: Nat Commun. 2020 Jan 14;11:279. doi: 10.1038/s41467-019-14024-1 (PMC6959354; doi:10.1038/s41467-019-14024-1)
Supplement: Supplementary file 4 — Description of Additional Supplementary Files [file 41467_2019_14024_MOESM4_ESM.docx]

**Description of Additional Supplementary Files**

File name: Supplementary Data 1
Description: Sequence of xdh from C. crescentus

File name: Supplementary Data 2
Description: Sequence of xylC from C. crescentus

File name: Supplementary Data 3
Description: Sequence of yjhH from E. coli

File name: Supplementary Data 4
Description: Sequence of yjhG from E. coli

File name: Supplementary Data 5
Description: Sequence of Bs_mgsA from B. subtilis

File name: Supplementary Data 6
Description: List of strains and plasmids

File name: Supplementary Data 7
Description: List of primers
