# Supplementary material for: Metabolic engineering of Escherichia coli for shikimate pathway derivative production from glucose–xylose co-substrate
Source: Nat Commun. 2020 Jan 14;11:279. doi: 10.1038/s41467-019-14024-1 (PMC6959354; doi:10.1038/s41467-019-14024-1)
Supplement: Supplementary file 2 — Peer Review [file 41467_2019_14024_MOESM2_ESM.pdf]

Reviewers' comments:

Reviewer #1 (Remarks to the Author):

The authors engineered an E. coli strain in such a way that glycolysis and the pentose phosphate pathway (PPP) are completely separated from the tricarboxylic acid (TCA) cycle. Glucose was only used for target metabolite production and xylose was used for biomass and energy synthesis, thus suggesting that co-utilization of glucose and xylose leads to the high yield. The resulting engineered strain gave a high yield of 0.31 g MA /g of glucose and 0.35 g L-tyrosine/g of glucose. The manuscript could be considered for publication after addressing the following comments.

Major points:

1. For CFT5 growth, why malate was supplemented along with sodium pyruvate? In your previous study (Noda et al., 2016, Metab. Eng. 33:119-219), the strain CFT5 has grown on M9 minimal medium only with the supplementation of sodium pyruvate, Not malate. It would be better to show whether the strain can grow on minimal medium without any supplementation or supplementation of pyruvate and/or malate separately.
2. Why CFT5 did not grow in xylose medium when XylA and XylB are active? In CFT5, ppc (PEP -->OAA) and maeB (malate -->Pyruvate) are active while indigenous XylAB can metabolize xylose through PPP. The mere assumption of "insufficient activity of xylAB (Line #248-250)" is not convincing without any supportive experimental data from experiments with xylAB overexpression or heterologous xylAB.
3. Even with the supplementation of pyruvate and malate, there was a very long lag phase (~48 h) in Suppl. Fig. 2. Discuss that.
4. Line 194-196: These lines are not clear. When the CFT5 strain is not growing in glucose containing minimal medium, then how come the eda deleted strain (GXa, i.e. CFT5 Δeda) can grow even in the absence of Dahms pathway?
5. Why eda was deleted when assuming that yjhG could catalyze the EDD-like reactions?
6. Line #129-130, the maximum yield was 0.35 g/g glucose by fed batch (Zhang et al., 2015). This yield was obtained at 72 h of cultivation. In the present study 0.31 g/g yield was obtained at 80 h of cultivation. What is the advantage of the present study?
7. It would be better and more valid if the author could show the production of the target compound(s) in both control strain and the final engineered strain. As mentioned in Line # 142-144 (Our final goal in the present study was to construct an engineered strain that can simultaneously assimilate both glucose and xylose and produce target chemicals with high yields), the final strain could be evaluated or compared with the control strains like a glucose and xylose co-utilizing strain (ptsG deleted strain).
8. Why auxotrophic to many aminoacids strain was selected as a parental strain for this study? This phenotype "ptsHI::P A1lacO-1 -glk-galP ΔpykFΔpykA" seems to be sufficient for a parental strain.
9. The present study was conducted in highly aerobic condition (10 mL culture in 200-mL flask with shaking at 220 rpm). What would happen if the experiment needs to be conducted in micro-aerobic or anaerobic conditions? Can we expect the similar performance of PMPE strains at these conditions?
10. Many researchers discussed about glycolaldehyde from Dahms pathway can be utilized by one or several additional pathways. Then how the authors are so sure about the conversion of xylose into pyruvate and glyoxylate? Moreover, indigenous lactaldehyde dehydrogenase (aldA) is not enough for the conversion of glycolaldehyde into glyoxylate discussed in other research (Cabulong et al. Appl Microbiol Biotechnol. 2018:102(5):2179-2189.). Authors also need to mention the fate of this xylose pathway intermediate.
11. The strategy was carried out in the present work is related with glucose to target product and xylose to biomass and energy. What about the inverse of present work where glucose will go to biomass/energy and xylose will go to the target product? Author should discuss and justify the betterment of their approach.
12. How are the ATP, NADH, and NADPH pools changed compared to the wild type? Calculate which

pool theoretically increased / decreased compared to the wild type. Even if growth rates are similar, changes in these pools will have a significant impact on applying this strategy to various productions.

Minor points:
1. Line#215: “In the pyk-knockout strain, the activities of Ppc and Pyk are higher…” – Correct the sentence.
2. Line #216: Pyk or Pck?
3. You have argued that the PMPE strain shows 'sufficient growth for production' in this paper. However, there is no comparison with wild type throughout the article. Compare the similarity of growth to wild type E. coli.
4. Authors need to incorporate the data with wild type strain in Fig 2, 4 and 5. This result will improve the discussion with respect to indigenous xylose utilization capacity of E. coli.
5. In fig 5a, the initial carbon concentrations differ significantly between Gx1xMA and CFT5xMA (almost 20% for xylose). The experiment should be repeated to ensure that the two concentrations match. Otherwise, the production data of fig 5b will also be unreliable.
6. Fig. 7a and 7h: When the cell is not growing on xylose (red line), then how the cell gradually producing xylonate over time from 0 to 72h?
7. Fig 7i: What is the final sampling time? Show the exact time. Because the maximum yield was obtained at the final time point.
8. In Fig 7, authors have discussed the extracellular xylonate reduces the pH in the medium which resulted in reduced cell growth and muconic acid production and this depletion can be compensated through CaCO3. What about intracellular accumulation of xylonate? Authors also need to quantify the intracellular accumulation of xylonate and its impact on growth. Can the incorporation of CaCO3 also settle down the effect of intracellular xylonate accumulation?
9. Line #152: The reference for the CFT5 strain may be corrected. It is not #25.
10. Line # 379: “fusion protein”…There is no prior description on this in introduction or results and discussion section.
11. It would be better if the authors describe the other xylose catabolizing pathway(s) such as Weinberg pathway and rationale for selecting the Dahmns pathway for the study?
12. It would be better if the authors could demonstrate or evaluate the engineered strain to produce MA from a real lignocellulosic biomass?
13. Title of the manuscript mentioned in the supplementary file is different from that of the title mentioned in the main text.
14. One disadvantage of the present strategy has been disused in the results of Fig 7, where the engineered strain can only work under the strict ratio of glucose and xylose. How this disadvantage can be compensated?

Reviewer #2 (Remarks to the Author):

This manuscript describes a new metabolic engineering approach of parallel metabolic pathway engineering (PMPE) for the production of shikimate pathway derivatives (including muconic acid (MA) and aromatic amino acids etc.) from a mixture of glucose and xylose. In this approach, the glycolysis with pentose phosphate pathway (PPP) was completely decoupled from the TCA cycle, where the former is utilized to break down glucose, while the latter is utilized to break down xylose via pyruvate and glyoxylate. It was shown that 4.09 g/l of MA was produced with a yield of 0.31 g/g-glucose by PMPE E.coli stain. This approach is attractive from the application point of view. My comments are as follows:

1. As for biomass formation from xylose, 1 mole of xylose is converted to 1 mole of pyruvate and 1 mole of glyoxylate via Dahms pathway, where pyruvate predominantly goes down to AcCoA. Since ppc and pck genes were knocked out in GX1xMA strain, glyoxylate pathway may be activated by the phosphorylation of ICDH. The malate synthase (MS) of the glyoxylate pathway requires equal moles of

AcCoA and glyoxylate to form 1 mole of malate. However, AcCoA is also necessary for the citrate synthase (CS) reaction to form citate, the stating metabolite of the TCA cycle. Thus, the glyoxylate may accumulate, which may cause the upper steam metabolite such as xylonate to accumulate ? The detailed metabolic flux analysis should be made to clarify this.
2. Although it was claimed that the cell growth is responsible to the availability of (controlled by) xylose, Fig.7a does not necessarily show it ? This may be due to the constraint on the flux of the TCA cycle as mentioned above ?
3. It is not clear how the mass balance is satisfied. For example, in Fig.5, 10 g/l of glucose was converted to about 1.6 g/l of MA (10 g/l of xylose was converted to about 4 of OD of biomass at the same time). Please confirm that that the mass balance is satisfied by showing other metabolites. Cell concentration should be given in g/l.
4. The same is true for Figs.7 and 8.
5. In the pyk mutant strain (together with ppc and pck knockout), PEP may accumulate, which inhibits Pfk activity, activates FBPase, and thus G6P accumulates, and in turn the oxidative PPP may be activated. Then intra-cellular metabolite concentrations should be checked.
6. In addition, the cofactor such as NADPH is necessary for the cell synthesis as well as shikimic acid synthetic pathway, and thus the flux analysis around G6P branch point should be made together with cofactor balance.
7. Acetate was produced at higher xylose content (75 % and 100 %) as shown in Fig.7f. It should be made clear about the mechanism on how the overflow occurs in relation to the imbalance between the PDH flux and the TCA cycle flux. Pyruvate may also activate Pta (and Pox).
8. The lactate formation occurs at higher xylose content (75 % and 50 %) as shown in Fig.7g. This may be caused by the higher redox ratio (NADH/NAD+), and some discussion may be necessary on this. I also wonder if the current culture condition using flask guarantees full aeration ? Otherwise, ArcA may repress the TCA cycle activity ?
9. Since some metabolites such as R5P, E4P, 3PG, and PEP etc. are the precursors of biosynthesis, the arrow from glycolysis and PPP to biomass constituents may be necessary in Fig.1b ?
10. The performance of MA production was evaluated in terms of titer and yield. How about productivity ?
11. In Fig.7i, the concentrations of biomass, xylonate, acetate, and lactate etc. should also be shown.
12. In Fig.8, the concentrations of bi-products should also be shown.
13. It is better to summarize the data as given in Fig.7 in terms of the specific rates such as the specific growth rate, specific substrate consumption rates, and specific metabolite production rates with respect the xylose content.
14. Although it is decomposed between glycolysis with PPP and TCA cycle, some transcription factors such as Cra (and cAMP-Crp) affects the TCA cycle and glyoxylate pathway gene expression in response to FBP/F1P ? Thus, they are indirectly connected ?
15. The drawback of the present approach is that the cell growth is stopped when either glucose or xylose was used as a single carbon source, where this may happen even when a mixture of multiple carbon sources was used, in which one carbon source was consumed fist, and depleted.

Although the proposed approach is attractive from the application point of view, basic question may arise as to the metabolic regulation as compared to the wild-type stain. Namely, as the cell growth rate increases, the metabolism changes from full respiration (oxidative phosphorylation) to respiro-fermentative metabolism with increasing the glycolytic flux to enhance the substrate-level phoshorylation in the wild-type strain. This may be due to oxidative stress regulation. Thus, the glycolysis with PPP must be tightly connected from such point of view, and I wonder if the current stain can endure long-term fermentation without such mechanism ?

Reviewer #3 (Remarks to the Author):

The authors have developed an interesting and novel approach for biochemical production that they

term “parallel metabolic pathway engineering” (PMPE). Via PMPE, glucose is mainly for target chemical production and xylose for supplying essential metabolites for cell growth via an introduced Dahms pathway. To develop this methods, they have performed a systematic characterization of carbon flow through central metabolism in different backgrounds, ultimately landing on the set of mutations required to partition carbon from two different sources. 13C labeling studies are performed to prove their hypotheses in this regard. The approach could be applicable to the production of other chemicals, which would therefore make it significant. This is a well-planned and executed study, and the article is well-written. There are a shortcomings, however, and points in need of clarficiation.

1. The authors chose to demonstrate their method via the production of 2 shikimate pathway derived products. While convenient, this doesn’t really do much to demonstrate ‘versatility’, which was their objective. They fact that PMPE improved tyrosine production after it was shown to improve MA production was not surprising at all. While useful to test, this doesn’t satisfy the objective. And it is an important objective. One Line 107, among central carbon intermediates, PEP was considered the most important intermediate. Why would this be so? Would this not depend on what one is trying to produce? If the authors truly want to demonstrate the versatility of their method, they should really apply it to the production of another non-shikimate and non-PEP derived compound. The discuss 1,2-propanediol as a possible candidate. If PMPE is truly to be presented as a versatile and important ‘strategy’ for metabolic engineering, it is not sufficient to simply save this for future, in my opinion.
2. The choice of shikimate pathway derived compounds for demonstrating PMPE is a bit peculiar to begin with, since it is well known that the yields/titers of such products is much higher from xylose (due to higher levels of PEP and E4P). That is, using xylose to produce such compounds and glucose for growth would perhaps be the optimal strategy. Of course, that would require an entirely different set of parallel pathways. However, it does beg that question: what are the optimal product targets of this strategy? This requires some thinking and discussion, since it currently seems as though the products were selected just based on convenience.
3. Fig 2: Panel C is not essential, as it contains the same data as in B.
4. In CFT5x, xylose was not fully consumed in 96 h. Was it ever? How to improve in this strain?
5. The observation that GX1x did not grow in M9 minimal media with just xylose was surprising. We routine grow ATCC31882 delta(pykAF) in xylose minimal media. It seems surprising that the additional mutations would inhibit this. Any thoughts on this? In the absence of glucose, native xylose metabolism should be quite active. This likely requires some additional discussion. Also, was this truly minimal media or was yeast extract added? It seems that this strain also carries auxotrophic mutations. Please be clear about this point throughout the paper.

Responses to the comments by reviewer #1

Thank you for your thoughtful comments that have helped us to improve our manuscript considerably. We revised our manuscript according your suggestion as follows.

Major points:

1. For CFT5 growth, why malate was supplemented along with sodium pyruvate? In your previous study (Noda et al., 2016, Metab. Eng. 33:119-219), the strain CFT5 has grown on M9 minimal medium only with the supplementation of sodium pyruvate, Not malate. It would be better to show whether the strain can grow on minimal medium without any supplementation or supplementation of pyruvate and/or malate separately.

Response

In our previous study (Noda et al., 2016, Metab. Eng. 33:119-219), CFT5 was constructed for the production of salicylate and its derivatives. Salicylate synthesis pathway produces pyruvate. CFT5 without salicylate synthesis pathway synthesized only a little amount of pyruvate, which was not enough for cell growth. Thus, we believe that CFT5 did not grow in the medium supplemented pyruvate due to the insufficient availability of extracellular pyruvate.

According to your suggestion, the growth on medium conditions with/without of pyruvate and/or malate was added in Supplementary Fig. S2.

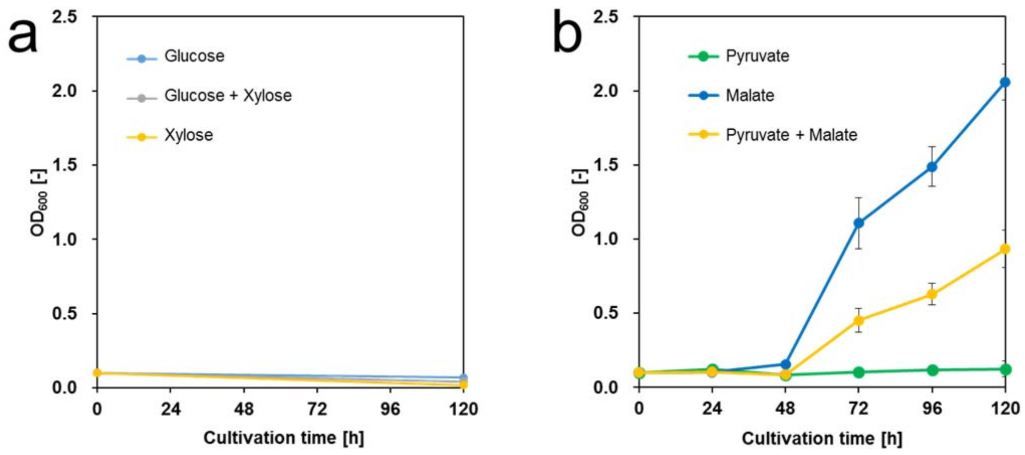

Supplementary Fig. S2. Growth of the CFT5 strain. (A) Blue, gray, and yellow symbols indicate

M9 minimal medium supplemented with glucose, a glucose–xylose mixture, and xylose, respectively. (B) Green, blue, and yellow symbols indicate the use of minimal medium containing glucose supplemented with 10-mM sodium pyruvate, 10-mM sodium malate, and mixture of 5-mM pyruvate and 5-mM malate, respectively. The data are presented as the average of three independent experiments, and error bars indicate standard errors.

2. Why CFT5 did not grow in xylose medium when XylA and XylB are active? In CFT5, ppc (PEP -->OAA) and maeB (malate -->Pyruvate) are active while indigenous XylAB can metabolize xylose through PPP. The mere assumption of "insufficient activity of xylAB (Line #248-250)" is not convincing without any supportive experimental data from experiments with xylAB overexpression or heterologous xylAB.

Response

We overexpressed xylAB and/or Dahms pathway related enzymes in CFT5 strain. All strains could not grow in minimal medium with xylose as a carbon source. Therefore, according to your suggestion, the data of the bacterial cell growth of the strains overexpressing XylAB and/or Dahms pathway enzymes was added (Supplementary Fig. S5) and we revised our manuscript as follows;

It was thought that the GX1x strain did not grow in M9 minimal medium containing only xylose because of the insufficient activity of the endogenous xylose catabolic pathway (i.e., xylAB). We constructed the strain overexpressing XylA and XylB derived from GX1x. Contrary to expectations, this strain did not glow in M9 minimal medium with xylose as a sole carbon source. Furthermore, the strains derived from CFT5 overexpressing Dahms pathway enzymes (Xdh, CcxylC, YjhH, and YjhG) and/or enzymes of the endogenous xylose catabolic pathway (XylAand XylB) also did not grow in the same condition (Supplementary Fig. S6).

(Please, see page 15, line 251-258)

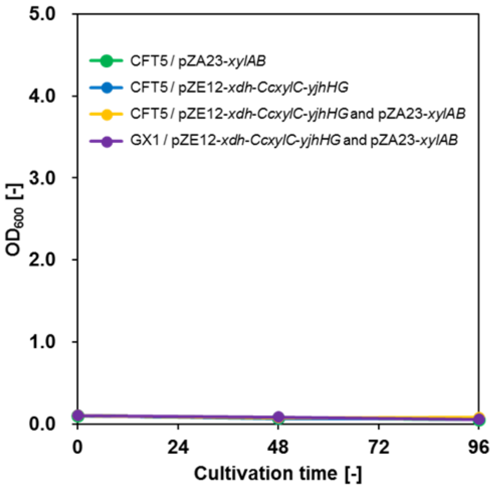

Supplementary Fig. S5. The effect of overexpressing XylA and XylB on the cell growth in M9 minimal medium with xylose as a sole carbon source. Green, blue, and yellow symbols indicate the bacterial cell growth of CFT5 harboring pZA23-xylAB, CFT5 harboring pZE12-xdh-CcxylC-yjhHG, and CFT5 harboring pZE12-xdh-CcxylC-yjhHG and pZA23-xylAB. Purple symbols indicate the bacterial cell growth of GX1 harboring pZE12-xdh-CcxylC-yjhHG and pZA23-xylAB.

3. Even with the supplementation of pyruvate and malate, there was a very long lag phase (~48 h) in Suppl. Fig. 2. Discuss that.

Response

In CFT5 strain, phosphotransferase system (PTS) was deleted and ATP-consuming GalP/Glk system was introduced. In addition, ATP-producing enzymes pykA and pykF were disrupted. These modifications caused low levels of ATP, which might delay the cell growth. Therefore, according to your suggestion, we revised our manuscript as follows;

Alternatively, the growth of the CFT5 strain was recovered in medium containing malate, which is a TCA cycle intermediate (Supplementary Fig. S2b). While the cell growth recovered by adding malate, it needed a very long lag phase, about two days. In CFT5, GalP/Glk system consumes 1 mole of ATP when converting 1 mole of glucose to glucose-6-phosphate. Furthermore, pyruvate kinases coded by pykA and pykF which synthesis pyruvate and ATP from PEP, were disrupted. Therefore, the lower levels of ATP might have delayed cell growth.

(Please, see page 10, line 157-162)

4. Line 194-196: These lines are not clear. When the CFT5 strain is not growing in glucose containing minimal medium, then how come the eda deleted strain (GXa, i.e. CFT5 Δeda) can grow even in the absence of Dahms pathway?

Response

In GXa strain, PEP carboxylase can supply the carbon from PEP into the TCA cycle. We believe that the balance of PEP–OAA interconversion may be altered by the disruption of the ED pathway. According to your suggestion, we revised our manuscript as follows;

This result suggests that the disruption of eda may activate other pathways or provide TCA cycle intermediates. In GXa, ppc and pck which coding PEP carboxylase (Ppc) and PEP carboxykinase (Pck), remained intact. Ppc and Pck catalyze the reaction converting PEP to OAA and the reverse reaction (PEP–OAA interconversion). It is thought that the balance of PEP–OAA interconversion may be altered by the disruption of the ED pathway.

(Please, see page 12, line 207 and page 13, line 208-210)

5. Why eda was deleted when assuming that yjhG could catalyze the EDD-like reactions?

Response

From the results of the additional examination of expressing each enzyme in Dahms pathway separately, we found that the key enzyme might be Xdh, not YjhG. Therefore, we revised our manuscript as follows;

Unexpectedly, CFT5x grew in M9 minimal medium containing glucose as the sole carbon source, suggesting that introducing the Dahms pathway may activate carbon flow into the TCA cycle from glucose but not xylose. We examined the effect on the cell growth by expressing each enzyme of Dahms pathway, Xdh, CcxylC, and yjhHG in CFT5. As a result, only the strain expressing Xdh could grow in M9 minimal medium containing glucose as the sole carbon source (Supplementary Fig. S3). However, Xdh have no or little enzymatic activity for glucose or glucose-6-phosphate

as substrates[32]. Thus, 6-phosphogluconate is one of the possible candidates.

(Please, see page 11, line 188-189 and page12, 190-193)

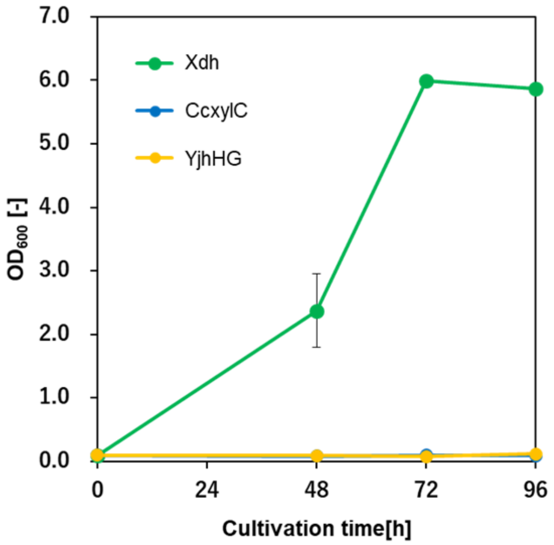

Supplementary Fig. S3. Growth of the CFT5 derived strain expressing each Dahms pathway enzyme in M9 minimal medium containing glucose as a sole carbon source. Green, blue, and yellow symbols indicate the bacterial cell growth of CFT5 harboring pZE12-xdh, CFT5 harboring pZE12-CcxylC, and CFT5 harboring pZE12-yjhHG, respectively.

6. Line #129-130, the maximum yield was 0.35 g/g glucose by fed batch (Zhang et al., 2015). This yield was obtained at 72 h of cultivation. In the present study 0.31 g/g yield was obtained at 80 h of cultivation. What is the advantage of the present study?

Response

One advantage of our approach is the simplicity of the fermentation process. We achieved high yield as batch culture, and slightly lower than the yield of fed batch cultivation reported to date. Therefore, we revised our manuscript as follows;

The highest yields of MA reported till date are 0.21 and 0.35 g/g of glucose in batch culture and complicated fed-batch culture, respectively[25,35,38].

(Please, see page 8, line 125-126)

7. It would be better and more valid if the author could show the production of the target compound(s) in both control strain and the final engineered strain. As mentioned in Line # 142-144 (Our final goal in the present study was to construct an engineered strain that can simultaneously assimilate both glucose and xylose and produce target chemicals with high yields), the final strain could be evaluated or compared with the control strains like a glucose and xylose co-utilizing strain (ptsG deleted strain).

Response

We examined MA production by the control strain you suggested. Deletion of *ptsG* and *pheA* yielded CTR2 as a control strain. Similar to GX1, phenylalanine biosynthetic pathway in CTR2 was inactivated by *pheA* disruption. CTR2MA was constructed by introducing pSAK-ZYc CTR2. Our final strain (GX1xMA) produced MA considerably higher than the control strain CTR2MA. We added these results in our manuscript as follows;

As a control of glucose and xylose co-utilizing strain, strain CTR2 was constructed by disrupting ptsG and pheA, which are responsible for catabolite repression and production of L-phenylalanine competing with MA production, respectively, from ATCC31882. CTR2MA strain (CTR2 harboring an MA-producing plasmid) was cultured in M9 minimal medium and produced 0.53 ± 0.01 g/L of MA after 48h (Supplementary Fig. S8).

(Please, see page 16, line 272-277)

The production titer of MA from glucose of the GX1xMA strain were 2.3- and 3.0-fold higher than CFT5xMA strain and CTR2MA strain, respectively, and the yield was 2.6-fold higher than CFT5xMA.

(Please, see page 17, line 280-282)

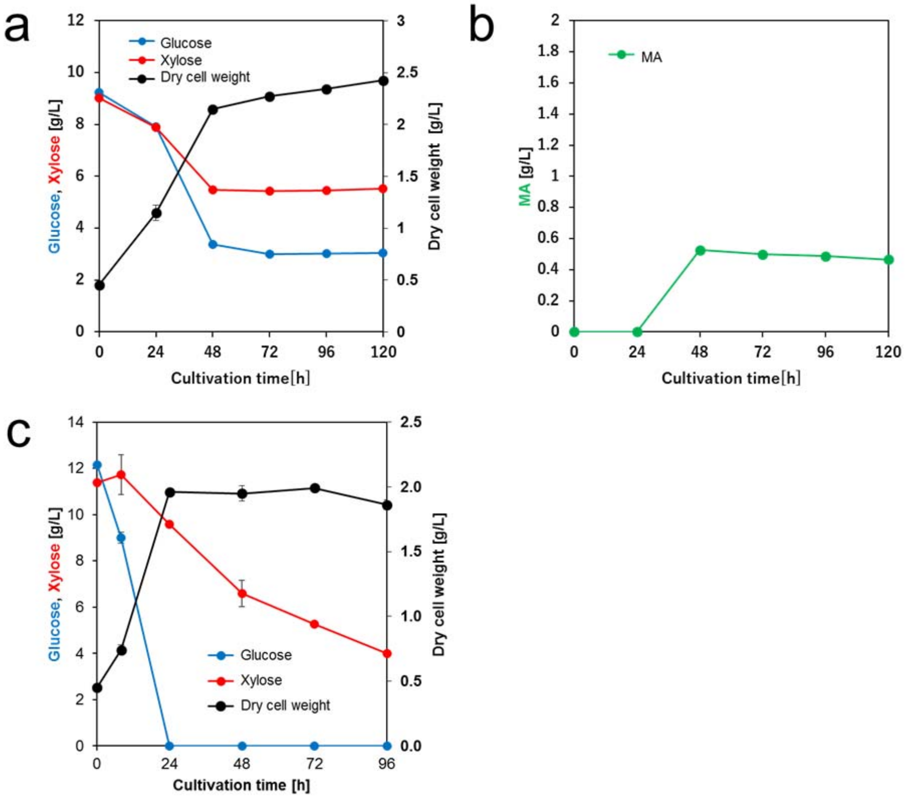

Supplementary Fig. S8. Culture profiles of CTR2MA and ATCC31882 strains in M9 minimal medium. (a) Culture profiles of CTR2MA. Black, blue, and red symbols indicate bacterial cell growth, glucose consumption, and xylose consumption, respectively. (b) MA production in CTR2MA. (c) Culture profiles of ATCC31882. Black, blue, and red symbols indicate bacterial cell growth, glucose consumption, and xylose consumption, respectively. The data are presented as the average of three independent experiments, and error bars indicate standard errors.

8. Why auxotrophic to many aminoacids strain was selected as a parental strain for this study? This phenotype “ptsHI::P A1lacO-1 -glk-galP ΔpykFΔpykA” seems to be sufficient for a parental strain.

Response

Parental strain ATCC31882 were originally designed as phenylalanine overproducing strain constructed by enhancing phenylalanine synthesis pathway (inserted feedback resistance mutation in *pheA*). The existing carbon flux to phenylalanine was competing with the pathway to *cis*,*cis*-muconic acid (MA), our target compound.

9. The present study was conducted in highly aerobic condition (10 mL culture in 200-mL flask with shaking at 220 rpm). What would happen if the experiment needs to be conducted in micro-aerobic or anaerobic conditions? Can we expect the similar performance of PMPE strains at these conditions?

Response

Because MA synthesis pathway (catechol 1,2-dioxygenase) needs oxygen as a cofactor, we examined tyrosine production in micro-aerobic condition. The yield of GX1xTYR was improved up to about 5-fold compared to CFT5xTYR. Therefore, according to your suggestion, we added these results in revised manuscript as follows;

In micro-aerobic condition, GX1xTYR strain and CFT5xTYR strain produced 0.32 ± 0.02 g/L (96 h) and 0.40 ± 0.02 g/L (72 h) of L-tyrosine, respectively, and the production yield in GX1xTYR reached 0.32 ± 0.10 g/g of glucose after 96 h of cultivation, reflecting a 5.22-fold increase compared with that using the CFT5xTYR (Fig. 8). These results suggested that PMPE is a promising strategy to improve production even in micro-aerobic conditions.

(Please, see page 24, line 405-411)

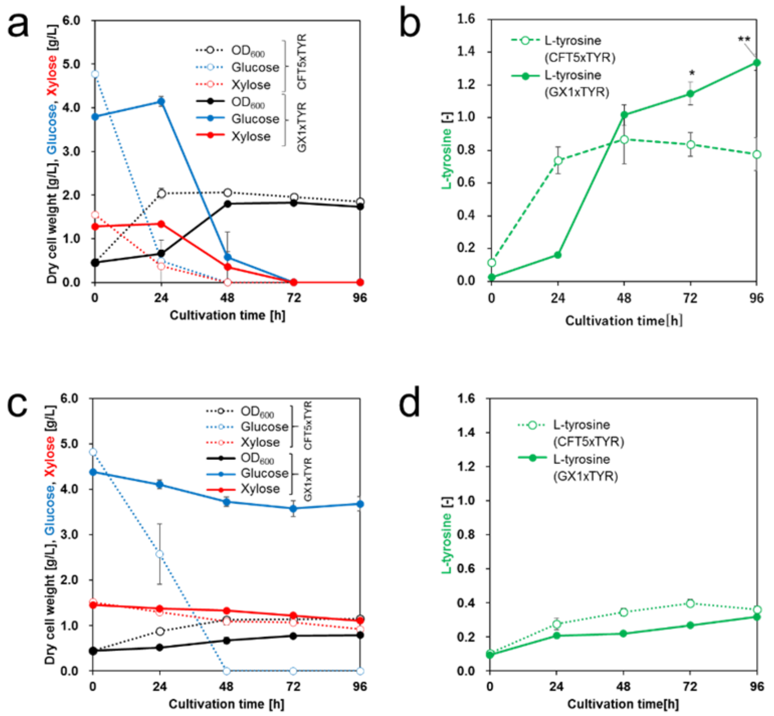

Fig. 8. Cultures profiles of L-tyrosine-producing strains. (a, b) aerobic condition. (c, d) micro-aerobic condition. (a, c) Black, blue, and red symbols indicate bacterial cell growth, glucose consumption, and xylose consumption, respectively. (b, d) Green symbols indicate the produced amounts of L-tyrosine. All open symbols and filled symbols indicate the results for the CFT5xTYR and GX1xTYR strains, respectively. The data are presented as the average of three independent experiments, and error bars indicate standard errors. P values were computed using the two-tailed Student's t-test (, P < 0.05; **, P < 0.01).*

10. Many researchers discussed about glycolaldehyde from Dahms pathway can be utilized by one or several additional pathways. Then how the authors are so sure about the conversion of xylose into pyruvate and glyoxylate? Moreover, indigenous lactaldehyde dehydrogenase (aldA) is not enough for the conversion of glycolaldehyde into glyoxylate discussed in other research (Cabulong et al. Appl Microbiol Biotechnol. 2018:102(5):2179-2189.). Authors also need to mention the fate of this xylose pathway intermediate.

Response

Glycolaldehyde can be metabolized to other than glyoxylate such as ethylene

glycol. However, since considerable glyoxylate is accumulated in GX1xMA strain (Supplementary Fig. S11), it is thought that most of glycolaldehyde flows to glyoxylic acid. According to your suggestion, we revised our manuscript as follows;

Xylonate accumulation is a common problem of Dahms pathway-mediated chemical production [33,34,48,49], which decreases the yields of target compounds synthesized via this pathway. To reduce the accumulation of xylonate, overexpressing lactaldehyde dehydrogenase (AldA) would be one of the possible strategies. Glycolaldehyde synthesized via Dahms pathway is converted to glycolate by AldA and then glycolate is converted to glyoxylate by glycolate oxidase. Cabulong et al. increased glycolic acid production by overexpressing AldA[33]. In PMPE strains, it might be possible to improve the accumulation of xylonate that pulling the carbon flow from glycolaldehyde to glyoxylate by overexpressing AldA and glycolate oxidase.

(Please, see page 21, line 358-364)

11. The strategy was carried out in the present work is related with glucose to target product and xylose to biomass and energy. What about the inverse of present work where glucose will go to biomass/energy and xylose will go to the target product? Author should discuss and justify the betterment of their approach.

Response

There is more glucose than xylose in nature, and it would be industrially advantageous to use glucose for the production of useful chemicals. When xylose is used for target metabolite production via Dahms pathway (or Weimberg pathway), several types of compounds can be produced such as xylonate, ethylene glycol, glycolic acid, 1,2,4-butanetriol, and so on, which are the intermediate or derivatives of Dahms pathway. However, many valuable compounds derived from shikimate pathway, PPP, and glycolysis pathway cannot be produce from xylose via Dahms pathway.

12. How are the ATP, NADH, and NADPH pools changed compared to the wild type? Calculate which pool theoretically increased / decreased compared to the wild type. Even if growth rates are similar, changes in these pools will have a significant impact on applying this strategy to various productions.

Response

We performed metabolome analysis of constructed strains and parental strain (Supplementary Discussions: Metabolome analysis of PMPE strains). The ATP levels of PMPE strains were lower than parental strain. In addition, the NADH level of PMPE strains also lower. These results suggest that limited NADH regeneration in TCA cycle caused reduction of ATP synthesis via oxidative phosphorylation. As expected, lower levels of NADPH of PMPE strain, due to NADPH consumption by enhanced shikimate pathway. Therefore, according to your suggestion, we added these results in our manuscript as follows;

We also investigated the levels of ATP, ADP, AMP, NADH, NAD^+, NADPH, and $NADP^+$. In GX1x, the ATP level was significantly reduced compared to ATCC31882x and CFT5 (Supplementary Fig. S14a). The NADH level in GX1x was significantly reduced compared to ATCC31882x, and NADH was not detected in CFT5x (Supplementary Fig. S14b). These results show that ATP synthesis by oxidative phosphorylation did not work well due to the restriction of the NADH regeneration in the TCA cycle. Additionally, the NADPH in CFT5x and GX1x were not detected, which caused by the enhanced shikimate pathway because shikimate dehydrogenase consumes 1 mole of NADPH when synthesis 1 mole of shikimate from 3-dehydroshikimate.

(Please, see Supplementary Information page 2, line 40-49)

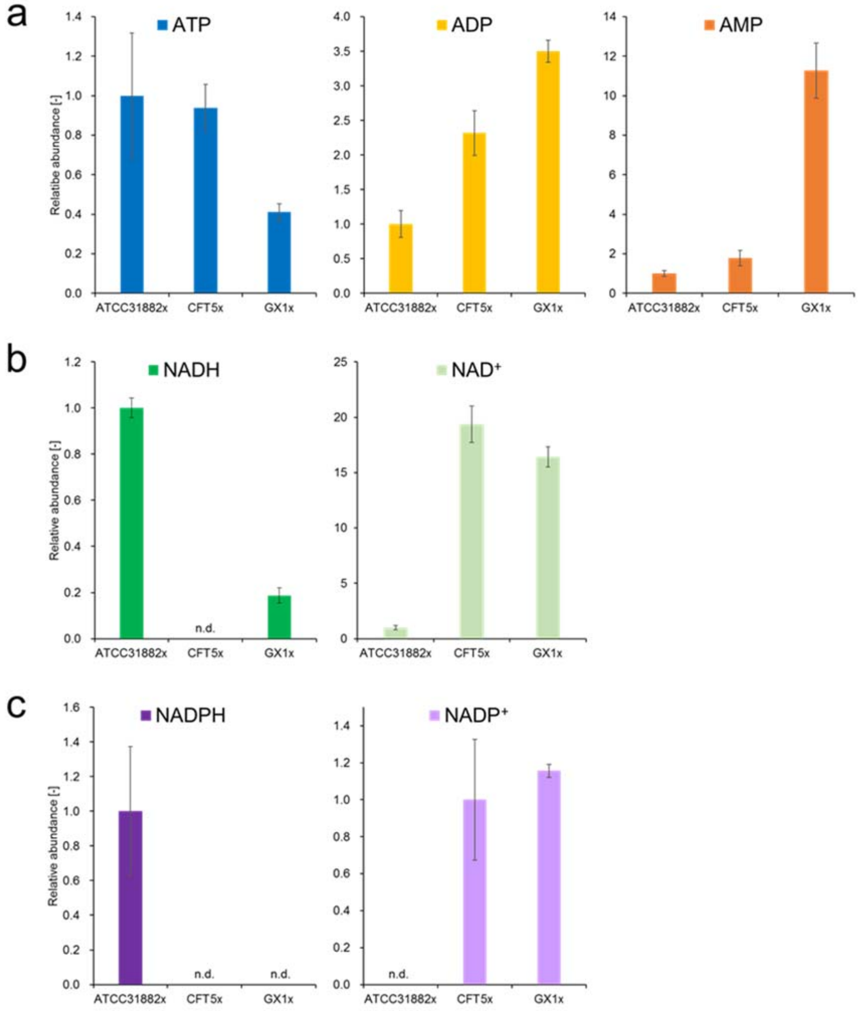

Supplementary Fig. S14. The relative abundance of ATP, ADP, AMP, NADH, NAD^+, NADPH, and $NADP^+$ in PMPE strains. (a) Blue, yellow, and orange bars indicate ATP, ADP, and AMP, respectively. (b) Dark green, and light green bars indicate NADH, and NAD^+, respectively. (c) Dark purple, and light purple bars indicate NADPH, and $NADP^+$, respectively. Figures indicate the specific accumulated amount of metabolites per OD_{600}. CFT5x was used as a benchmark in $NADP^+$ and ATCC31882x was used as a benchmark in other metabolites. n.d., not detected. The data are presented as the average of three independent experiments and error bars indicate standard deviations.

Minor points:

1. Line#215: "In the pyk-knockout strain, the activities of Ppc and Pyk are higher…" – Correct the sentence.

Response

As you pointed out, we revised our manuscript as follows;

In the pyk-knockout strain, the activities of Ppc and Pck are higher than those in the wild-type strain [42].

(Please, see page 13, line 218-219)

2. Line #216: Pyk or Pck?

Response

As you pointed out, we revised our manuscript as follows;

Ppc and Pck activity, i.e., PEP–OAA interconversion, depends on the concentration of metabolites in the TCA cycle (i.e., PEP, OAA, acetyl-CoA, and malate), and it is regulated by these metabolites[43,44].

(Please, see page 13, line 221-223)

3. You have argued that the PMPE strain shows 'sufficient growth for production' in this paper. However, there is no comparison with wild type throughout the article. Compare the similarity of growth to wild type E. coli.

Response

We added the culture profiles of wild type cultured in glucose, xylose, and these mixture medium. The maximum cell density of GX1x was at the same level of ATCC31882. Detailed revises are indicated in the response of the next comment.

4. Authors need to incorporate the data with wild type strain in Fig 2, 4 and 5. This result will improve the discussion with respect to indigenous xylose utilization capacity of E. coli.

Response

As you pointed out, we revised our manuscript as follows;

GX1x strain equipped with the Dahms pathway (Fig. 4a) grew in M9 minimal medium with

a glucose–xylose mixture as the carbon source, and maximum OD_{600} reached at the same level of ATCC31882 (Fig. 4b).

(Please, see page 15 line 244-246)

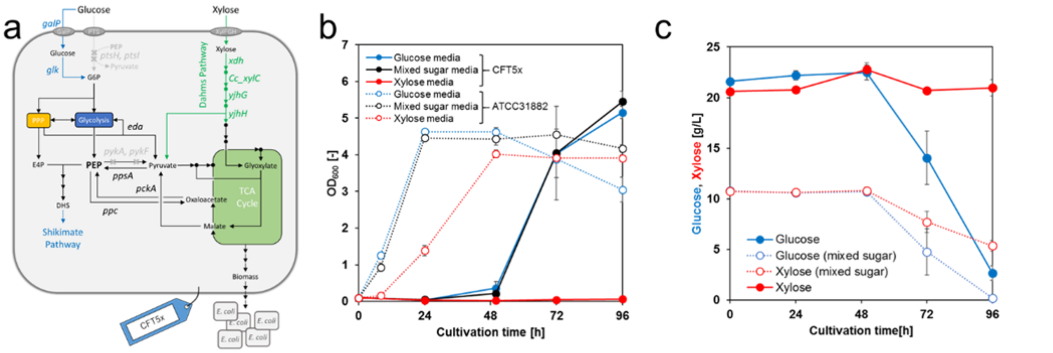

*Fig. 2. Strain construction and cell growth examination. (**a**) Metabolic design of CFT5x. (**b**) Bacterial cell growth. Blue, black, and red symbols indicate the growth of the CFT5x strain and ATCC31882 cultured in medium supplemented with glucose, a glucose–xylose mixture, and xylose, respectively. (**c**) Glucose and xylose consumption. Filled blue symbols indicate glucose consumption in glucose-containing medium. Filled red symbols indicate xylose consumption in xylose-containing medium. Open blue and open red symbols indicate glucose and xylose consumptions in mixed sugar-containing medium, respectively. G6P, glucose-6-phosphate; PEP, phosphoenolpyruvate; E4P, erythrose 4-phosphate; DHS, dehydroshikimate; and Gluconate-6P, gluconate-6-phosphate. The data are presented as the average of three independent experiments, and error bars indicate standard errors.*

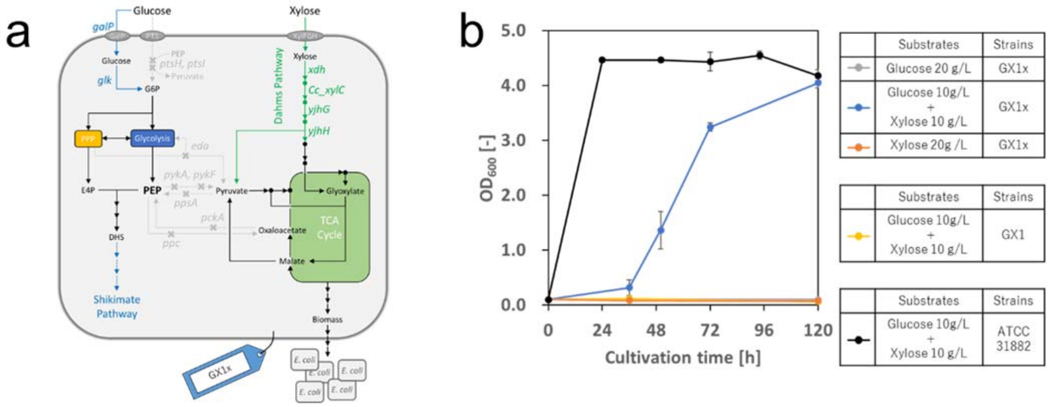

*Fig. 4. Examination of the growth of the strain in which the Dahms pathway was introduced. (**a**) Metabolic design of the GX1x strain. (**b**) Bacterial cell growth in minimal medium.*

Gray, blue, and orange symbols indicate the growth of the GX1x strain cultured in medium containing only glucose, a glucose–xylose mixture, and xylose alone, respectively. Yellow symbols indicate the growth of the GX1 strain (i.e., a strain lacking the Dahms pathway) cultured in medium containing a glucose–xylose mixture. Black symbols indicate the growth of the ATCC31882 (control strain) cultured in medium containing a glucose–xylose mixture. G6P, glucose-6-phosphate; PEP, phosphoenolpyruvate; E4P, erythrose 4-phosphate; and DHS, dehydroshikimate. The data are presented as the average of three independent experiments, and error bars indicate standard errors.

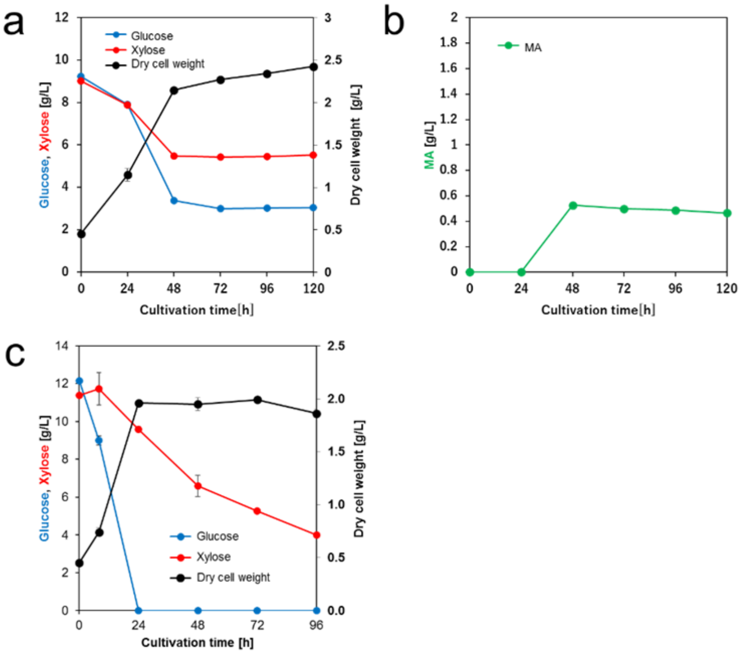

Supplementary Fig. S8. Culture profiles of CTR2MA and ATCC31882 strains in M9 minimal medium. (a) Culture profiles of CTR2MA. Black, blue, and red symbols indicate bacterial cell growth, glucose consumption, and xylose consumption, respectively. (b) MA production in CTR2MA. (c) Culture profiles of ATCC31882. Black, blue, and red symbols indicate bacterial cell growth, glucose consumption, and xylose consumption, respectively. The data are presented as the average of three independent experiments, and error bars indicate standard errors.

5. In fig 5a, the initial carbon concentrations differ significantly between Gx1xMA and CFT5xMA (almost 20% for xylose). The experiment should be repeated to ensure that the two concentrations match. Otherwise, the production data of fig 5b will also be unreliable.

Response

As you pointed out, we corrected the data of xylose concentration based on the results of the re-measurement of the same samples. Fig. 5a was revised as follow.

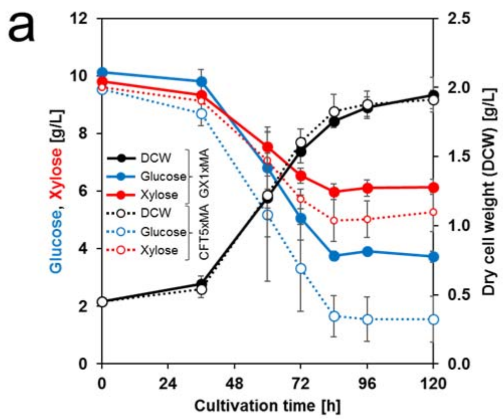

Fig. 5. Culture profiles of cis,cis-muconic acid (MA)-producing strains in M9 minimal medium. (a) Black, blue, and red symbols indicate bacterial cell growth, glucose consumption, and xylose consumption, respectively. (b) Green symbols indicate the produced amounts of MA. All open and filled symbols indicate the results for the CFT5xMA and GX1xMA strains, respectively. The data are presented as the average of three independent experiments, and error bars indicate standard errors. P values were computed using the two-tailed Student's t-test (, P < 0.05).*

6. Fig. 7a and 7h: When the cell is not growing on xylose (red line), then how the cell gradually producing xylonate over time from 0 to 72h?

Response

It is thought that cells worked as whole-cell catalysis. Xylose dehydrogenase requires NAD^+ as a cofactor. In the PMPE strain, NAD^+ was considerably increased than ATCC31882 (Supplementary Fig. S14b). Thus, xylonate production was occurred without cell growth.

(Fig 7h has been moved to Supplementary Fig. S11d.)

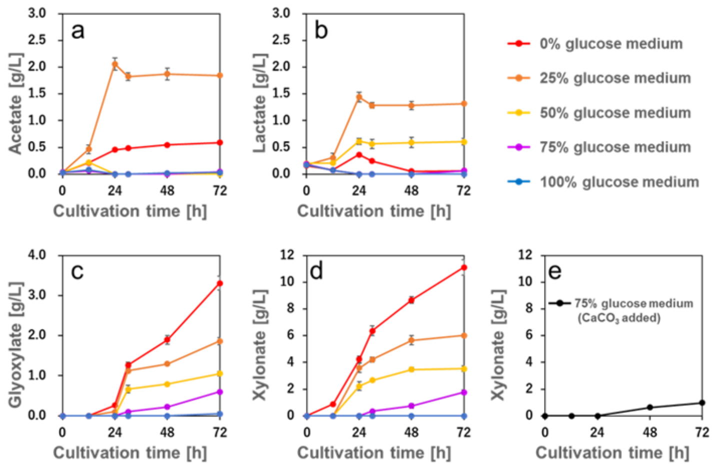

Supplementary Fig. S11. Culture profiles of the GX1xMA strain in M9 minimal medium containing 20-g/L sugar(s) and 5-g/L yeast extract. (a) Acetate accumulation. (b) Lactate accumulation. (c) Glyoxylate accumulation. (d) Xylonate accumulation. (e) Xylonate accumulation in 75% glucose medium with $CaCO_3$ addition condition. Red, orange, yellow, purple, and blue symbols indicate the results of cultivation in 0%, 25%, 50%, 75%, and 100% glucose medium, respectively, in a–d. The data are presented as the average of three independent experiments, and error bars indicate standard errors.

7. Fig 7i: What is the final sampling time? Show the exact time. Because the maximum yield was obtained at the final time point.

Response

As you pointed out, we revised our manuscript as follows;

(Fig 7i has been renamed to Fig. 7f.)

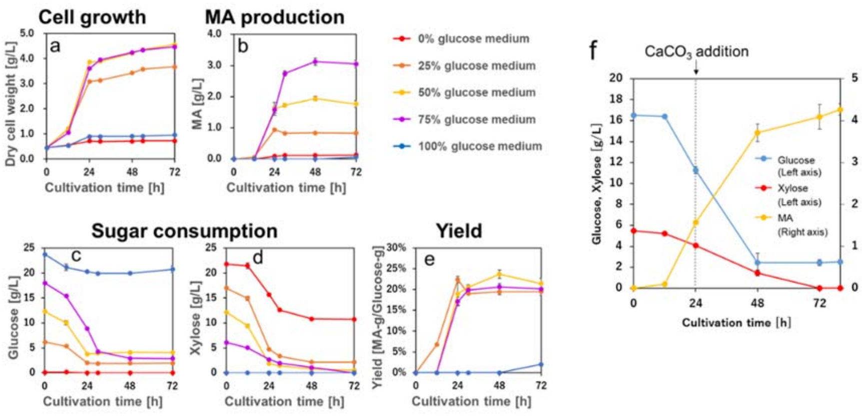

Fig. 7. Culture profiles of the GX1xMA strain in M9 minimal medium containing 20-g/L sugar(s) and 5-g/L yeast extract. (a) Bacterial cell growth. (b) Produced amounts of MA. (c) Glucose consumption. (d) Xylose consumption. (e) Production yield of cis,cis-muconic acid (MA) from glucose (g of produced MA/g of consumed glucose). Red, orange, yellow, purple, and blue symbols indicate the results of cultivation in 0%, 25%, 50%, 75%, and 100% glucose medium, respectively, in a–h. ***(f)*** *Culture profiles of the GX1xMA strain with the addition of $CaCO_3$. The final sampling time is after 80h cultivation. Blue, red, and yellow symbols indicate the glucose concentration, xylose concentration, and the produced amount of MA, respectively. The data are presented as the average of three independent experiments, and error bars indicate standard errors.*

8. In Fig 7, authors have discussed the extracellular xylonate reduces the pH in the medium which resulted in reduced cell growth and muconic acid production and this depletion can be compensated through CaCO3. What about intracellular accumulation of xylonate? Authors also need to quantify the intracellular accumulation of xylonate and its impact on growth. Can the incorporation of CaCO3 also settle down the effect of intracellular xylonate accumulation?

Response

We believe that synthesized xylonate is immediately transported extracellularly. In Fig 7 and Supplementary Fig. S11, when cultured in 100% xylose medium (red line), over 90% of the consumed xylose is converted xylonate and accumulated extracellular. In addition, wild type *E. coli* can use xylonate as a substrate for cell growth[1], suggesting the xylonate transportation between medium and inside of *E. coli* cell is efficient[2].

9. Line #152: The reference for the CFT5 strain may be corrected. It is not #25.

Response

As you pointed out, we revised our manuscript as follows;

Regarding potential parent strains, we chose the previously developed E. coli CFT5 strain[41]. (41. Noda, S. et al., Metab. Eng. 33, 119–129 (2016).)

(Please, see page 9, line 149)

10. Line # 379: “fusion protein”…There is no prior description on this in introduction or results and discussion section.

Response

As you pointed out, we revised our manuscript as follows;

We solved this problem by expressing the protein fused chorismate synthase and isochorismate synthase in MA producing strain in this study.

(Please, see page 25, line 424-425)

11. It would be better if the authors describe the other xylose catabolizing pathway(s) such as Weinberg pathway and rationale for selecting the Dahmns pathway for the study?

Response

In previous studies, when the salicylate synthesis pathway which supplies pyruvate was introduced, the CFT5 strain grew on a minimal medium. Based on these results, Dahms pathway was considered suitable because it was possible to restore cell growth by supplying pyruvate from xylose. However, we found that PMPE using the Dahms pathway has problems such as xylonate and glyoxylate accumulation. Using the Weimberg pathway instead of the Dahms pathway has the potential to solve these problems. Therefore, according to your suggestion, we revised our manuscript as follows;

Glyoxylate accumulation is another issue to be solved. Using Weimberg pathway is alternative option because this pathway supplies α-ketoglutarate from xylose without producing glyoxylate.

(Please, see page 26, line 444-446)

12. It would be better if the authors could demonstrate or evaluate the engineered strain to produce MA from a real lignocellulosic biomass?

Response

In order to evaluate MA production by the PMPE strain from real lignocellulosic biomass, it is necessary to consider not only fermentation processes but also pretreatment and saccharification processes. As shown in Fig. 7, the tuning of the culture

conditions has a great influence on the production results, so a lot of additional experiments must be performed. However, as you pointed out, MA production from real lignocellulosic biomass is one of the challenging research topics in aspect to develop a clean process. We will undertake that theme in our future works.

13. Title of the manuscript mentioned in the supplementary file is different from that of the title mentioned in the main text.

Response

As you pointed out, we revised our manuscript as follows;

Metabolic Engineering of Escherichia coli for shikimate pathway derivative production from glucose–xylose co-substrate

(Please, see Supplementary Information page 1, line 3-4)

14. One disadvantage of the present strategy has been disused in the results of Fig 7, where the engineered strain can only work under the strict ratio of glucose and xylose. How this disadvantage can be compensated?

Response

It is thought that PMPE strains can only grow sufficiently with a narrow range of substrates ratio because the carbon flux derived from xylose is stagnant at xylonate and glyoxylate. Indeed, there is room for improvement on the xylose catabolic pathway including Dahms pathway. As you pointed out, the overexpression of AldA has the potential to improve the metabolism of xylose. Accumulation of glyoxylate caused by the shortage of acetyl-coA synthesized from pyruvate needs to be reduced. It would be improved by disrupting Pta which are enzymes catalyzing the reaction convert pyruvate to acetyl phosphate and overexpression of malate dehydrogenase, which converts malate to pyruvate. We revised our manuscript as follows;

To reduce the accumulation of xylonate, overexpressing lactaldehyde dehydrogenase (AldA) would be one of the possible strategies. Glycolaldehyde synthesized via Dahms pathway is converted to glycolate by AldA and then glycolate is converted to glyoxylate by glycolate oxidase. Cabulong et al. increased glycolic acid production by overexpressing AldA[33]*. In PMPE strains, it might be possible to*

improve the accumulation of xylonate that pulling the carbon flow from glycolaldehyde to glyoxylate by overexpressing AldA and glycolate oxidase. Another explanation of the glyoxylate accumulation is due to the shortage of acetyl-CoA. Glyoxylate and acetyl-CoA are converted to malate by malate synthase. Disruption of Pta and overexpression of malate dehydrogenase, which converts malate to pyruvate, have the potential to solve glyoxylate accumulation.

(Please, see page 21, line 358-368)

Responses to the comments by reviewer #2

Thank you for your thoughtful comments that have helped us to improve our manuscript considerably. We revised our manuscript according your suggestion as follows.

Reviewer #2 (Remarks to the Author):

This manuscript describes a new metabolic engineering approach of parallel metabolic pathway engineering (PMPE) for the production of shikimate pathway derivatives (including muconic acid (MA) and aromatic amino acids etc.) from a mixture of glucose and xylose. In this approach, the glycolysis with pentose phosphate pathway (PPP) was completely decoupled from the TCA cycle, where the former is utilized to break down glucose, while the latter is utilized to break down xylose via pyruvate and glyoxylate. It was shown that 4.09 g/l of MA was produced with a yield of 0.31 g/g-glucose by PMPE E.coli stain. This approach is attractive from the application point of view. My comments are as follows:

1. As for biomass formation from xylose, 1 mole of xylose is converted to 1 mole of pyruvate and 1 mole of glyoxylate via Dahms pathway, where pyruvate predominantly goes down to AcCoA. Since ppc and pck genes were knocked out in GX1xMA strain, glyoxylate pathway may be activated by the phosphorylation of ICDH. The malate synthase (MS) of the glyoxylate pathway requires equal moles of AcCoA and glyoxylate to form 1 mole of malate. However, AcCoA is also necessary for the citrate synthase (CS) reaction to form citate, the stating metabolite of the TCA cycle. Thus, the glyoxylate may accumulate, which may cause the upper steam metabolite such as xylonate to accumulate ? The detailed metabolic flux analysis should be made to clarify this.

Response

We measured the concentration of glyoxlate in the medium of flask fermentation. The malate synthase (MS) requires 1 mole of AcCoA (from pyruvate) and 1 mole of glyoxylate for synthesis of 1 mole of malate. Malate is converted to oxaloacetate by malate dehydrogenase coded by *mqo* or *mdh*, or converted to pyruvate with exhausting CO_2 by malate dehydrogenase coded by *sfcA* or *maeB*. Thus, by the reaction catalyzed by SfcA and MaeB, pyruvate is regenerated. By enhancing the reaction converting malate to pyruvate, theoretically, it is possible that 1 mole of citrate can be generated from 2 moles of xylose (= 2 moles of pyruvate and 2 moles of glyoxylate) via Dahms pathway without accumulating glyoxylate. Therefore, according to your suggestion, we added the

results and discussions as follows;

Furthermore, the accumulation of xylonate and glyoxylate increased in proportion to xylose concentration (Supplementary Fig. S11).

(Please, see page 21, line 353-354)

Another explanation of the glyoxylate accumulation is due to the shortage of acetyl-CoA. Glyoxylate and acetyl-CoA are converted to malate by malate synthase. Disruption of Pta and overexpression of malate dehydrogenase, which converts malate to pyruvate, have the potential to solve glyoxylate accumulation.

(Please, see page 21, line 364-368)

(Fig 7f, 7g, 7h has been renamed to Supplementary Fig. S11a, b, and d, respectively.)

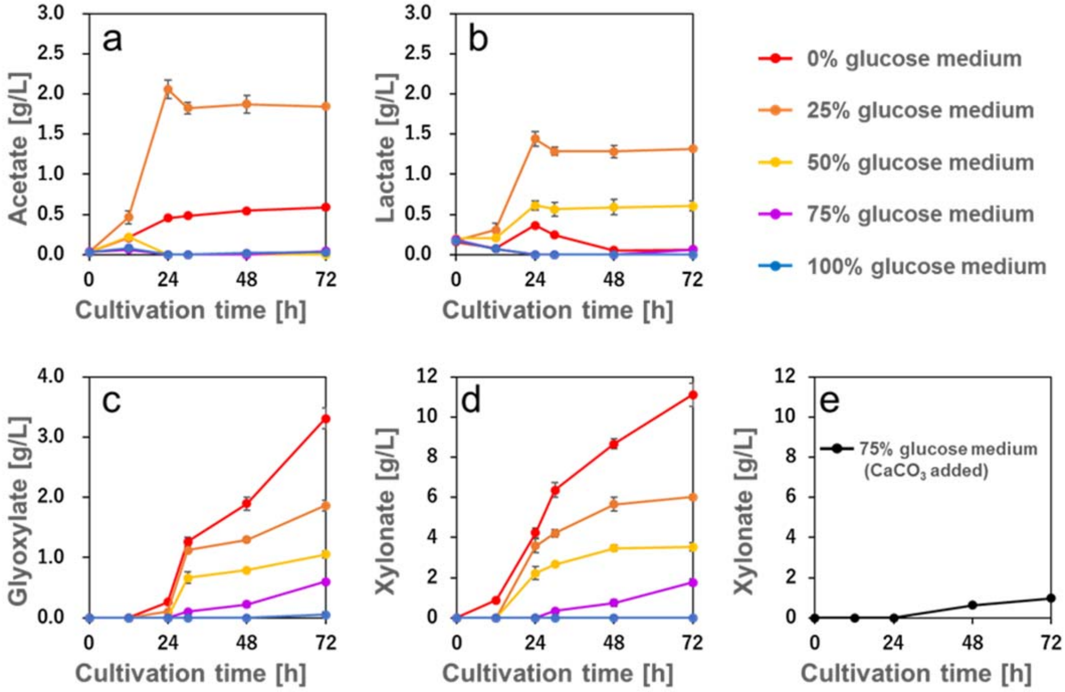

Supplementary Fig. S11. Culture profiles of the GX1xMA strain in M9 minimal medium containing 20-g/L sugar(s) and 5-g/L yeast extract. (a) Acetate accumulation. (b) Lactate accumulation. (c) Glyoxylate accumulation. (d) Xylonate accumulation. (e) Xylonate accumulation in 75% glucose medium with CaCO3 addition condition. Red, orange, yellow, purple, and blue symbols indicate the results of cultivation in 0%, 25%, 50%, 75%, and 100%

glucose medium, respectively, in a–d. The data are presented as the average of three independent experiments, and error bars indicate standard errors.

2. Although it was claimed that the cell growth is responsible to the availability of (controlled by) xylose, Fig.7a does not necessarily show it ? This may be due to the constraint on the flux of the TCA cycle as mentioned above ?

Response

As you pointed out, in the PMPE strains, cell growth is not positively correlated with xylose concentration even though there is enough available xylose. This limitation of cell growth would be due to the constraint on the flux of the TCA cycle. This problem can be solved by eliminating the accumulation of glyoxylic acid and improving the flux of the TCA cycle.

3. It is not clear how the mass balance is satisfied. For example, in Fig.5, 10 g/l of glucose was converted to about 1.6 g/l of MA (10 g/l of xylose was converted to about 4 of OD of biomass at the same time). Please confirm that that the mass balance is satisfied by showing other metabolites. Cell concentration should be given in g/l.

Response

We measured the organic acid accumulation in MA producing strain (GX1xMA and CFT5MA) in M9 minimal medium. These two strains accumulated 1.6 g/L of xylonate, and less than 0.05 g/L of acetate and lactate after 120h cultivation. These results suggested that 44% of consumed xylose was accumulated as xylonate. It is necessary to eliminate the accumulation of xylonate to improve cell growth. The mass balance was calculated based on the carbon-mole of consumed glucose and xylose (Additional Fig. 1, please see the below). As you pointed out, the indication of cell concentration was changed OD_{600} to dry cell weight [g/L]. Therefore, according to your suggestion, we revised our manuscript as follows;

After 120h cultivation of CFT5xMA and GX1xMA, 1.63 ± 0.24 and 1.67 ± 0.20 g/L of xylonate was accumulated, respectively, and the less than 0.05 g/L of acetate and were lactate accumulated in the medium (Supplementary Fig. S9).

(Please, see page 16, line 278-279 and page 17 line 280)

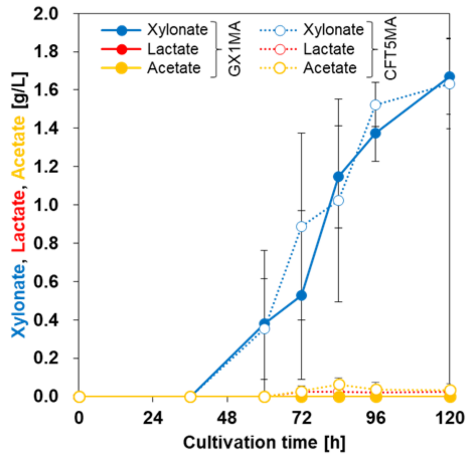

Supplementary Fig. S9. Organic acid accumulation of cis,cis-muconic acid (MA)-producing strains in M9 minimal medium. Black, red, and yellow symbols indicate xylonate, lactate, and acetate concentration, respectively. All open and filled symbols indicate the results for the CFT5xMA and GX1xMA strains, respectively. The data are presented as the average of three independent experiments, and error bars indicate standard errors.

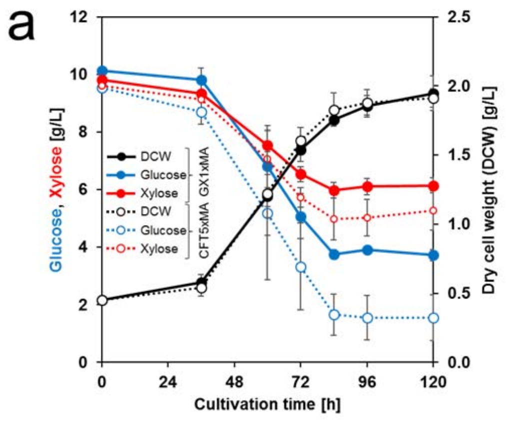

Figure 5a revised (OD_{600} --> DCW)

Cell growth was determined by measuring OD_{600} using a UVmini-1240 spectrophotometer (Shimadzu Corporation, Kyoto, Japan). The dry cell weight was determined based on OD_{600} using a calibration curve (Supplementary Fig. S11).

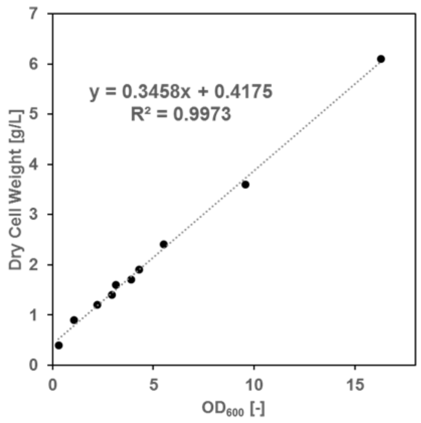

Supplementary Fig. S11. Calibration curve for obtaining DCW from OD.

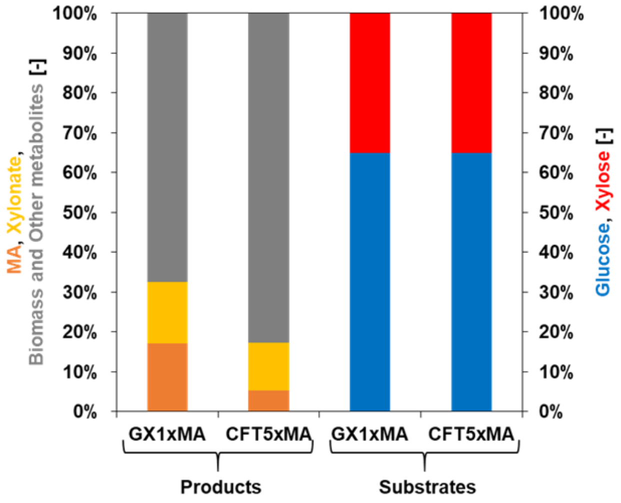

Additional Fig. 1. Mass balance of GX1xMA and CFT5xMA. Orange, yellow, and gray bars indicate the proportion of produced MA, xylonate, and other components including biomass and exhausted CO_2, respectively. Blue and red bars indicate the proportion of consumed glucose and xylose, respectively.

4. The same is true for Figs.7 and 8.

Response

According to your suggestion, we revised our manuscript as follows;

Xylonate, glyoxylate, lactate, and acetate were not accumulated in the

medium after the cultivation of CFT5xTYR or GX1xTYR.

(Please, see page 23, line 401-402)

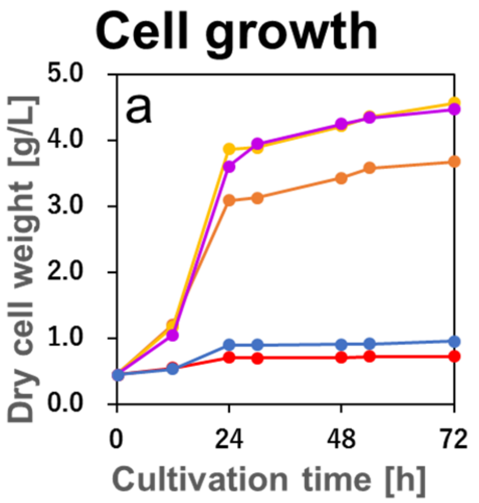

Figure 7a revised (OD_{600} --> DCW)

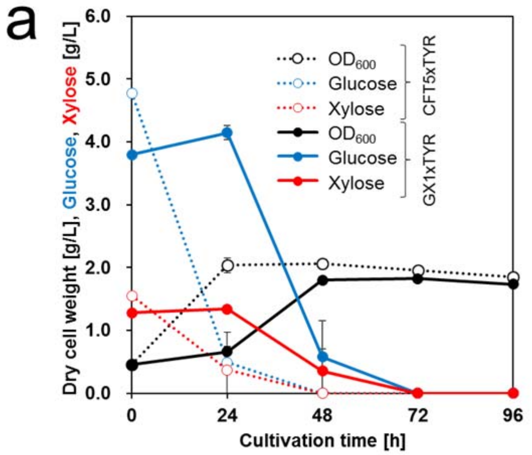

Figure 8a revised (OD_{600} --> DCW)

5. In the pyk mutant strain (together with ppc and pck knockout), PEP may accumulate, which inhibits Pfk activity, activates FBPase, and thus G6P accumulates, and in turn the oxidative PPP may be activated. Then intra-cellular metabolite concentrations should be checked.

6. In addition, the cofactor such as NADPH is necessary for the cell synthesis as well as shikimic acid synthetic pathway, and thus the flux analysis around G6P branch point should be made together with cofactor balance.

Responses of comments 5 and 6

We performed metabolome analysis of constructed strains and control strains (Supplementary Discussions: Metabolome analysis of PMPE strains). As you mentioned, accumulation of F16BP was decreased and PPP was activated in CFT5x and GX1x. Surprisingly, the NADPH levels in CFT5x and GX1x were very low despite PPP being activated. We believe this low NADPH level was caused by overproducing of shikimate pathway derivatives because shikimate dehydrogenase consumes 1 mole of NADPH when synthesis 1 mole of shikimate from 3-dehydroshikimate. We added these results in our manuscript as follows;

The accumulated F16BP in CFT5x and GX1x were significantly lower than ATCC31882. It would have been caused by the accumulation of PEP because PEP inhibits 6-phosphofructokinase 1 (coded by pfkA) which synthesize F16BP from F6P and activates Fructose 1,6-bisphosphatase (coded by fbp) which synthesize F6P from F16BP 1,2. Therefore, PEP accumulation degrades the flux of glycolysis and enhances the flux of pentose phosphate pathway (PPP)[3]. In GX1x, four PPP metabolites, 6PG, Ru5P, Ro5P, and S7P, were significantly increased than ATCC31882x.

(Please, see Supplementary information page 2, line 34-40)

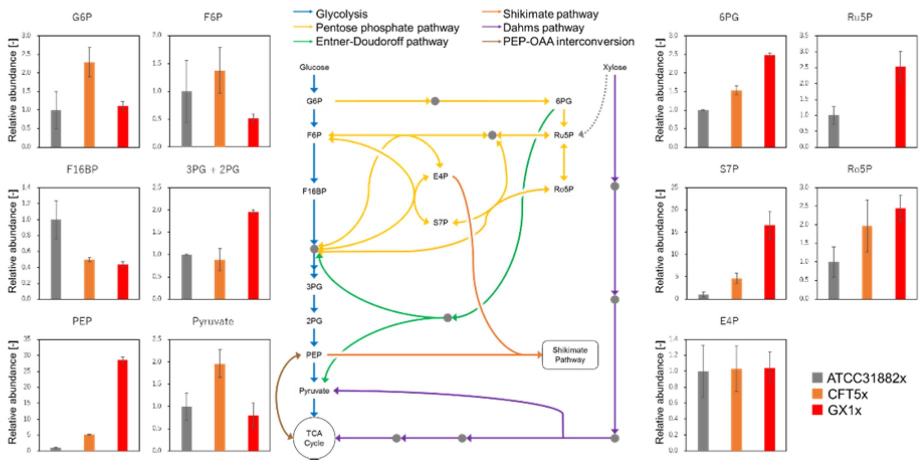

Supplementary Fig. S13. Metabolome analysis of PMPE strains. Figures indicate the specific accumulated amount of metabolites per OD_{600}, used that in ATCC31882x as a benchmark.

In 3PG and 2PG, these were evaluated as a combined value. Gray, orange, and red bars indicate the results of ATCC31882, CFT5x, and GX1, respectively. The data are presented as the average of three independent experiments, and error bars indicate standard deviations.

We also investigated the levels of ATP, ADP, AMP, NADH, NAD^+, NADPH, and NADP+. In GX1x, the ATP level was significantly reduced compared to ATCC31882x and CFT5 (Supplementary Fig. S14a). The NADH level in GX1x was significantly reduced compared to ATCC31882x, and NADH was not detected in CFT5x (Supplementary Fig. S14b). These results show that ATP synthesis by oxidative phosphorylation did not work well due to the restriction of the NADH regeneration in the TCA cycle. Additionally, the NADPH in CFT5x and GX1x were not detected, which caused by the enhanced shikimate pathway because shikimate dehydrogenase consumes 1 mole of NADPH when synthesis 1 mole of shikimate from 3-dehydroshikimate.

(Please, see Supplementary Information page 2, line 40-49)

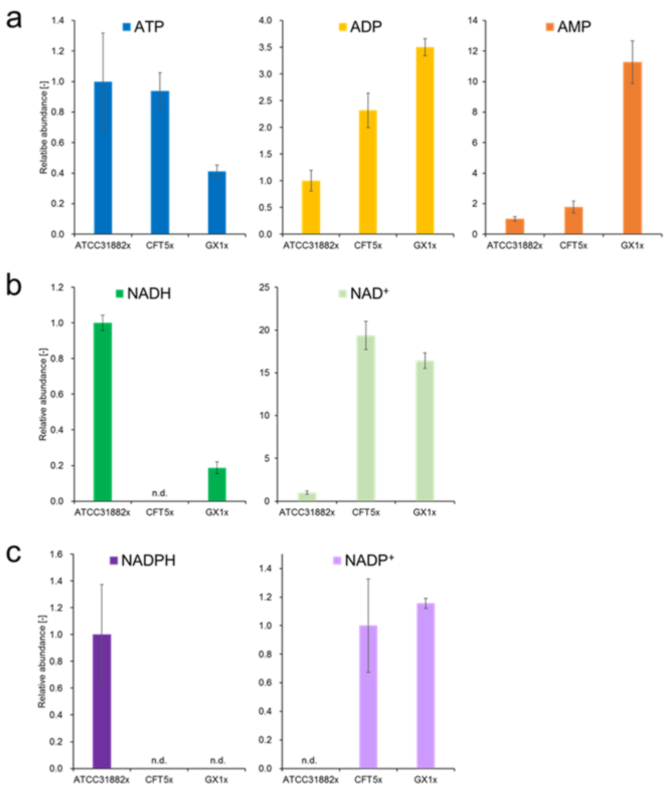

Supplementary Fig. S14. The relative abundance of ATP, ADP, AMP, NADH, NAD^+, NADPH, and $NADP^+$ in PMPE strains. (a) Blue, yellow, and orange bars indicate ATP, ADP, and AMP, respectively. (b) Dark green, and light green bars indicate NADH, and NAD^+, respectively. (c) Dark purple, and light purple bars indicate NADPH, and $NADP^+$, respectively. Figures indicate

the specific accumulated amount of metabolites per OD_{600}. CFT5x was used as a benchmark in $NADP^{+}$ and ATCC31882x was used as a benchmark in other metabolites. n.d., not detected. The data are presented as the average of three independent experiments and error bars indicate standard deviations.

7. Acetate was produced at higher xylose content (75 % and 100 %) as shown in Fig.7f. It should be made clear about the mechanism on how the overflow occurs in relation to the imbalance between the PDH flux and the TCA cycle flux. Pyruvate may also activate Pta (and Pox).

Response

In the low NADH level, the intracellular condition is favorable for the reaction of converting pyruvate to Acetyl-CoA. Furthermore, when glyoxylate is accumulated, Acetyl-CoA and glyoxylate are converted to malate. However, a part of pyruvate was converted to acetate by PoxB or Pta-AckA despite the low NADH level and glyoxylate accumulation, in GX1xMA. As you mentioned, we also believe that Pta was activated in GX1xMA. In the PMPE strain, the accumulation of PEP was significantly increased than ATCC31882, although pyruvate was at the same level as ATCC31882x (Supplementary Fig. S13). According to the previous report, the accumulation of PEP activates acetyl phosphate production by Pta and inhibits the reverse reaction[3]. We revised our manuscript as follows;

It is thought that the accumulation of acetate was caused by the activation of phosphate acetyltransferase (Pta) due to the accumulation of PEP. Pta converts pyruvate to acetyl phosphate and acetyl phosphate is converted to acetate by acetate kinase. PEP acts as a regulator of Pta and activates the production of acetyl phosphate and inhibit the reverse reaction[46].

(Please, see page 20, line 345-350)

8. The lactate formation occurs at higher xylose content (75 % and 50 %) as shown in Fig.7g. This may be caused by the higher redox ratio (NADH/NAD+), and some discussion may be necessary on this. I also wonder if the current culture condition using flask guarantees full aeration? Otherwise, ArcA may repress the TCA cycle activity ?

Response

Since the flask culture is performed in 10 mL medium using the flask with baffles (200 mL working volume) at 220rpm, it is thought to be a complete aerobic condition. The NADH level was low in PMPE strain at log growth phase. (Supplementary discussion: Metabolome analysis of PMPE strains). The NADH level is not favorable for the synthesis of lactate. One possible explanation is that lactate dehydrogenase (LdhA) is activated at low pH[4]. In the flask fermentation, there was a positive correlation between the lactate production and pH (pH 4.59, pH 4.46, pH 4.85, and pH 4.98 in 0%, 25%, 50%, and 75% of glucose medium after 72 h of cultivation, respectively). In the pH control condition (25% glucose), lactate was not detected despite that 0.064 g/L of lactate was accumulated in non-pH-control condition (25% glucose). We revised our manuscript as follows;

The accumulation of lactate might be caused by a decrease in pH. In 50% and 75% of glucose medium, pH was decreased under 5.0 after 72h cultivation. The low pH condition activates lactate dehydrogenase which converts pyruvate to lactate [47].

(Please, see page 20, line 350-351 and page 21 line 352)

9. Since some metabolites such as R5P, E4P, 3PG, and PEP etc. are the precursors of biosynthesis, the arrow from glycolysis and PPP to biomass constituents may be necessary in Fig.1b ?

Response

According to your suggestion, we revised our manuscript as follows;

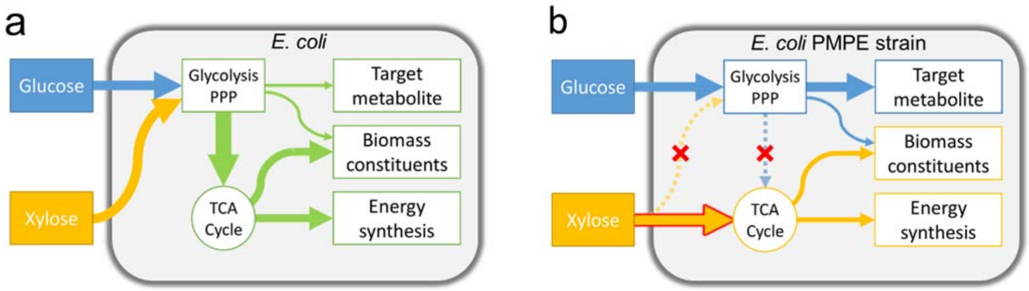

10. The performance of MA production was evaluated in terms of titer and yield. How about productivity ?

Response

According to your suggestion, we revised our manuscript as follows;

The maximum productivity of MA in 25%, 50%, and 75% glucose-containing media were 0.078, 0.072, and 0.114 g/L/h, respectively.

(Please, see page 20 line 335-337)

The maximum productivity of L*-tyrosine in CFT5xTYR and GX1xTYR were 0.026 and 0.021 g/L/h, respectively.*

(Please, see page 23 line 399-401)

11. In Fig.7i, the concentrations of biomass, xylonate, acetate, and lactate etc. should also be shown.

Response

Acetate and lactate were not detected in the medium, and it was difficult to estimate cell weight in the presence of $CaCO_3$ in the medium. According to your suggestion, we revised our manuscript as follows;

(Fig 7f, 7g, 7h has been renamed to Supplementary Fig. S11a, b, and d, respectively.)

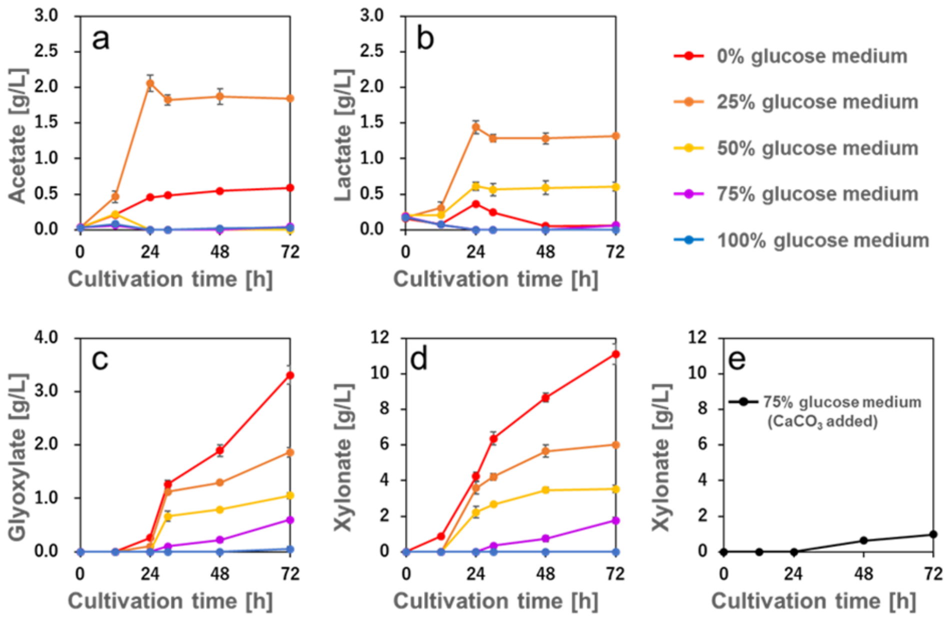

Supplementary Fig. S11. Culture profiles of the GX1xMA strain in M9 minimal medium containing 20-g/L sugar(s) and 5-g/L yeast extract. (a) Acetate accumulation. (b) Lactate

accumulation. (c) Glyoxylate accumulation. (d) Xylonate accumulation. (e) Xylonate accumulation in 75% glucose medium with CaCO3 addition condition. Red, orange, yellow, purple, and blue symbols indicate the results of cultivation in 0%, 25%, 50%, 75%, and 100% glucose medium, respectively, in a–d. The data are presented as the average of three independent experiments, and error bars indicate standard errors.

12. In Fig.8, the concentrations of bi-products should also be shown.

Response

All bi-products (xylonate, glyoxylate, lactate, and acetate) were not detected in the medium. We revised our manuscript as follows;

Xylonate, glyoxylate, lactate, and acetate were not accumulated in the medium after the cultivation of CFT5xTYR or GX1xTYR.

(Please, see page 23 line 401-402)

13. It is better to summarize the data as given in Fig.7 in terms of the specific rates such as the specific growth rate, specific substrate consumption rates, and specific metabolite production rates with respect the xylose content.

Response

As your suggestion, we calculated the specific rates.

Supplementary Table 3. The specific rates of cell growth, substrate consumption, and metabolite production of GX1xMA in each glucose:xylose ratio

Glucose content	specific growth rate [h^{-1}]	specific glucose consumption rate [h^{-1}]	specific xylose consumption rate [h^{-1}]	specific acetate consumption rate [h^{-1}]	specific lactate consumption rate [h^{-1}]
0%	0.067	-	0.026	0.151	0.242
25%	0.108	0.082	0.096	0.127	0.138
50%	0.126	0.081	0.136	-	0.092
75%	0.136	0.046	0.053	-	-
100%	0.119	0.004	-	-	-

14. Although it is decomposed between glycolysis with PPP and TCA cycle, some transcription factors such as Cra (and cAMP-Crp) affects the TCA cycle and glyoxylate pathway gene expression in response to FBP/F1P? Thus, they are indirectly connected ?

Response

As you pointed out, glycolysis with PPP and the TCA cycle are connected indirectly by transcription factors. By the tuning of expression of these transcription factors, it is expected to improve growth and production.

15. The drawback of the present approach is that the cell growth is stopped when either glucose or xylose was used as a single carbon source, where this may happen even when a mixture of multiple carbon sources was used, in which one carbon source was consumed fist, and depleted.

Response

As you mentioned, the depletion of either glucose or xylose causes the stop of cell growth. However, even though the low or high glucose ratio, glucose ratio in consumed sugars was proportional to the glucose ratio of supplemented substrates sugars at the starting condition in the medium (Fig. 7c, 7d, 8a, and Additional Fig. 2, please see the below.). Improving sugar uptake and metabolisms of these sugars are future challenges to further increase in production.

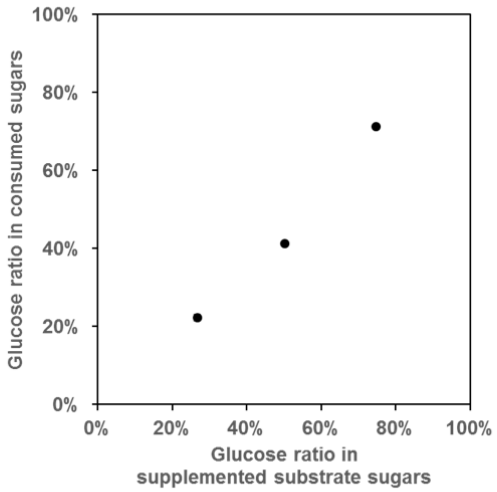

Additional Fig. 2. The plot of glucose ratio of consumed sugar and glucose ratio of supplemented substrate sugar.

Although the proposed approach is attractive from the application point of view, basic question may arise as to the metabolic regulation as compared to the wild-type stain. Namely, as the cell growth rate increases, the metabolism changes from full respiration (oxidative phosphorylation) to respiro-fermentative metabolism with increasing the glycolytic flux to enhance the substrate-level phoshorylation in the wild-type strain. This may be due to oxidative stress regulation. Thus, the glycolysis with PPP must be tightly connected from such point of view, and I wonder if the current stain can endure long-term fermentation without such mechanism ?

Response

The problem of metabolic regulation including energy synthesis is a challenge to be addressed. In the present stage, the PMPE strain cannot grow well under respiro-fermentative or fermentative metabolism (Fig. 8c, Supplementary Fig. S16). We believe that improving sugar uptake will have as much priority as engineering of metabolic regulators, because glucose/xylose was not consumed completely (Fig. 5 and Fig. 7). To overcome this problem, it is necessary to investigate the expression level of global regulatory proteins and other factors influence metabolism in PMPE strains.

Responses to the comments by reviewer #3

Thank you for your thoughtful comments that have helped us to improve our manuscript considerably. We revised our manuscript according your suggestion as follows.

Reviewer #3 (Remarks to the Author):

The authors have developed an interesting and novel approach for biochemical production that they term "parallel metabolic pathway engineering" (PMPE). Via PMPE, glucose is mainly for target chemical production and xylose for supplying essential metabolites for cell growth via an introduced Dahms pathway. To develop this methods, they have performed a systematic characterization of carbon flow through central metabolism in different backgrounds, ultimately landing on the set of mutations required to partition carbon from two different sources. 13C labeling studies are performed to prove their hypotheses in this regard. The approach could be applicable to the production of other chemicals, which would therefore make it significant. This is a well-planned and executed study, and the article is well-written. There are a shortcomings, however, and points in need of clarficiation.

1. The authors chose to demonstrate their method via the production of 2 shikimate pathway derived products. While convenient, this doesn't really do much to demonstrate 'versatility' which was their objective. They fact that PMPE improved tyrosine production after it was shown to improve MA production was not surprising at all. While useful to test, this doesn't satisfy the objective. And it is an important objective. One Line 107, among central carbon intermediates, PEP was considered the most important intermediate. Why would this be so? Would this not depend on what one is trying to produce? If the authors truly want to demonstrate the versatility of their method, they should really apply it to the production of another non-shikimate and non-PEP derived compound. The discuss 1,2-propanediol as a possible candidate. If PMPE is truly to be presented as a versatile and important 'strategy' for metabolic engineering, it is not sufficient to simply save this for future, in my opinion.

Response

As you pointed out, we applied the PMPE strategy in 1,2-propanediol production. The strain GX1xPD was constructed by introduced 1,2-propanediol synthesis pathway into the strain GX1x. GX1xPD produced 0.53 ± 0.01 g/L of 1,2-propanediol, which was 1.40-fold higher than the control strain. The production yield reached 0.53 ± 0.16

g/g-glucose, 11.4-fold higher than the control strain. Therefore, according to your suggestion, we added the results and discussions in our manuscript as follows;

Additionally, we constructed the strain producing 1,2-propanediol as a non-shikimate pathway chemical derived from GX1x (GX1xPD). GX1xPD increased the production titer and the yield of 1,2-propanediol by 1.40- and 11.4-fold, respectively, compared to the control strain. (Supplementary Discussion: 1,2-Propanediol production in the PMPE strain). These results suggested that PMPE is versatile for applying the production of various compounds including non-shikimate pathway derivatives.

(Please, see page 25 line 426-432)

1,2-Propanediol production in the PMPE strain

To confirm the versatility of PMPE in non-shikimate pathway, we attempted to produce 1,2-propanediol using the PMPE strain (Supplementary Fig. S15). The GX1xPD strain, a GX1-derived strain harboring Bs_mgsA, gldA, and fucO, encoding methylglyoxal synthase, glycerol dehydrogenase, and 1,2-propanediol oxidoreductase, respectively, was cultivated in M9Y medium containing both 20 g/L of glucose and 5 g/L of xylose in micro-aerobic condition at 30°C. GX1xPD strain produced 0.53 ± 0.01 g/L of 1,2-propanediol after 96 h of cultivation, representing a 1.40-fold increase compared with that produced by the control strain CTR2PD (Supplementary Fig. S16). The maximum productivity of 1,2-propanediol in CTR2PD and GX1xPD were 0.010 and 0.015 g/L/h, respectively. The production yield reached 0.53 ± 0.16 g/g-glucose after 96 h of cultivation, reflecting a 11.4-fold increase compared with that using the CTR2PD strain and corresponding to 62% of the theoretical maximum without considering cell growth (0.84 g/g of glucose)4. Jain et al. engineered the metabolism in E. coli for production of 1,2-propanediol. They disrupted tpiA which is coding triosephosphate isomerase to increase the production5. The final strain in this study produced 1.2 g/L of 1,2-propanediol from 20 g/L of glucose. However, tpiA-deficient strains can only produce up to 1 mole of 1,2-propanediol from 1 mole of glucose, thus the theoretical maximum yield is 0.42 g/g. In GX1xPD, the yield was higher than the theoretical maximum yield in tpiA-deficient strains, it suggests that the PMPE strategy has the potential to dramatically improve the yield of 1,2-propanediol from glucose. For further improvement of 1,2-propanediol production, the optimization of metabolism is necessary because GX1 was engineered to increase the production of shikimate pathway derivatives. In particular, improving NADH regeneration is necessary for 1,2-propanediol production in the PMPE strain. 2 moles of NADH was required when 1 mole of 1,2-propanediol is synthesized (Supplementary Fig. S15). The NADH level in the PMPE strain was very low (Supplementary Fig. S14b). By improving the redox levels of NADH / NAD^+, further increase in the production of 1,2-propanediol

can be expected.

(Please, see Supplementary Information page 3 line 51-74 and page 4 line 75-79)

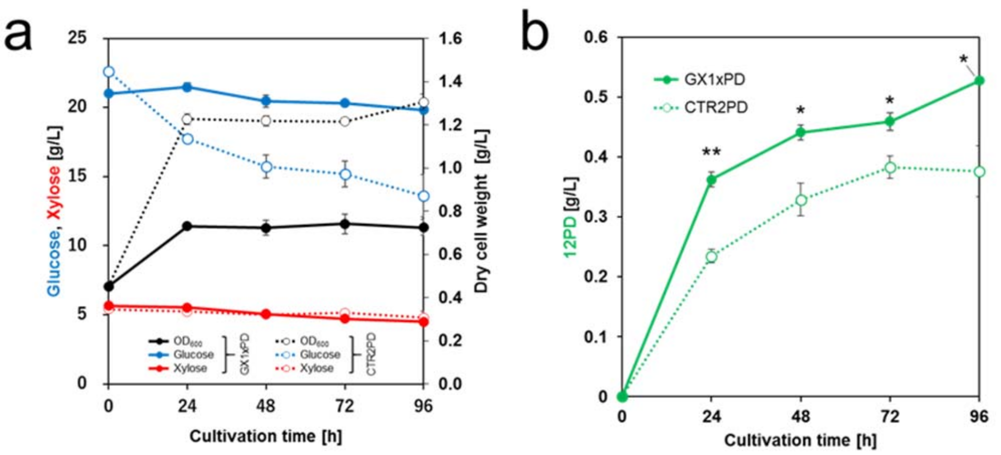

Supplementary Fig. S16. Culture profiles of 1,2-propanediol-producing strains in M9 minimal medium. (a) Black, blue, and red symbols indicate bacterial cell growth, glucose consumption, and xylose consumption, respectively. (b) Green symbols indicate the produced amounts of 1,2-propanediol. All open and filled symbols indicate the results for the CTR2PD and GX1xPD strains, respectively. The data are presented as the average of three independent experiments, and error bars indicate standard errors. P values were computed using the two-tailed Student's t-test (, $P < 0.05$; **$P < 0.01$).*

2. The choice of shikimate pathway derived compounds for demonstrating PMPE is a bit peculiar to begin with, since it is well known that the yields/titers of such products is much higher from xylose (due to higher levels of PEP and E4P). That is, using xylose to produce such compounds and glucose for growth would perhaps be the optimal strategy. Of course, that would require an entirely different set of parallel pathways. However, it does beg that question: what are the optimal product targets of this strategy? This requires some thinking and discussion, since it currently seems as though the products were selected just based on convenience.

Response

As you mentioned, xylose is more suited to produce the shikimate pathway derivatives in wild type and or metabolic modified strain. However, PMPE using glucose as the substrate for products of interest has the potential to produce various compounds

derived from not only the shikimate pathway but also glycolysis pathway and PPP, such as 1,2-propanediol. In addition, there is a larger amount of glucose than xylose in our environment, and it would have industrially advantage to use glucose to produce a variety of useful chemicals. We revised our manuscript as follows;

Since there is a larger amount of glucose than xylose in nature, we selected glucose as a substrate for producing target chemicals, and xylose is used in the pathway that synthesizes essential metabolites.

(Please, see page 9 line 145-147)

3. Fig 2: Panel C is not essential, as it contains the same data as in B.

Response

According to your suggestion, we revised our manuscript as follows;

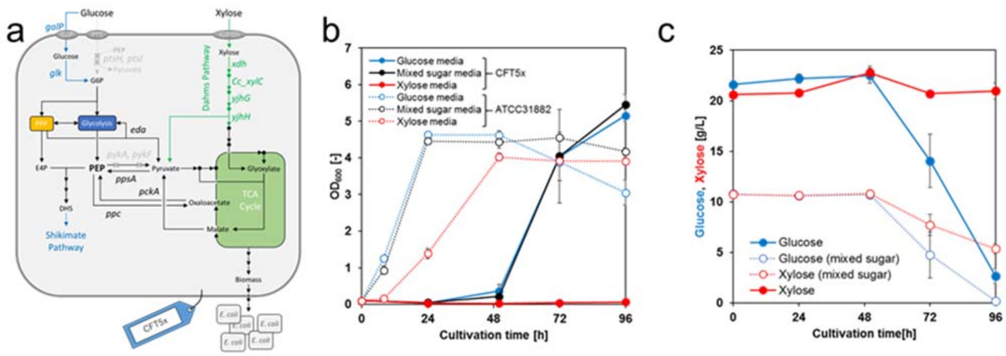

*Fig. 2. Strain construction and cell growth examination. (**a**) Metabolic design of CFT5x. (**b**) Bacterial cell growth. Blue, black, and red symbols indicate the growth of the CFT5x strain and ATCC31882 cultured in medium supplemented with glucose, a glucose–xylose mixture, and xylose, respectively. (**c**) Glucose and xylose consumption. Filled blue symbols indicate glucose consumption in glucose-containing medium. Filled red symbols indicate xylose consumption in xylose-containing medium. Open blue and open red symbols indicate glucose and xylose consumptions in mixed sugar-containing medium, respectively. G6P, glucose-6-phosphate; PEP, phosphoenolpyruvate; E4P, erythrose 4-phosphate; DHS, dehydroshikimate; and Gluconate-6P, gluconate-6-phosphate. The data are presented as the average of three independent experiments, and error bars indicate standard errors.*

4. In CFT5x, xylose was not fully consumed in 96 h. Was it ever? How to improve in this strain?

Response

In revised Fig. 2c (Fig. 2d in the original version), The starting time of consuming glucose and xylose was the same, but the consumption rate of xylose was lower than glucose. CFT5x would need further time to consume xylose completely. In PMPE strains, accumulation of metabolites derived from xylose such as xylonate is one of the problems to solve (Supplementary Fig. S9a, Supplementary Fig. S11). Pulling the carbon flow from glycolaldehyde to glyoxylate by overexpressing lactaldehyde dehydrogenase and glycolate oxidase is one possible strategy to improve the cell growth. Therefore, according to your suggestion, we revised our manuscript as follows.

To reduce the accumulation of xylonate, overexpressing lactaldehyde dehydrogenase (AldA) would be one of the possible strategies. Glycolaldehyde synthesized via Dahms pathway is converted to glycolate by AldA and then glycolate is converted to glyoxylate by glycolate oxidase. Cabulong et al. increased glycolic acid production by overexpressing AldA[33]. In PMPE strains, it might be possible to improve the accumulation of xylonate that pulling the carbon flow from glycolaldehyde to glyoxylate by overexpressing AldA and glycolate oxidase. Another explanation of the glyoxylate accumulation is due to the shortage of acetyl-CoA. Glyoxylate and acetyl-CoA are converted to malate by malate synthase. Disruption of Pta and overexpression of malate dehydrogenase, which converts malate to pyruvate, have the potential to solve glyoxylate accumulation.

(Please, see page 21 line 358-368)

5. The observation that GX1x did not grow in M9 minimal media with just xylose was surprising. We routine grow ATCC31882 delta(pykAF) in xylose minimal media. It seems surprising that the additional mutations would inhibit this. Any thoughts on this? In the absence of glucose, native xylose metabolism should be quite active. This likely requires some additional discussion. Also, was this truly minimal media or was yeast extract added? It seems that this strain also carries auxotrophic mutations. Please be clear about this point throughout the paper.

Response

We investigated the culture profiles of PMPE strains in M9 minimal medium containing xylose as a sole carbon source with amino acids supplements. Parental strain ATCC31882 is auxotrophic for Tyr/Trp, and the strains CFT5 derivatives is additionally required Phe. Overexpressing XylAB (and Dahms pathway enzymes) in GX1

and CFT5 derivative strains did not improve the growth (See Supplementary Fig. S6 shown in below). All medium used in this study were supplemented with proper aromatic amino acids. We added these results in our manuscript as follows;

It was thought that the GX1x strain did not grow in M9 minimal medium containing only xylose because of the insufficient activity of the endogenous xylose catabolic pathway (i.e., xylAB). We constructed the strain overexpressing XylA and XylB derived from GX1x. Contrary to expectations, this strain did not glow in M9 minimal medium with a glucose–xylose mixture as the carbon source. Furthermore, the strains derived from CFT5 overexpressing Dahms pathway enzymes (Xdh, CcxylC, YjhH, and YjhG) and/or enzymes of the endogenous xylose catabolic pathway (XylA and XylB) also did not grow in the same condition (Supplementary Fig. S6).

(Please, see page 15 line 251-258)

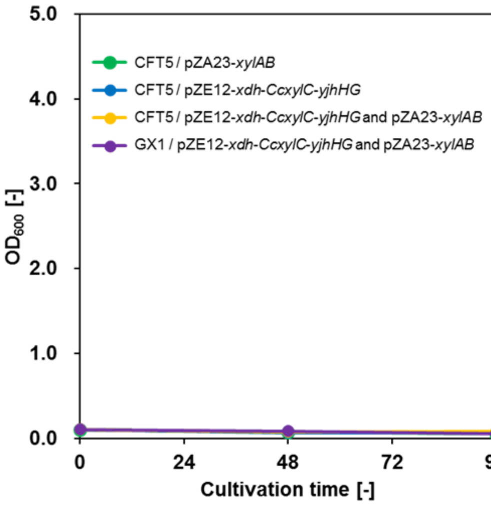

Supplementary Fig. S6. The effect of overexpressing XylA and XylB on the cell growth in M9 minimal medium with glucose and xylose as the carbon source. Green, blue, and yellow symbols indicate the bacterial cell growth of CFT5 harboring pZA23-xylAB, CFT5 harboring pZE12-xdh-CcxylC-yjhHG, and CFT5 harboring pZE12-xdh-CcxylC-yjhHG and pZA23-xylAB. Purple symbols indicate the bacterial cell growth of GX1 harboring pZE12-xdh-CcxylC-yjhHG and pZA23-xylAB.

M9 minimal medium comprised the following (per liter): carbon source (20-g glucose, 10-g glucose, and 10- or 20-g xylose), 0.5-g NaCl, 17.1-g

Na_2HPO_4•$12H_2O$, 3-g KH_2PO_4, 1-g NH_4Cl, 246-mg $MgSO_4$•$7H_2O$, 14.7-mg $CaCl_2$•$2H_2O$, 2.78-mg $FeSO_4$•$7H_2O$, 10-mg thiamine hydrochloride, 40-mg L-tyrosine, 40-mg L-tryptophan, and 100-mg L-phenylalanine (Tyr and Trp were included because ATCC31882 is auxotrophic for these amino acids and the CFT5 derivative strains are auxotrophic for Phe).

(Please, see Supplementary Information page 5 line 91-94)

References

1. Wei Niu, Mapitso N. Molefe, and & Frost*, J. W. Microbial Synthesis of the Energetic Material Precursor 1,2,4-Butanetriol. (2003). doi:10.1021/JA036391+
2. Liu, H., Valdehuesa, K. N. G., Nisola, G. M., Ramos, K. R. M. & Chung, W. J. High yield production of d-xylonic acid from d-xylose using engineered *Escherichia coli*. *Bioresour. Technol.* **115**, 244–248 (2012).
3. Campos-Bermudez, V. A., Bologna, F. P., Andreo, C. S. & Drincovich, M. F. Functional dissection of *Escherichia coli* phosphotransacetylase structural domains and analysis of key compounds involved in activity regulation. *FEBS J.* **277**, 1957–1966 (2010).
4. Mat-Jan, F., Alam, K. Y. & Clark, D. P. Mutants of *Escherichia coli* deficient in the fermentative lactate dehydrogenase. *J. Bacteriol.* **171**, 342–8 (1989).

Reviewers' comments:

Reviewer #1 (Remarks to the Author):

The correction has been made or discussed in the revised manuscript. However, some points are still unclear.

1. CFT5 overexpressing xylAB or Dahms pathway enzymes did not grow. Please discuss why?

2. "Authors need to incorporate the data with wild type strain in Fig 2, 4 and 5. This result will improve the discussion with respect to indigenous xylose utilization capacity of E. coli". The data of control strain were added in the revised manuscript; however, it was not discussed anywhere in the manuscript. The late lag phase and slower carbon utilization of engineered strain compared with control strain can also be discussed, especially by mentioning the data of supplementary Fig S14.

3. The CFT5 stains can grow after a very long lag phase, about two days (Supplementary Fig. S2b). The reviewer believe that there is a big change in gene expression pattern. Do you have any data that can explain this phenomenon?

4. In Figure 8, CFT5xTYRv showed high titer, but less yield. From this result, it may be hard to mention that PMPE is a promising strategy to improve production even in micro-aerobic conditions.

5. The reviewer wonder if xylose could be utilized through the isomerase route in the GX1 strain because authors only mentioned that GX2 strains lack activity of genes associated with the isomerase pathway.

Reviewer #2 (Remarks to the Author):

This is the modified version of the original manuscript, and it is significantly improved based on the reviewers' comments. Some further clarification and improvement may be necessary before publication.

1. English should be thoroughly checked by the native speaker. In particular, it must be careful about the grammar of the revised portion in pages 10, 12, 13, 17, and 26.
2. In page 22, it is stated 'Disruption of Pta and overexpression of malate dehydrogenase, which converts malate to pyruvate have… ' It may be malic enzyme (Mez) which converts malate to pyruvate ?
3. In page 23, it is stated that CCR was almost reversed. This may be relaxed ?
4. In page 24, lines 412-418, it is stated that GX1xTYR is better than CFT5xTYR in terms of production yield under micro-aerobic condition. However, the substrate consumption rates of GX1xTYR are very low, and not attractive in practice. I wonder why the glucose consumption was significantly low in GX1xTYR, while glucose was rapidly consumed (and fermentation products such as lactate, formate, ethanol and acetate may have been produced ?) in CFT5xTYR. Under microaerobic condition, TCA cycle is repressed by ArcA, and thus xylose may be not be essentially consumed, which seems to be one of the the drawbacks ?

Reviewer #3:

[Editor: Reviewer #3 states in Remark to Editor section that (s)he is satisfied with the revision.]

Responses to the comments by reviewer #1

Thank you for your thoughtful comments that have helped us to improve our manuscript considerably. We revised our manuscript according your suggestion as follows.

1. CFT5 overexpressing xylAB or Dahms pathway enzymes did not grow. Please discuss why?

Response

In CFT5, expression of genes in the gluconeogenesis (*maeB*, *pckA*, and *ppsA*) was enhanced and expression of *gltA* and *ppc* which code enzymes supplying the intermediate of the TCA cycle was suppressed (Supplementary Fig. S2d). Pyruvate and glyoxylate supplied via Dahms pathway would have been converted to PEP in glycolysis and could not be used in the TCA cycle. According to your suggestion, we revised our manuscript as follows;

In this strain, while pyruvate and glyoxylate were supplied via Dahms pathway, these metabolites would have been converted to intermediates of glycolysis and could not be used in the TCA cycle.

(Please, see page 15, line 57-59)

2. "Authors need to incorporate the data with wild type strain in Fig 2, 4 and 5. This result will improve the discussion with respect to indigenous xylose utilization capacity of E. coli".
The data of control strain were added in the revised manuscript; however, it was not discussed anywhere in the manuscript. The late lag phase and slower carbon utilization of engineered strain compared with control strain can also be discussed, especially by mentioning the data of supplementary Fig S14.

Response

By the transcriptome analysis, it was suggested that the expression of genes in central metabolism had changed in CFT5 and GX1x. It would have caused the defect of regeneration of NADH via the TCA cycle and the shortage of ATP in CFT5 and resulted in the long lag phase and slow cell growth. According to your suggestion, we revised our manuscript as follows;

In GX1x, the ATP level was significantly reduced compared to ATCC31882x and CFT5 (Supplementary Fig. S15a). In CFT5 and GX1, GalP/Glk system consumes 1 mole of ATP when converting 1 mole of glucose to G6P. Furthermore, pyruvate kinases (coded by pykA and pykF) which synthesize pyruvate and ATP from PEP were disrupted in CFT5 and GX1. These metabolic modifications would have caused the lower levels of ATP and the late and lower cell growth (Fig. 2b, 4b, 5a). The NADH level in GX1x was significantly reduced compared to ATCC31882x, and NADH was not detected in CFT5x (Supplementary Fig. S15b). These results are consistent with the transcriptome analysis suggesting the carbon flux suppression of the TCA cycle in CFT5 (Supplementary Fig. 2d) and show that ATP synthesis by oxidative phosphorylation did not work well due to the restriction of the NADH regeneration in the TCA cycle.

(Fig S14 was renamed Fig S15)

(Please, see Supporting Information page 4 line 74-85)

3. The CFT5 stains can grow after a very long lag phase, about two days (Supplementary Fig. S2b). The reviewer believe that there is a big change in gene expression pattern. Do you have any data that can explain this phenomenon?

Response

According to your suggestion, we examined the gene expression patterns of CFT5. In CFT5, the expression of some genes related with gluconeogenesis was enhanced. On the other hand, the expression of genes in TCA cycle and anaplerosis was suppressed. These results suggest that the low carbon flux into the TCA cycle caused the long lag phase and slow cell growth in CFT5. We added the results as follows;

The gene expression patterns of these strains were also evaluated. (Supplementary Discussion: Characteristics of central metabolic genes expression in CFT5 strain).

(Please, see page 10, line 158-161)

Characteristics of central metabolic genes expression in CFT5 strain

In the M9M medium culture of CFT5, lag phase was very long, about two days (Supplementary Fig. S2b). It is thought that there are differences in expression of genes in central metabolism from ATCC31882, the parental strain which can grow rapidly in M9 minimal medium. To examine the characteristics of gene expression in CFT5, we measured genes transcription by real time revers transcription PCR. ATCC31882 and CFT5 were cultured in M9 minimal medium containing 20 g/L glucose supplemented with 10 mM sodium malate. The initial optical density at

600 nm (OD_{600}) of the cultures was 1.0, and cells were collected 1h (ATCC31882, lag phase), or 22h (CFT5, lag phase) and 66h (CFT5, early logarithmic growth phase) for real time RT-PCR analysis. An increase or decrease compared with ATCC31882 was measured in seven genes, maeB (coding malic acid enzyme), ppsA (coding PEP synthase), ppc (coding PEP carboxylase), pckA (coding PEP carboxykinase), pgi (coding G6P isomerase), zwf (coding G6P dehydrogenase), gltA (coding citrate synthase) (Supplementary Fig. S2c). Supplementary Fig. S2d shows the results of relative expression levels of mRNA in each gene. In the lag phase, CFT5 increased the expression of maeB, ppsA, pckA. These three genes code enzymes of gluconeogenesis which is the reverse metabolism of glucose catabolism. Expression of gltA was decreased in the lag phase of CFT5. Since citrate synthase catalyzes the first reaction of the TCA cycle, it is thought that the TCA cycle would have been suppressed in CFT5. Furthermore, expression of ppc was also decreased. PEP carboxylase is the enzymes of anaplerosis which is the metabolic reaction supplying oxaloacetate, an intermediate of the TCA cycle. These results suggested that the carbon flow to the TCA cycle reduced. The expression of pgi and zwf which code the enzymes catalyzing the first reaction of glycolysis and pentose phosphate pathway, respectively, were enhanced in the lag phase of CFT5. In the early logarithmic growth phase of CFT5, all genes were increased when compared with the lag phase of CFT5, but there was no change in the increase / decrease trend when compared with ATCC31882. Taken together, it is thought that sugar catabolism was promoted in the upstream of the central metabolism, gluconeogenesis was enhanced and the TCA cycle was suppressed, which might have caused the long lag phase and the slow cell growth in CFT5.

(Please, see Supporting Information page 1, line 16-24 and page 2)

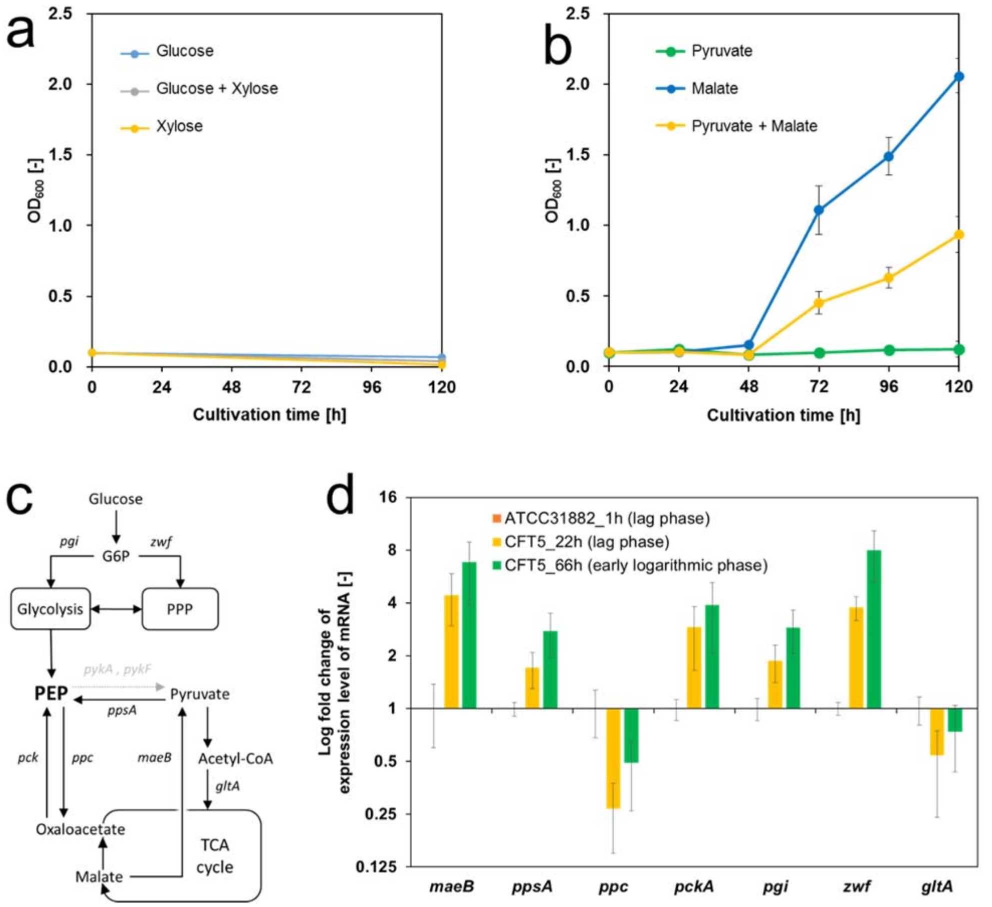

Supplementary Fig. S2. Growth of the CFT5 strain. (a) Blue, gray, and yellow symbols indicate M9 minimal medium supplemented with glucose, a glucose–xylose mixture, and xylose, respectively. (b) Green, blue, and yellow symbols indicate the use of minimal medium containing glucose supplemented with 10-mM sodium pyruvate, 10-mM sodium malate, and mixture of 5-mM pyruvate and 5-mM malate, respectively. (c) Diagram of central metabolism in CFT5. (d) Transcriptome analysis of CFT5. Yellow and green bars indicate log fold change of expression level of mRNA from ATCC31882 (after 1h cultivation) in the lag phase of CFT5 (after 22h cultivation) and in the early logarithmic phase of CFT5 (after 66h cultivation), respectively. The data are presented as the average of three independent experiments, and error bars indicate standard errors.

4. In Figure 8, CFT5xTYR showed high titer, but less yield. From this result, it may be hard to mention that PMPE is a promising strategy to improve production even in micro-aerobic

conditions.

Response

According to your suggestion, we revised our manuscript as follows;

These results suggested that PMPE is superior also in micro-aerobic conditions at the production yield, however, the production titer needs to be improved.

(Please, see page 24 line 412-414)

5. The reviewer wonder if xylose could be utilized through the isomerase route in the GX1 strain because authors only mentioned that GX2 strains lack activity of genes associated with the isomerase pathway.

Response

As you pointed out, GX1 has an endogenous xylose isomerase pathway and we thought the possibility that xylose catabolized via isomerase pathway. To confirmed this, we compared GX1x and GX2x (disrupted the isomerase pathway in GX1x) by metabolome analysis, and the profiles of GX1x were almost the same as the GX2x. These results suggests that the isomerase pathway did not work enough in GX1x and almost all xylose was consumed via Dahms pathway. We revised our manuscript as follows;

To prevent accidental activation of the endogenous xylose isomerase pathway, we generated the GX2xMA strain, a GX1xMA-derived strain in which xylA (xylose isomerase) and xylB (xylulokinase) were disrupted. ^{13}C-metabolic analysis of the GX2xMA strain revealed profiles similar to those of the GX1xMA strain (Fig. 6). This result suggested that the metabolites derived from the intermediates of glycolysis or PPP were not sufficiently synthesized from consumed xylose in the strain in which the Dahms pathway was introduced.

(Please, see page 18, line 304-311)

Responses to the comments by reviewer #2

Thank you for your thoughtful comments that have helped us to improve our manuscript considerably. We revised our manuscript according your suggestion as follows.

1. English should be thoroughly checked by the native speaker. In particular, it must be careful about the grammar of the revised portion in pages 10, 12, 13, 17, and 26.

Response

According to your suggestion, we revised our manuscript as follows;

However, Xdh has no or little enzymatic activity for glucose or glucose-6-phosphate as substrates[32].
(Please, see page 12, line 190-191)

It is thought that the balance of PEP–OAA interconversion may have been altered by the disruption of the ED pathway.
(Please, see page 13, line 208-209)

As a control of glucose and xylose co-utilizing strain, strain CTR2 was constructed by disrupting ptsG and pheA from ATCC31882. These genes are responsible for catabolite repression (ptsG) and production of L-phenylalanine competing with MA production (pheA), respectively.
(Please, see page 16, line 274-278)

Using Weimberg pathway is an alternative option because this pathway supplies α-ketoglutarate from xylose without producing glyoxylate.
(Please, see page 26, line 453-454)

2. In page 22, it is stated ‘Disruption of Pta and overexpression of malate dehydrogenase, which converts malate to pyruvate have… ‘. It may be malic enzyme (Mez) which converts malate to pyruvate?

Response

In this sentence, “malate dehydrogenases” means the malic enzymes coded by

maeB (NADP-dependent malic enzyme) or *sfcA* (NAD-dependent malic enzyme), which converts malate to pyruvate. We revised our manuscript as follows;

Disruption of Pta and overexpression of malic enzymes coded by maeB (NADP-dependent malic enzyme) or sfcA (NAD-dependent malic enzyme), which converts malate to pyruvate, have the potential to solve glyoxylate accumulation.
(Please, see page 21, line 368-369 and page 22, line 370-371)

3. In page 23, it is stated that CCR was almost reversed. This may be relaxed?

Response

As you pointed out, we revised our manuscript as follow;

We demonstrated MA production using the PMPE strain. Figures 5 and 7f show that CCR was almost relaxed, and glucose and xylose consumptions were simultaneously achieved.
(Please, see page 22, line 384-385)

4. In page 24, lines 412-418, it is stated that GX1xTYR is better than CFT5xTYR in terms of production yield under micro-aerobic condition. However, the substrate consumption rates of GX1xTYR are very low, and not attractive in practice. I wonder why the glucose consumption was significantly low in GX1xTYR, while glucose was rapidly consumed (and fermentation products such as lactate, formate, ethanol and acetate may have been produced?) in CFT5xTYR. Under microaerobic condition, TCA cycle is repressed by ArcA, and thus xylose maybe not be essentially consumed, which seems to be one of the drawbacks?

Response

According to your suggestion, we measured the by-products (formate, lactate, acetate, xylonate, and ethanol) accumulation in micro-aerobic conditions. Some organic acids and ethanol were accumulated and the by-products production in CFT5xTYR was higher than GX1xTYR (Supplementary Fig. S12a; see the below). We also evaluated *arcA* transcription level by RT-PCR. In micro-aerobic condition, *arcA* expression was increased in CFT5xTYR and GX1xTYR compared to aerobic condition (Supplementary Fig. S12b). As you pointed out, the down-regulation of the TCA cycle by ArcA is not

desirable for the efficient use of xylose in the PMPE. We revised our manuscript as follows;

In micro-aerobic condition, CFT5xTYR and GX1xTYR produced by-products (such as organic acids and ethanol) and the expression level of aerobic respiration control protein (ArcA, coded by arcA) was increased in micro-aerobic conditions (Supplementary Fig. S12a, S12b). ArcA down-regulates the transcription of genes in the TCA cycle [52]. To improve the productivity of PMPE strain in micro-aerobic conditions, the engineering of metabolic regulators including ArcA would be necessary.

(Please, see page 24, line 414-420)

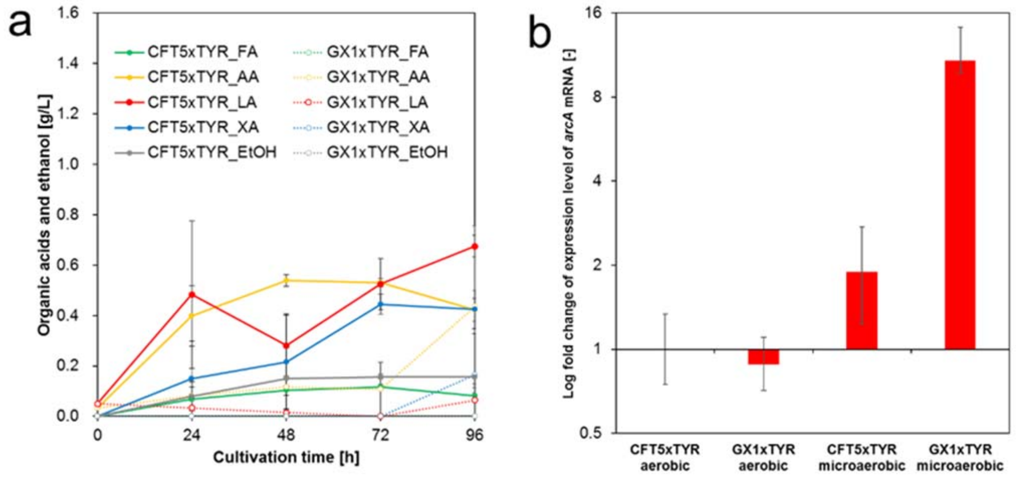

Supplementary Fig. S12. Culture profile of CFT5xTYR and GX1xTYR. (A) By-products production in anaerobic condition. All filled symbols and all open symbols indicate results of CFT5xTYR and GX1xTYR, respectively. FA, formate; AA, acetate; LA, lactate; XA, xylonate; and EtOH, ethanol. (B) Relative transcriptional levels of arcA. The arcA mRNA level of CFT5xTYR in aerobic condition was used as a benchmark. The data are presented as the average of three independent experiments, and error bars indicate standard errors.

REVIEWERS' COMMENTS:

Reviewer #1 (Remarks to the Author):

The authors have made all the correction in the revised manuscript. The manuscript is ready for the acceptance after few minor comments.

Minor comments
1. Pg. 26, line 456, 'Xylonate dehydrogenase in the Dahms pathway is a rate-limiting enzyme'. I think It is 'Xylonate dehydratase' which is a rate limiting enzyme for Dahms pathway.
2. Pg. 27 line 467, 'significantly contribute to the production of other chemicals with high yields'. Please change this to 'significantly contribute to the production of other non-shikimate pathway chemicals with high yields'.
3. Pg. 27 line 460, Using Weimberg pathway….glyoxylate. Support this sentence with reference.

4. Pg 23, line 399, Please rewrite the subsection or separate it in two, one for tyrosine and other one for 1,2-propanediol.

Reviewer #2 (Remarks to the Author):

The manuscript has been appropriately modified, and this may be now accepted.

Responses to the comments by reviewer #1

Thank you for your thoughtful comments that have helped us to improve our manuscript considerably. We revised our manuscript according your suggestion as follows.

Minor comments

1. Pg. 26, line 456, 'Xylonate dehydrogenase in the Dahms pathway is a rate-limiting enzyme'. I think It is 'Xylonate dehydratase' which is a rate limiting enzyme for Dahms pathway.

Response

As you pointed out, we revised our manuscript as follows;

Xylonate dehydratase in the Dahms pathway is a rate-limiting enzyme, and another isozyme such as E. coli yagF with higher enzyme activity [39] would be useful for efficient utilization of xylose.

(Please, see page 15, line 57-59)

2. Pg. 27 line 467, 'significantly contribute to the production of other chemicals with high yields'. Please change this to 'significantly contribute to the production of other non-shikimate pathway chemicals with high yields'.

Response

As you pointed out, we revised our manuscript as follows;

Therefore, it is expected that the PMPE strain will significantly contribute to the production of other non-shikimate pathway chemicals with high yields.

(Please, see page 15, line 57)

3. Pg. 27 line 460, Using Weimberg pathway….glyoxylate. Support this sentence with reference.

Response

As you pointed out, we revised our manuscript as follows;

Using Weimberg pathway is an alternative option because this pathway supplies α-ketoglutarate from xylose without producing glyoxylate[54].

(Please, see page 15, line 57)

4. Pg 23, line 399, Please rewrite the subsection or separate it in two, one for tyrosine and other one for 1,2-propanediol.

Response

As you pointed out, we revised our manuscript as follows;

Production of other target metabolites using PMPE strain

(Please, see page 15, line 57)
